# Comparative Efficacy of Glucosamine-Based Combination Therapies in Alleviating Knee Osteoarthritis Pain: A Systematic Review and Network Meta-Analysis

**DOI:** 10.3390/jcm13237444

**Published:** 2024-12-06

**Authors:** Dewan Md. Sumsuzzman, Zeeshan Ahmad Khan, Jin Ho Jung, Yunkyung Hong, Won Jong Yang, Kanghui Park, Hong Jin Choi, Ok Chan Jeong, Sang Jin Kim, Yonggeun Hong

**Affiliations:** 1 Department of Physical Therapy, College of Healthcare Medical Science & Engineering, Gimhae 50834, Republic of Korea; dewanpavelpharm@gmail.com (D.M.S.); acezeeshan@live.com (Z.A.K.); 2 Research Center for Aged-life Redesign (RCAR), Inje University, Gimhae 50834, Republic of Korea; 3 Biohealth Products Research Center (BPRC), Inje University, Gimhae 50834, Republic of Korea; 4 Department of Rehabilitation Science, Graduate School of Inje University, Gimhae 50834, Republic of Korea; 5 Department of Neurology, Busan Paik Hospital, Inje University College of Medicine, Busan 47392, Republic of Korea; sweetmasil@gmail.com (J.H.J.); jsk502@inje.ac.kr (S.J.K.); 6 Dementia and Neurodegenerative Disease Research Center, Inje University, Busan 47392, Republic of Korea; 7 Medical Device Commercialization Support Team, Gimhae Biomedical Industry Promotion Agency (GBIA), Gimhae 50969, Republic of Korea; dangmoo777@naver.com; 8 Department of Rehabilitation Medicine, Medical Corporation Daegu Medical Foundation The K Hospital, Daegu 47392, Republic of Korea; louis.yang.rm@gmail.com; 9 Department of Physical Therapy, Busan Health University, Busan 49318, Republic of Korea; djpt2014@naver.com; 10 Department of Digital Anti-Aging Healthcare, Inje University, Gimhae 50834, Republic of Korea; hjchoi@inje.ac.kr (H.J.C.); memsoku@inje.ac.kr (O.C.J.); 11 Department of Biomedical Engineering, College of Healthcare Medical Science & Engineering, Gimhae 50834, Republic of Korea

**Keywords:** glucosamine, combination therapy, knee osteoarthritis, systematic review, network meta-analysis

## Abstract

**Background:** The lack of definitive scientific evidence sustains uncertainty about the efficacy of glucosamine and its combination therapies for knee osteoarthritis (KOA), contributing to an ongoing debate among clinical practice guidelines and healthcare practitioners. This systematic review and network meta-analysis (NMA) aimed to identify the most effective glucosamine combination therapy for KOA patients. **Methods:** Frequentist random-effects models were employed for this NMA, with standardized mean differences (SMDs) and 95% confidence intervals (CIs) calculated for primary outcomes. We incorporated an SMD value of 0.40 as a minimum clinically important difference (MCID) to interpret the pain outcome. Confidence in evidence was evaluated using CINeMA. **Results:** Thirty randomized controlled trials (RCTs) covering 5265 patients were included. Glucosamine with omega-3 (G + omega-3, SMD –2.59 [95% CI –4.42 to –0.75], moderate quality) and glucosamine with ibuprofen (G + ibuprofen, SMD –2.27 [95% CI –3.73 to –0.82], moderate quality) significantly reduced overall pain compared to placebo. Similarly, glucosamine + chondroitin sulfate + methylsulfonylmethane showed effectiveness in pain reduction (SMD –2.25 [95% CI –3.84 to –0.67], low-quality). None of the other interventions met the MCID threshold for overall pain reduction. Moreover, clustered ranking results showed that glucosamine with omega-3 interventions was more effective than others in reducing overall pain and adverse events. **Conclusions:** For KOA, combining glucosamine with omega-3 and ibuprofen effectively reduces pain and may lower NSAID side effects, improving treatment guidelines and decision-making for better patient care.

## 1. Introduction

Osteoarthritis (OA) remains a major global health issue, with its prevalence rising with age and varying across different geographic regions and anatomic sites [1]. The knee is the most commonly affected anatomic site, accounting for over 80% of the total OA burden [2], and affects at least 19% of American adults aged 45 years and older [3]. Arguably, pain from knee osteoarthritis (KOA) significantly impacts healthcare costs and is a primary driver for seeking medical consultation [4]. Effective management of KOA is therefore crucial not only for improving the patient’s quality of life but also for reducing the associated economic burden.

Managing KOA pain mainly relies on NSAIDs, but their prolonged use raises the risk of gastrointestinal and cardiovascular adverse events [5]. In contrast, glucosamine is a safe and effective dietary supplement for alleviating KOA pain and is frequently recommended to modify its clinical and radiological course. For instance, in Australia, 85.2% of physicians and 94.7% of community pharmacists advise their patients to use glucosamine for relieving OA symptoms [6]. However, the American College of Rheumatology conditionally does not recommend the use of glucosamine for treating KOA [7]. Due to varied conclusions in numerous clinical trials and systematic reviews on glucosamine for KOA [8,9,10,11,12], current clinical practice guidelines (CPGs) do not advocate its use. Despite discrepancies between medical practitioners and CPGs, alongside inconsistent conclusions from systematic reviews, glucosamine remains a topic of interest. Additionally, Consegic Business Intelligence forecasts that the glucosamine supplement market will grow to USD 1082.65 million by 2030, with a 5.8% annual increase from USD 693.50 million in 2022 [13]. North America, leading with a 36.50% share at USD 253.13 million in 2022, is expected to reach USD 397.87 million by 2030 [13]. This growth projection underscores ongoing interest and potential market expansion, despite the conflicting stances on glucosamine within the medical and research communities.

One recent scoping review shows that combining glucosamine with NSAIDs yields better pain reduction and adverse event management in KOA, indicating its potential for a multimodal treatment approach [14]. This approach aligns with numerous clinical trials showing the benefits of combining glucosamine with other interventions like omega-3 [15], chondroitin [16], hyaluronic acid [17], and curcumin [18] to alleviate KOA symptoms. However, unresolved questions persist: What is the most effective glucosamine combination therapy, and are there definite benefits in short-term and long-term usage for KOA patients?

A traditional pairwise meta-analysis only compares two interventions at a time, limiting the ability to draw broader conclusions about the relative effectiveness of different combination therapies. In contrast, a network meta-analysis (NMA) integrates both direct and indirect evidence from all available randomized controlled trials (RCTs), enabling comparisons of multiple treatments simultaneously. This approach provides more accurate estimates, enhances statistical power, and allows for consolidated comparisons of alternative therapies. Addressing this gap in research, this study employs an NMA to evaluate the comparative effectiveness, relative rankings, and safety of various interventions alongside glucosamine for managing KOA-related pain and adverse events. The findings aim to inform guideline recommendations and evidence-based decision-making in clinical practice.

## 2. Materials and Methods

### 2.1. Search Strategy and Selection Criteria

We registered our protocol on PROSPERO (CRD42022331863) and reported our study following the Preferred Reporting Items for Systematic Reviews and Meta-analyses and the extension statement for network meta-analysis (PRISMA-NMA) [19]. We searched Medline (Ovid), Embase, Cochrane Library, Clinicaltrials.gov, and the International Clinical Trials Registry Platform (ICTRP) from inception to 30 November 2023 and updated the search results in March 2024. We also manually chased citations for relevant studies. No publication date and language restrictions were applied. The detailed search strategy is shown in Supplementary Table S1.

We included RCTs that evaluate the effectiveness of glucosamine in combination with different pharmacological interventions for pain in adult patients with KOA. We included studies that evaluated these interventions compared with a placebo, glucosamine alone, or another active comparator. In the case of multiple-arm RCTs, we employed a multivariate meta-analysis framework with data augmentation to account for correlations between treatment effects within the same study. Each treatment arm was modeled separately, with one arm designated as the reference, ensuring transparent and robust comparisons across treatments in the NMA. Our pre-defined primary outcome was pain, with data extracted from the scale highest on the following hierarchical list: (1) Western Ontario and McMaster Universities Osteoarthritis Index (WOMAC) pain subscore; (2) Visual Analogue Scale (VAS). For overall pain outcome, we extracted the longest follow-up data to avoid unit analysis errors [20]. Short-term (≤3 months) and long-term (>3 months) endpoints were also extracted to generate more clinically important results. Our secondary outcome was any adverse events, with data analyzed for the same time points as those for overall pain. We excluded studies combining glucosamine with non-pharmacological treatments and herbal or dietary supplements due to the potential for confounding effects. For example, non-pharmacological interventions, such as exercise, could independently affect pain and function in KOA, complicating the isolation of glucosamine’s effect, while the lack of robust clinical evidence for herbal supplements and dietary interventions further justified their exclusion. Supplementary Table S2 provides details on the exclusion criteria.

### 2.2. Data Extraction

Four reviewers independently evaluated each trial using the double-entry method to verify data accuracy for data extraction. Disagreements were resolved through consultation with a senior scholar. We extracted trial characteristics, such as study design, center, funding, and follow-up; intervention characteristics, such as treatment arm, dose, and the number of patients in each treatment arm; participant characteristics, such as mean age, percentage of females, disease severity, duration of diseases, and body mass index (BMI); and type of outcomes. Results based on the intention-to-treat principle were extracted whenever possible.

### 2.3. Risk-of-Bias Assessment

Two authors independently assessed the methodological quality of the included trials using the Cochrane risk-of-bias assessment tool. Each item was rated as having a low, high, or unclear risk of bias. Disagreements were determined with another reviewer.

### 2.4. Credibility of Evidence

We assessed the overall credibility of the evidence in the network for the primary outcomes using the Confidence in Network Meta-Analysis (CINeMA) tool [21], which summarizes the level of concern for each comparison based on the contributions of the direct comparisons to the NMA estimation.

### 2.5. Statistical Analysis and Evaluation of Assumptions

We used a frequentist NMA to analyze overall pain and adverse events using Stata software version 16.0. As outcomes were measured on different scales, we calculated standardized mean differences (SMDs) with 95% confidence intervals (CIs) for continuous data. We defined effect sizes as small, moderate, or large based on the following criteria: small for SMD between 0.20 and 0.49, moderate for SMD between 0.50 and 0.79, and large for SMD of 0.80 or higher [22]. We determined the minimum clinically important difference (MCID) for pain outcome to be an SMD of 0.40, reflecting the smallest change perceived as beneficial by patients [23]. We estimated odds ratio (OR) and 95% CIs for adverse events. We presented the interval plot results and league tables for all interventions against placebo and estimated mean rank and relative treatment rankings using SUCRA values [24]. Furthermore, a cluster ranking plot was generated to evaluate the comprehensive ability of treatments to reduce overall pain and adverse events [25]. Finally, the *metan* command was used for the pairwise meta-analyses. We conducted subgroup analyses to investigate the impact of different endpoints on pain outcomes in OA. We assessed the basic transitivity assumption by comparing potential effect modifiers and visual inspection of patient characteristics. To assess inconsistency between direct and indirect evidence, we used three methods: a loop-specific approach [25,26], a side-splitting method (0.10 > *p*; favoring consistency) [27], and a global inconsistency approach (Wald test 0.10 > *p*; favoring consistency) [28]. We also used comparison-adjusted funnel plots to assess publication bias [25].

## 3. Results

### 3.1. Search Results and Study Characteristics

**Figure 1:**
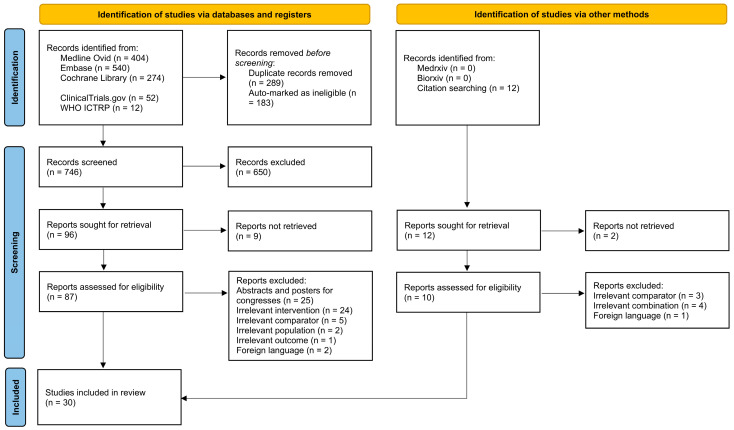
Study flow diagram for evidence source and selection process.

A flowchart outlining the study selection procedure is presented in Figure 1. A total of 1282 records were retrieved from electronic databases and registry searches, and 30 studies involving 5265 knee OA patients with pain were included in the systematic review. Of these, 28 articles were identified from electronic sources, while 2 were identified from other sources such as reference lists and forward citation searching. The screening process involved 746 records for titles and abstracts and 88 full-text articles for eligibility after removing duplicates.

**Figure 2:**
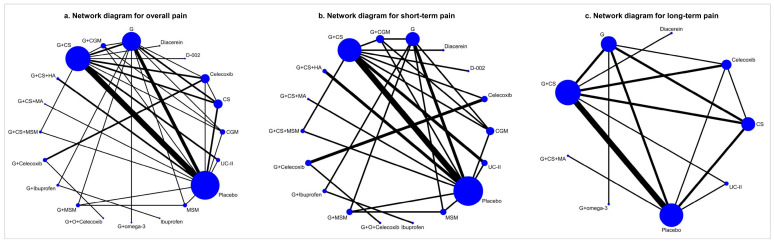
Network plot of studies included in network meta-analysis for overall pain (**a**), short-term pain (**b**), and long-term pain (**c**). The size of the nodes is proportional to the number of studies evaluating each intervention, and the thickness of the edges is proportional to the number of randomly assigned patients contributing to direct comparisons. G, glucosamine; CS, chondroitin sulfate; UC-II, undenatured type II collagen; D-002, an inhibitor of both cyclooxygenase and 5-lipoxygenase activities; MSM, methylsulfonylmethane; G + CS; glucosamine + chondroitin sulfate; G + Celecoxib, glucosamine + celecoxib; G + Ibuprofen, glucosamine + ibuprofen; G + omega-3, glucosamine + omega-3; G + CS + HA, glucosamine + chondroitin sulfate + hyaluronic acid; G + MSM, glucosamine + methylsulfonylmethane; +CS + MSM, glucosamine + chondroitin sulfate + methylsulfonylmethane; G + CGM, glucosamine + curcumin derivatives; G + O + Celecoxib, glucosamine + ozone + celecoxib; G + CS + MA, glucosamine + chondroitin sulfate + manganese ascorbate.

Six out of thirty studies were excluded from this NMA due to the primary outcome, four due to disconnectedness [29,30,31,32], and two due to not meeting the transitivity assumption [33,34]. The characteristics of the 24 RCTs included in this NMA are presented in Supplementary Table S3, with most studies being non-industry-funded and conducted in single-center settings, mainly in Asia. Mean age, percentage of female patients, BMI, and disease severity were similar across studies assessing pain. The domain level and overall risk of bias judgments for pain are presented in Supplementary Figures S1 and S2. Of the 24 studies included in this NMA, 8 were judged to have some concerns (33.33%), 2 had a high risk of bias (8.33%), and 14 had a low risk of bias (58.33%) for overall pain. The unclear risk of bias was mainly due to insufficient information on the blinding of participants and personnel (33.33%; 8/24) and allocation concealment (33.33%; 8/24). The high risk of bias was mostly related to other sources of bias domain due to industry-funded studies and/or missing conflict of interest statements (70.83%; 17/24). Figure 2 shows the network plots of available comparisons for pain. Glucosamine in combination with chondroitin sulfate (G + CS) vs. placebo and glucosamine alone (G) vs. placebo were the most common interventions for overall pain and short-term and long-term pain. Regarding combination therapies for overall pain, G + CS + HA and G + CS + MA were compared only with placebo and not with any other interventions or controls; therefore, no closed loops were identified for these combinations. Similarly, G + omega-3 was only compared with G, without any other interventions or control groups. A comparable network geometry was observed for both short-term and long-term pain outcomes. No important clinical differences in the distributions of most effect modifiers were observed in the network. The transitivity assumption for the duration of the disease could not be statistically evaluated due to the small number of available data. Tests for the inconsistency of some loops involving G + CGM intervention were positive for overall pain and short-term pain, but there was no evidence of inconsistency for long-term pain. The global Wald test for statistical incoherence was not significant for overall pain, short-term pain, and long-term pain. Detailed information regarding network geometries, transitivity, and coherence are provided in Supplementary Tables S4–S7, and Supplementary Figures S3–S11.

### 3.2. Sensitivity Analysis for Statistical Incoherence

We conducted a sensitivity analysis to address inconsistencies between direct and indirect evidence identified by the loop-specific and side-splitting methods. For overall pain, the 95% CI of five loops did not overlap the null, with three involving the glucosamine–curcumin derivatives (G + CGM) combination, indicating that the test for the inconsistency of these loops was positive (Supplementary Figure S9). Similar results were observed using the side-splitting method: the *p*-values between direct and indirect evidence were generally consistent, with minor differences related to the G + CGM intervention (Supplementary Table S4). The degree of inconsistency in short-term pain was similar to that seen in overall pain (Supplementary Figure S10 and Table S5). The inconsistencies were minor and mainly related to the G + CGM intervention for overall pain and short-term pain outcomes. After excluding the sources of inconsistency, the *p*-values between direct and indirect evidence were consistent in our sensitivity analysis (Supplementary Tables S8 and S9). No evidence of inconsistency was observed for long-term pain using both the loop-specific and side-splitting methods (Supplementary Figure S11 and Table S6). The global Wald test for statistical incoherence was also non-significant for overall pain (Chi2 = 1.02, *p* = 0.9998) and short-term pain (Chi2 = 0.08, *p* = 0.9999) (Supplementary Table S10).

### 3.3. Relative Effects and Ranking of Interventions for Pain

**Figure 3:**
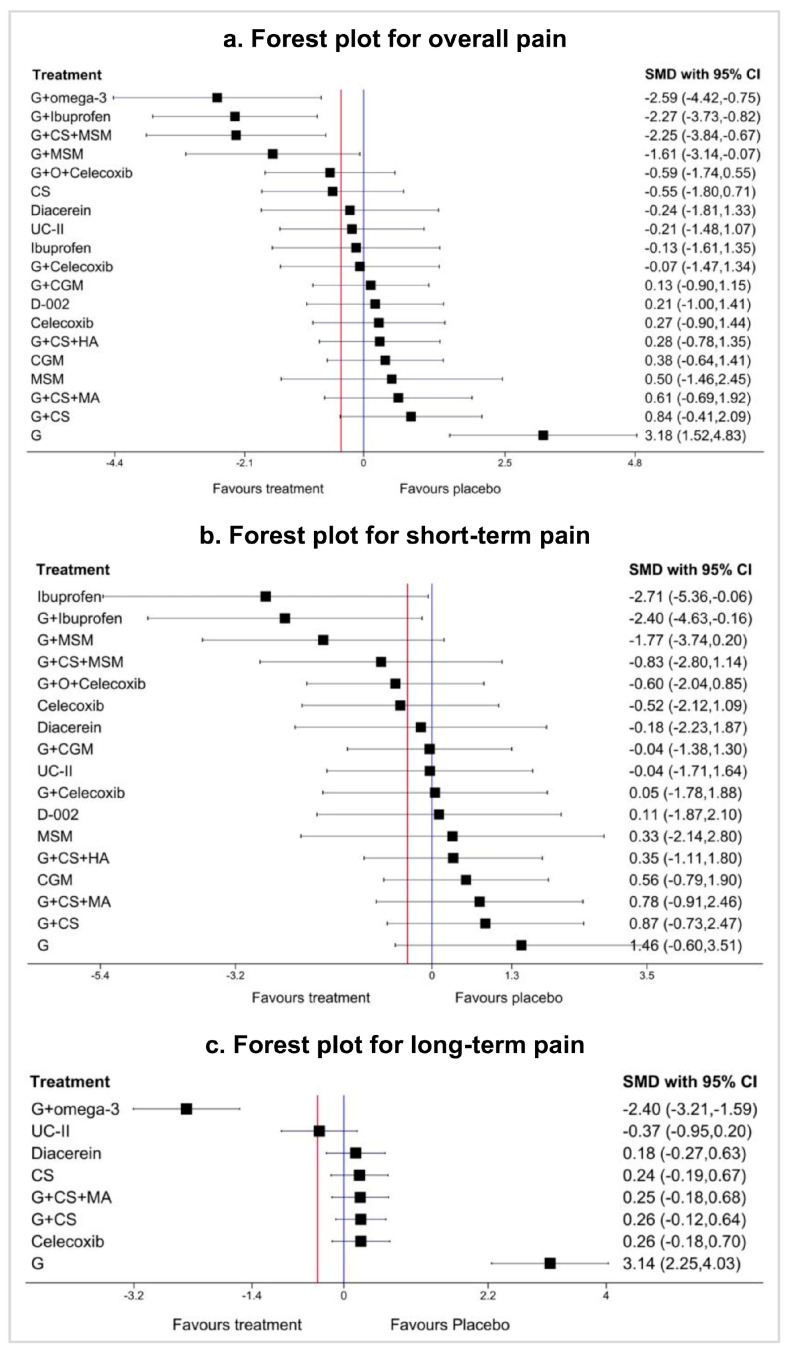
Forest plots of network meta-analysis for overall pain (**a**), short-term pain (**b**), and long-term pain (**c**) compared with placebo, ordered according to treatment effect size. The purple vertical line represents the line of no effect, the red vertical line indicates the MCID, and the black square represents the effect size. When the 95% CI crosses the MCID line, it indicates that the effectiveness of the intervention is uncertain. MCID, minimum clinically important difference; SMD, standardized mean difference; 95% CI, 95% confidence interval; G + omega-3, glucosamine + omega-3; G + Ibuprofen, glucosamine + ibuprofen; G + CS + MSM, glucosamine + chondroitin sulfate + methylsulfonylmethane; G + MSM, glucosamine + methylsulfonylmethane; G + O + Celecoxib, glucosamine + ozone + celecoxib; CS, chondroitin sulfate; UC-II, undenatured type II collagen; G + Celecoxib, glucosamine + celecoxib; D-002, an inhibitor of both cyclooxygenase and 5-lipoxygenase activitiy; G + CS + HA, glucosamine + chondroitin sulfate + hyaluronic acid; CGM, curcumin derivatives; MSM, methylsulfonylmethane; G + CS + MA, glucosamine + chondroitin sulfate + manganese ascorbate; G + CS; glucosamine + chondroitin sulfate; G, glucosamine.

**Figure 4:**
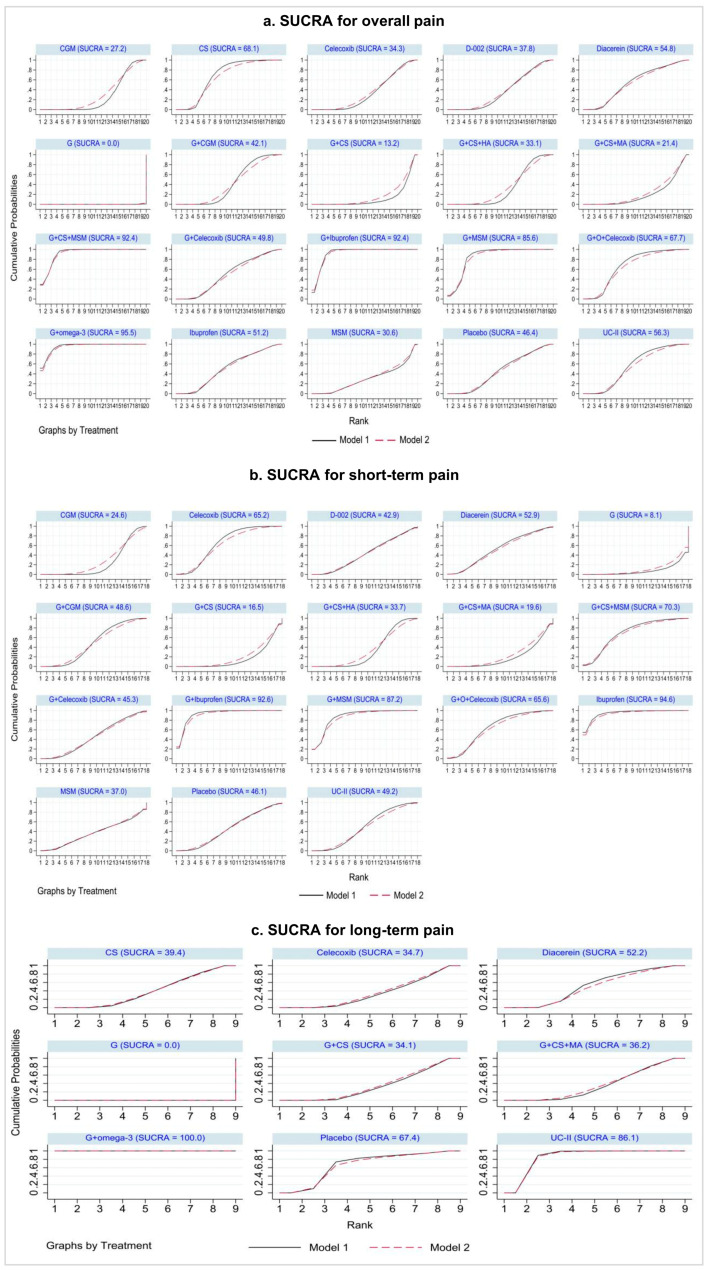
Rankograms of treatments for overall pain (**a**), short-term pain (**b**), and long-term pain (**c**) included in the network meta-analysis. G + omega-3, glucosamine + omega-3; G + Ibuprofen, glucosamine + ibuprofen; G + CS + MSM, glucosamine + chondroitin sulfate + methylsulfonylmethane; G + MSM, glucosamine + methylsulfonylmethane; G + O + Celecoxib, glucosamine + ozone + celecoxib; CS, chondroitin sulfate; UC-II, undenatured type II collagen; G + Celecoxib, glucosamine + celecoxib; D-002, an inhibitor of both cyclooxygenase and 5-lipoxygenase activity; G + CS + HA, glucosamine + chondroitin sulfate + hyaluronic acid; CGM, curcumin derivatives; MSM, methylsulfonylmethane; G + CS + MA, glucosamine + chondroitin sulfate + manganese ascorbate; G + CS; glucosamine + chondroitin sulfate; G, glucosamine.

In the NMA of overall pain, 24 studies with 20 interventions (59 arms) were included. Glucosamine with omega-3 (G + omega-3, SMD −2.59 [95% CI −4.42 to −0.75], moderate quality), glucosamine with ibuprofen (G + ibuprofen, SMD −2.27 [95% CI −3.73 to −0.82], moderate quality), and glucosamine + chondroitin sulfate + methylsulfonylmethane (G + CS + MSM, −2.25 [95% CI −3.84 to −0.67], low quality) showed a large and clinically important effect on reducing pain compared with placebo (Figure 3a). Glucosamine with methylsulfonylmethane (G + MSM, −1.61 [95% CI −3.14 to −0.07], moderate quality) also showed a large effect, but the 95% CI crossed the MCID line. We present confidence in the findings of the NMA for overall pain using the CINeMA approach (Supplementary Table S11). Pairwise meta-analysis results are also presented in Supplementary Table S12. A sensitivity analysis excluding sources of inconsistency produced similar effect estimates (Supplementary Figure S12). G + omega-3, G + ibuprofen, and G + CS + MSM were the top interventions for overall pain (SUCRA values: 95.5, 92.4, and 92.4, respectively; mean rank: 1.9, 2.4, and 2.4, respectively; Supplementary Table S13; Figure 4a). Supplementary Table S14 provides the effect estimates for studies excluded from the NMA for overall pain.

For short-term pain, 20 studies with 18 interventions (46 arms) were included in the NMA, and ibuprofen (SMD −2.71 [95% CI −5.36 to −0.06], low quality), G + ibuprofen (SMD −2.40 [95% CI −4.63 to −0.16] moderate quality), and G + MSM (SMD −1.77 [95% CI −3.74 to 0.20] low quality) showed a large effect on reducing pain compared with placebo (Figure 3b). However, their 95% CI extended beyond the MCID line. We provide confidence in the NMA’s findings for short-term pain in Supplementary Table S15 and present the results of pairwise and network evidence from the certainty assessment for short-term pain in Supplementary Table S16.

Sensitivity analysis produced similar effect estimates (Supplementary Figure S13), and the most highly ranked interventions were ibuprofen, G + ibuprofen, and G + MSM (Supplementary Table S17). Rankograms for short-term pain are presented in Figure 4b. The effect estimated for studies excluded from the NMA for short-term pain is shown in Supplementary Table S18.

Among the nine interventions for long-term pain tested in the NMA, with nine studies (24 arms), only G + omega-3 (SMD −2.40 [95% CI −3.21 to −1.59], moderate quality) had a large and clinically important effect on reducing pain compared to placebo (Figure 3c), ranking the highest among the interventions. Confidence in NMA findings for pain is presented in Supplementary Table S19, and pairwise and network evidence for long-term pain with certainty assessment using CINeMA is presented in Supplementary Table S20. G + omega-3 was the most highly ranked intervention for long-term pain based on SUCRA values and mean rank (Supplementary Table S21), and rankograms are presented in Figure 4c. Effect estimates for excluded studies are presented in Supplementary Table S22.

### 3.4. Relative Effects and Ranking of Interventions for Adverse Events

In the NMA of adverse-events, 17 studies with 13 interventions (42 arms) were included (Supplementary Figure S14). G + omega-3 had the lowest odds of adverse events (OR 0.17 [95% CI 0.02 to 1.28]), while celecoxib alone had the highest odds (OR 1.24 [95% CI 0.25 to 6.18]) (Supplementary Figure S15). Pairwise and network evidence for adverse events is presented in Supplementary Table S23. G + omega-3 was the most highly ranked intervention for minimizing adverse events (SUCRA = 89.3, mean rank = 2.4, Supplementary Table S24). Rankograms for adverse events are presented in Supplementary Figure S16. Transitivity assumption and statistical incoherence results are also presented in Supplementary Tables S25 and S26 and Supplementary Figures S17–S19.

### 3.5. Clustered Ranking

**Figure 5:**
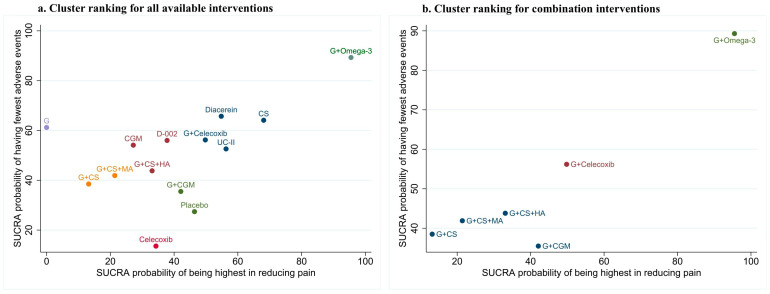
Cluster ranking plot for overall pain and adverse events (**a**) for all available treatments and (**b**) for glucosamine combination interventions. The treatment in the upper right corner is considered to perform well for both outcomes. Different plotting symbols represent different clusters of treatments. G + Ibuprofen, glucosamine + ibuprofen; CS, chondroitin sulfate; G, glucosamine; G + Celecoxib, glucosamine + celecoxib; D-002, an inhibitor of both cyclooxygenase and 5-lipoxygenase activities; CGM, curcumin derivatives; UC-II, undenatured type II collagen; G + CS + HA, glucosamine + chondroitin sulfate + hyaluronic acid; G + CS + MA, glucosamine + chondroitin sulfate + manganese ascorbate; G + CS; glucosamine + chondroitin sulfate; G + CGM, glucosamine + curcumin derivative.

Using a cluster analysis of SUCRA values for reducing overall pain and adverse events, we generated two clustered ranking plots: one that includes all available treatments (Figure 5a) and one specifically on glucosamine combination interventions (Figure 5b). G + omega-3 interventions were found to be more effective in reducing overall pain and adverse events compared to other interventions.

### 3.6. Evaluation of Small-Study Effects

Small-study effects for pain and adverse event outcomes were visually inspected using comparison-adjusted funnel plots. The results showed no significant evidence of small-study bias, as indicated by a symmetrical funnel plot (Supplementary Figures S20–S23) and Egger’s regression test (overall pain: *p* = 0.897; short-term pain: *p* = 0.996; long-term pain: *p* = 0.440; and adverse events: *p* = 0.742).

## 4. Discussion

In this systematic review and NMA, 30 RCTs involving 5265 KOA patients were analyzed to compare the effectiveness and adverse events of glucosamine combined with various pharmacological interventions, compared to placebo, glucosamine alone, or other active comparators. Among the interventions tested in this study for reducing overall pain in KOA patients, G + omega-3, G + ibuprofen, and G + CS + MSM were found to be the most effective, with G + omega-3 and G + ibuprofen showing moderate certainty and G + CS + MSM showing low certainty. In order of effectiveness, G + omega-3 ranked highest, followed by G + ibuprofen and then G + CS + MSM. For short-term pain, ibuprofen, G + ibuprofen, and G + MSM were the most effective interventions, with moderate certainty that G + ibuprofen and G + MSM reduce short-term pain. G + omega-3 was found to be the only effective intervention for reducing long-term pain, and G + omega-3 interventions resulted in the lowest odds of adverse events. Celecoxib alone resulted in the highest odds of adverse events, while G + celecoxib was less associated with adverse events than celecoxib alone. In summary, G + omega-3 is the most effective intervention for reducing pain and adverse events in KOA patients, and incorporating glucosamine with pharmacological interventions may be a better therapeutic choice than using pharmacological interventions alone.

We utilized the NMA design to evaluate all available combinations of glucosamine and pharmacological interventions for managing pain in KOA. This allowed us to simultaneously compare and rank competing interventions within one coherent treatment network to determine the comparative effectiveness of these interventions with glucosamine. We performed a comprehensive literature search and utilized at least four blinded authors for study selection, data extraction, and quality assessment to minimize bias and transcription errors. Our study’s strength lies in its ability to synthesize direct and indirect evidence to determine the comparative effectiveness of these interventions with a clinically relevant MCID.

In our NMA, we relied on the transitivity assumption, which assumes that indirect comparisons can accurately estimate unobserved head-to-head comparisons [35]. Our analysis showed no clinically important differences in age and percentage of females among the trials, supporting this assumption. However, we could not fully evaluate the duration of disease due to limited data. We excluded studies that did not meet the transitivity assumption [33,34] or were disconnected from the main network [29,30,31,32]. We resolved inconsistency by excluding conflicting sources and confirmed consistent evidence. For example, we conducted a sensitivity analysis by excluding studies with a high risk of bias for overall pain and short-term pain, as detailed in the quality assessment section (Supplementary Table S27), and the results remained consistent with those from the main analysis, highlighting the robustness of our findings (Supplementary Figure S24).

Several published systematic reviews have assessed the effectiveness of glucosamine combination strategies for pain in KOA patients [36,37,38]. Some of these reviews utilized an NMA approach [39,40,41]. However, all of them focused only on the combination of G + CS. This is the first systematic review and NMA to evaluate all available pharmacological interventions used in combination with glucosamine to alleviate pain in KOA patients. Furthermore, the previous reviews and NMAs did not differentiate or separately analyze the short-term and long-term effects of these combination strategies on reducing pain in KOA. Prior NMAs have presented conflicting findings on the effectiveness of G + CS treatments for KOA [39]. Our study aligns with Wandel et al.’s NMA, conducted in 2010 [39], which found that G + CS did not reduce pain compared to a placebo. However, we disagree with the results of another NMA [41] and do not recommend G + CS for OA treatment due to several reasons. Firstly, the interpretation of treatment comparisons lacked consideration of MCID. Secondly, the evaluation of confidence in the NMA’s results was absent, rendering them unreliable and inconsistent with clinical practice guidelines. In a previous RCT, G + omega-3 was found to be clinically and statistically superior to glucosamine alone in reducing pain by ≥80% of the WOMAC pain score, possibly due to its synergistic effects on osteoarthritic joints [42]. Our findings are consistent with the literature, as G + omega-3 was found to be the most effective intervention for reducing pain in KOA patients.

International guidelines recommend NSAIDs for managing OA pain [7,43], but they often lack efficacy and have side-effects [44,45]. Combining glucosamine with NSAIDs improves pain, reduces side effects, and enhances quality of life [46]. Our study confirms that G + ibuprofen is more effective at reducing pain in KOA than ibuprofen alone, and G + celecoxib has demonstrated superiority in pain reduction and reducing adverse events over celecoxib alone. However, both interventions extended beyond the MCID line, possibly because the efficacy of celecoxib is dose-dependent, and a minimum 12-week treatment duration is necessary to sustain pain reduction in OA [47]. Two of the four studies [48,49] in our NMA assessed G + celecoxib efficacy at 6 and 8 weeks, respectively. Therefore, we recommend that G + celecoxib be given to OA patients for at least 12 weeks to optimize pain outcomes.

Glucosamine has primarily been studied for its efficacy in reducing joint pain in OA, but its potential effects may extend beyond symptom relief. Given the systemic nature of OA, glucosamine may not only alleviate pain but also modulate key biological markers—such as inflammation and cartilage turnover—that are critical to disease progression [50,51,52]. While our study focuses primarily on pain management, these broader effects underscore glucosamine’s multifaceted therapeutic potential in knee OA. Furthermore, chronic pain in OA often disrupts circadian rhythms, leading to sleep disturbances that can further exacerbate pain and reduce quality of life [53,54]. Despite the discovery of various circadian modulators, their clinical use has been limited, possibly due to a lack of high-quality evidence and pharmacokinetic uncertainties [55]. Repurposing glucosamine may offer additional benefits to knee OA patients, as it could indirectly reduce pain by regulating circadian rhythms [55]. When considering the pathogenesis of OA, the gut microbiome is less likely pronounced, but it may play a role in pain modulation through mitigating inflammation. In a previous clinical trial, glucosamine significantly attenuated knee joint pain, stiffness, and improved joint flexibility [56]. However, exploring this potential pathway could offer valuable insights into how glucosamine may exert systemic anti-inflammatory effects and contribute to pain relief.

The potential of combining glucosamine with pharmacological interventions for pain management in KOA patients remains unclear. Our NMA addresses this gap, identifying effective and ineffective interventions to inform guidelines and support decision-making. However, the variability in methodology and small sample sizes in KOA pain studies necessitate high-quality RCTs. Additionally, establishing optimal dosing and duration of NSAIDs with glucosamine is necessary. Improved reporting guidelines for adverse events related to combined interventions would enhance data accuracy and clinical risk–benefit assessment. Topical NSAIDs can provide musculoskeletal pain relief and minimize systemic side effects [57]. Future studies are recommended to investigate the efficacy, safety, and cost-effectiveness of topically applied NSAIDs with glucosamine for KOA pain relief. Although we searched key biomedical databases and clinical trial registries, we did not include subject-specific or regional databases, nor gray literature (e.g., CINAHL, LILACS), which may have resulted in the omission of relevant studies and introduced publication bias [58]. To mitigate this limitation and provide a more complete picture of the evidence, future research should incorporate these sources. Finally, the absence of direct comparisons between several glucosamine combination therapies—G + CS + HA, G + CS + MA, and G + omega-3—and other interventions, the evidence for these combinations remains primarily indirect, somewhat limiting the strength of our conclusions about their relative effectiveness. Hence, future research should prioritize investigating these combinations to assess their potential synergistic effects in improving pain management and reducing inflammation in OA patients.

Our study, using NMA methodology, found that G + omega-3 and G + ibuprofen are the most effective for managing osteoarthritis pain. G + omega-3 showed superior efficacy and fewer adverse events compared to other combinations. Additionally, adding glucosamine to NSAIDs may reduce adverse events. However, we found that the G + CS combination does not provide a clinically significant pain reduction in mild-to-moderate KOA patients. Our estimates have low-to-moderate certainty and can guide decision-making. The cost-effectiveness of these combination therapies is uncertain and warrants further investigation.

## 5. Conclusions

Omega-3 and ibuprofen seem to be the most effective pharmacological interventions with glucosamine for osteoarthritis pain. Our findings also suggest that glucosamine with omega-3 was more effective than others in reducing both pain and adverse events. Furthermore, adverse events from NSAIDs in pain management might be reduced by adding glucosamine. We are also confident that the G + CS combination does not reduce clinically significant pain in mild-to-moderate KOA patients. With the NMA approach and focusing on combined therapy for pain, our study provided comprehensive evidence on relative treatment effects. Many of our estimates were based on evidence of low-to-moderate certainty and can inform decision-making. In terms of cost-effectiveness, we are uncertain whether the proposed combination therapies are easy to implement in clinical practice. Further studies regarding the cost involved with these combined therapy modalities would be worthwhile.

## Data Availability

All data analyzed in this study are available in this published article and Supplementary Materials. Further inquiries can be directed to the corresponding author (Yonggeun Hong; yonghong@inje.ac.kr) upon reasonable request.

## Supplementary Materials

The following supporting information can be downloaded at https://www.mdpi.com/article/10.3390/jcm13237444/s1, Table S1. Search strategies, Table S2. Studies excluded at full text-level, Table S3. Characteristics of studies included in network meta-analysis, Table S4. Results of a side-splitting method for overall pain, Table S5. Results of a side-splitting method for short-term pain, Table S6. Results of a side-splitting method for long-term pain, Table S7. Results of global inconsistency tests for the primary analyses, Table S8. Sensitivity analysis of the side-splitting method for statistical inconsistency in overall pain, Table S9. Sensitivity analysis of the side-splitting method for statistical inconsistency in short-term pain, Table S10. Sensitivity analysis of global inconsistency tests for the primary analyses, Table S11. Confidence rating for overall pain using CINeMA, Table S12. Pairwise and network meta-analysis results for overall pain, Table S13. SUCRA and mean rank results for overall pain, Table S14. Studies excluded from the network meta-analysis for overall pain: Table S15. Confidence rating for short-term pain using CINeMA, Table S16. Pairwise and network meta-analysis results for short-term pain, Table S17. SUCRA and mean rank results for short-term pain, Table S18. Studies excluded from the network meta-analysis for short-term pain, Table S19. Confidence rating for long-term pain using CINeMA, Table S20. Pairwise and network meta-analysis results for long-term pain, Table S21. SUCRA and mean rank results for long-term pain, Table S22. Studies excluded from the network meta-analysis for long-term pain, Table S23. Pairwise and network meta-analysis results for adverse events, Table S24. Rank results for adverse events, Table S25. Results of a side-splitting method for adverse events, Table S26. Results of global inconsistency tests for the primary analyses. Table S27. The quality assessment included studies in the network meta-analysis. Figure S1. Risk of bias graph, Figure S2. Risk of bias summary, Figure S3. Mean age distribution in the network of interventions for overall pain outcome, Figure S4. Percentage of females in the network of interventions for overall pain outcome, Figure S5. Mean age distribution in the network of interventions for short-term pain outcome, Figure S6. Percentage of females in the network of interventions and short-term pain outcomes, Figure S7. Mean age distribution in the network of interventions and long-term pain outcomes, Figure S8. Percentage of females in the network of interventions for long-term pain outcomes, Figure S9. Inconsistency factors for overall pain of all trials, Figure S10. Inconsistency factors for short-term pain from 20 trials, Figure S11. Inconsistency factors for long-term pain from 9 trials, Figure S12. Sensitivity analysis to resolve the inconsistency for overall pain, Figure S13. Sensitivity analysis to resolve the inconsistency for short-term pain, Figure S14. Network plot for adverse events, Figure S15. Interval plot for adverse events, Figure S16. SUCRA ranking for adverse events, Figure S17. Mean age distribution in the network of interventions for adverse event outcomes, Figure S18. Percentage of females in the network of interventions for adverse events outcomes, Figure S19. Inconsistency factors for overall pain in all trials, Figure S20. Comparison-adjusted funnel plot for overall pain, Figure S21. Comparison-adjusted funnel plot for short-term pain, Figure S22. Comparison-adjusted funnel plot for long-term pain, Figure S23. Comparison-adjusted funnel plot for adverse events, Figure S24. Sensitivity analysis of network meta-analysis for overall pain and short-term pain.

## References

[1] Long H., Liu Q., Yin H., Wang K., Diao N., Zhang Y., Lin J., Guo A. (2022). Prevalence Trends of Site-Specific Osteoarthritis from 1990 to 2019: Findings from the Global Burden of Disease Study 2019. Arthritis Rheumatol..

[2] GBD 2021 Osteoarthritis Collaborators (2023). Global, Regional, and National Burden of Osteoarthritis, 1990–2020 and Projections to 2050: A Systematic Analysis for the Global Burden of Disease Study 2021. Lancet Rheumatol..

[3] Wallace I.J., Worthington S., Felson D.T., Jurmain R.D., Wren K.T., Maijanen H., Woods R.J., Lieberman D.E. (2017). Knee Osteoarthritis Has Doubled in Prevalence since the Mid-20th Century. Proc. Natl. Acad. Sci. USA.

[4] Neogi T. (2013). The Epidemiology and Impact of Pain in Osteoarthritis. Osteoarthr. Cartil..

[5] Kolasinski S.L., Neogi T., Hochberg M.C., Oatis C., Guyatt G., Block J., Callahan L., Copenhaver C., Dodge C., Felson D. (2020). 2019 American College of Rheumatology/Arthritis Foundation Guideline for the Management of Osteoarthritis of the Hand, Hip, and Knee. Arthritis Rheumatol..

[6] Sibbritt D., Adams J., Lui C.-W., Broom A., Wardle J. (2012). Who Uses Glucosamine and Why? A Study of 266,848 Australians Aged 45 Years and Older. PLoS ONE.

[7] Hochberg M.C., Altman R.D., April K.T., Benkhalti M., Guyatt G., McGowan J., Towheed T., Welch V., Wells G., Tugwell P. (2012). American College of Rheumatology 2012 Recommendations for the Use of Nonpharmacologic and Pharmacologic Therapies in Osteoarthritis of the Hand, Hip, and Knee. Arthritis Care Res..

[8] Towheed T., Maxwell L., Anastassiades T.P., Shea B., Houpt J., Welch V., Hochberg M.C., Wells G.A. (2005). Glucosamine Therapy for Treating Osteoarthritis. Cochrane Database Syst. Rev..

[9] Rabade A., Viswanatha G.L., Nandakumar K., Kishore A. (2024). Evaluation of efficacy and safety of glucosamine sulfate, chondroitin sulfate, and their combination regimen in the management of knee osteoarthritis: A systematic review and meta-analysis. Inflammopharmacology.

[10] Gallagher B., Tjoumakaris F.P., Harwood M.I., Good R.P., Ciccotti M.G., Freedman K.B. (2015). Chondroprotection and the Prevention of Osteoarthritis Progression of the Knee: A Systematic Review of Treatment Agents. Am. J. Sports Med..

[11] Eriksen P., Bartels E.M., Altman R.D., Bliddal H., Juhl C., Christensen R. (2014). Risk of Bias and Brand Explain the Observed Inconsistency in Trials on Glucosamine for Symptomatic Relief of Osteoarthritis: A Meta-Analysis of Placebo-Controlled Trials. Arthritis Care Res..

[12] Ogata T., Ideno Y., Akai M., Seichi A., Hagino H., Iwaya T., Doi T., Yamada K., Chen A.-Z., Li Y. (2018). Effects of Glucosamine in Patients with Osteoarthritis of the Knee: A Systematic Review and Meta-Analysis. Clin. Rheumatol..

[13] Intelligence C.B. Glucosamine Market Size, Share, Trends|Forecast to 2030. https://www.consegicbusinessintelligence.com/glucosamine-market.

[14] Veronese N., Ecarnot F., Cheleschi S., Fioravanti A., Maggi S. (2022). Possible Synergic Action of Non-Steroidal Anti-Inflammatory Drugs and Glucosamine Sulfate for the Treatment of Knee Osteoarthritis: A Scoping Review. BMC Musculoskelet. Disord..

[15] Sibbritt D., Lui C., Kroll T., Adams J. (2016). Prevalence of Glucosamine and Omega-3 Fatty Acid Use and Characteristics of Users among Mid-Age Women: Analysis of a Nationally Representative Sample of 10,638 Women. J. Nutr. Health Aging.

[16] Roman-Blas J.A., Castañeda S., Sánchez-Pernaute O., Largo R., Herrero-Beaumont G., CS/GS Combined Therapy Study Group (2017). Combined Treatment With Chondroitin Sulfate and Glucosamine Sulfate Shows No Superiority Over Placebo for Reduction of Joint Pain and Functional Impairment in Patients With Knee Osteoarthritis: A Six-Month Multicenter, Randomized, Double-Blind, Placebo-Controlled Clinical Trial. Arthritis Rheumatol..

[17] Wang S.-J., Wang Y.-H., Huang L.-C. (2021). The Effect of Oral Low Molecular Weight Liquid Hyaluronic Acid Combination with Glucosamine and Chondroitin on Knee Osteoarthritis Patients with Mild Knee Pain: An 8-Week Randomized Double-Blind Placebo-Controlled Trial. Medicine.

[18] Thomas J.V., Smina T.P., Khanna A., Kunnumakkara A.B., Maliakel B., Mohanan R., Krishnakumar I.M. (2021). Influence of a Low-Dose Supplementation of Curcumagalactomannoside Complex (CurQfen) in Knee Osteoarthritis: A Randomized, Open-Labeled, Active-Controlled Clinical Trial. Phytother. Res..

[19] Hutton B., Catalá-López F., Moher D. (2016). The PRISMA statement extension for systematic reviews incorporating network meta-analysis: PRISMA-NMA. Med. Clin..

[20] 4 Repeated Observations on Participants. https://handbook-5-1.cochrane.org/chapter_9/9_3_4_repeated_observations_on_participants.htm.

[21] Nikolakopoulou A., Higgins J.P.T., Papakonstantinou T., Chaimani A., Del Giovane C., Egger M., Salanti G. (2020). CINeMA: An Approach for Assessing Confidence in the Results of a Network Meta-Analysis. PLoS Med..

[22] Ho E.K.-Y., Chen L., Simic M., Ashton-James C.E., Comachio J., Wang D.X.M., Hayden J.A., Ferreira M.L., Ferreira P.H. (2022). Psychological Interventions for Chronic, Non-Specific Low Back Pain: Systematic Review with Network Meta-Analysis. BMJ.

[23] Concoff A., Rosen J., Fu F., Bhandari M., Boyer K., Karlsson J., Einhorn T.A., Schemitsch E. (2019). A Comparison of Treatment Effects for Nonsurgical Therapies and the Minimum Clinically Important Difference in Knee Osteoarthritis. JBJS Rev..

[24] Rücker G., Schwarzer G. (2015). Ranking Treatments in Frequentist Network Meta-Analysis Works without Resampling Methods. BMC Med. Res. Methodol..

[25] Chaimani A., Salanti G. (2015). Visualizing Assumptions and Results in Network Meta-Analysis: The Network Graphs Package. Stata J..

[26] Veroniki A.A., Vasiliadis H.S., Higgins J.P.T., Salanti G. (2013). Evaluation of Inconsistency in Networks of Interventions. Int. J. Epidemiol..

[27] Dias S., Welton N.J., Caldwell D.M., Ades A.E. (2010). Checking Consistency in Mixed Treatment Comparison Meta-Analysis. Stat. Med..

[28] White I.R., Barrett J.K., Jackson D., Higgins J.P.T. (2012). Consistency and Inconsistency in Network Meta-Analysis: Model Estimation Using Multivariate Meta-Regression. Res. Synth Methods.

[29] Kongtharvonskul J., Woratanarat P., McEvoy M., Attia J., Wongsak S., Kawinwonggowit V., Thakkinstian A. (2016). Efficacy of Glucosamine plus Diacerein versus Monotherapy of Glucosamine: A Double-Blind, Parallel Randomized Clinical Trial. Arthritis Res. Ther..

[30] Kumar V., Sareen S., Bhatia S. (2020). Oxaceprol Monotherapy versus Oxaceprol and Glucosamine Combination Therapy for Knee Osteoarthritis. Int. J. Med. Dent. Sci..

[31] Sun Y., Wang C., Gong C. (2020). Repairing Effects of Glucosamine Sulfate in Combination with Etoricoxib on Articular Cartilages of Patients with Knee Osteoarthritis. J. Orthop. Surg. Res..

[32] Zhijun L., Rongchun C., Feixiang L., Yaohong W., Ning L., Shufang Z., Mingliang Z., Hongfa Z. (2019). Therapeutic Effects of Combined Meloxicam and Glucosamine Sulfate Treatment on Patients with Osteoarthritis, and Its Effect on Serum CTX-I, CTX-II, COMP and MMP-3. Trop. J. Pharm. Res..

[33] Arti H.R., Azemi M.E. (2012). Comparing the Effect of Glucosamine and Glucosamine with Alendronate in Symptomatic Relieve of Degenerative Knee Joint Disease: A Double- Blind Randomized Clinical Trial Study. Jundishapur. J. Nat. Pharm. Prod..

[34] Leffler C.T., Philippi A.F., Leffler S.G., Mosure J.C., Kim P.D. (1999). Glucosamine, Chondroitin, and Manganese Ascorbate for Degenerative Joint Disease of the Knee or Low Back: A Randomized, Double-Blind, Placebo-Controlled Pilot Study. Mil. Med..

[35] Riley R.D., Jackson D., Salanti G., Burke D.L., Price M., Kirkham J., White I.R. (2017). Multivariate and Network Meta-Analysis of Multiple Outcomes and Multiple Treatments: Rationale, Concepts, and Examples. BMJ.

[36] Simental-Mendía M., Sánchez-García A., Vilchez-Cavazos F., Acosta-Olivo C.A., Peña-Martínez V.M., Simental-Mendía L.E. (2018). Effect of glucosamine and chondroitin sulfate in symptomatic knee osteoarthritis: A systematic review and meta-analysis of randomized placebo-controlled trials. Rheumatol. Int..

[37] Zhu X., Sang L., Wu D., Rong J., Jiang L. (2018). Effectiveness and Safety of Glucosamine and Chondroitin for the Treatment of Osteoarthritis: A Meta-Analysis of Randomized Controlled Trials. J. Orthop. Surg. Res..

[38] Meng Z., Liu J., Zhou N. (2022). Efficacy and Safety of the Combination of Glucosamine and Chondroitin for Knee Osteoarthritis: A Systematic Review and Meta-Analysis. Arch. Orthop. Trauma Surg..

[39] Wandel S., Juni P., Tendal B., Nuesch E., Villiger P.M., Welton N.J., Reichenbach S., Trelle S. (2010). Effects of Glucosamine, Chondroitin, or Placebo in Patients with Osteoarthritis of Hip or Knee: Network Meta-Analysis. Br. Med. J..

[40] Zhu X., Wu D., Sang L., Wang Y., Shen Y., Zhuang X., Chu M., Jiang L. (2018). Comparative Effectiveness of Glucosamine, Chondroitin, Acetaminophen or Celecoxib for the Treatment of Knee and/or Hip Osteoarthritis: A Network Meta-Analysis. Clin. Exp. Rheumatol..

[41] Zeng C., Wei J., Li H., Wang Y., Xie D., Yang T., Gao S., Li Y., Luo W., Lei G. (2015). Effectiveness and Safety of Glucosamine, Chondroitin, the Two in Combination, or Celecoxib in the Treatment of Osteoarthritis of the Knee. Sci. Rep..

[42] Gruenwald J., Petzold E., Busch R., Petzold H.-P., Graubaum H.-J. (2009). Effect of Glucosamine Sulfate with or without Omega-3 Fatty Acids in Patients with Osteoarthritis. Adv. Ther..

[43] McAlindon T.E., Bannuru R.R., Sullivan M.C., Arden N.K., Berenbaum F., Bierma-Zeinstra S.M., Hawker G.A., Henrotin Y., Hunter D.J., Kawaguchi H. (2014). OARSI Guidelines for the Non-Surgical Management of Knee Osteoarthritis. Osteoarthr. Cartil..

[44] Cooper C., Chapurlat R., Al-Daghri N., Herrero-Beaumont G., Bruyère O., Rannou F., Roth R., Uebelhart D., Reginster J.-Y. (2019). Safety of Oral Non-Selective Non-Steroidal Anti-Inflammatory Drugs in Osteoarthritis: What Does the Literature Say?. Drugs Aging.

[45] Puljak L., Marin A., Vrdoljak D., Markotic F., Utrobicic A., Tugwell P. (2017). Celecoxib for Osteoarthritis. Cochrane Database Syst. Rev..

[46] Selvan T., Rajiah K., Nainar M.S.-M., Mathew E.M. (2012). A Clinical Study on Glucosamine Sulfate versus Combination of Glucosamine Sulfate and NSAIDs in Mild to Moderate Knee Osteoarthritis. Sci. World J..

[47] Kivitz A.J., Moskowitz R.W., Woods E., Hubbard R.C., Verburg K.M., Lefkowith J.B., Geis G.S. (2001). Comparative Efficacy and Safety of Celecoxib and Naproxen in the Treatment of Osteoarthritis of the Hip. J. Int. Med. Res..

[48] Feng X., Beiping L. (2017). Therapeutic Efficacy of Ozone Injection into the Knee for the Osteoarthritis Patient along with Oral Celecoxib and Glucosamine. J. Clin. Diagn. Res..

[49] Gang D., Xiaguang C., Kanghua Y., Aiping W., Guangxuan Z. (2019). Combined Effect of Celecoxib and Glucosamine Sulfate on Inflammatory Factors and Oxidative Stress Indicators in Patients with Knee Osteoarthritis. Trop. J. Pharm. Res..

[50] Sánchez-Romero E.A., Battaglino A., Campanella W., Turroni S., Bishop M.D., Villafañe J.H. (2021). Impact on Blood Tests of Lower Limb Joint Replacement for the Treatment of Osteoarthritis: Hip and Knee. Top. Geriatr. Rehabil..

[51] Conrozier T., Lohse T. (2022). Glucosamine as a Treatment for Osteoarthritis: What If It’s True?. Front. Pharmacol..

[52] Henrotin Y., Chevalier X., Herrero-Beaumont G., McAlindon T., Mobasheri A., Pavelka K., Schön C., Weinans H., Biesalski H. (2013). Physiological Effects of Oral Glucosamine on Joint Health: Current Status and Consensus on Future Research Priorities. BMC Res. Notes.

[53] Hossain F.M., Hong Y., Jin Y., Choi J., Hong Y. (2019). Physiological and Pathological Role of Circadian Hormones in Osteoarthritis: Dose-Dependent or Time-Dependent?. J. Clin. Med..

[54] Sumsuzzman D.M., Choi J., Khan Z.A., Kamenos G., Hong Y. (2021). Melatonin Maintains Anabolic-Catabolic Equilibrium and Regulates Circadian Rhythm During Osteoarthritis Development in Animal Models: A Systematic Review and Meta-Analysis. Front. Pharmacol..

[55] Li Z., Fu B., Wei A., Wu Y., Huang M., Zhang E., Cui B., Wang B., Peng H. (2023). D-Glucosamine Induces Circadian Phase Delay by Promoting BMAL1 Degradation through AMPK/mTOR Pathway. Life Sci..

[56] Coulson S., Butt H., Vecchio P., Gramotnev H., Vitetta L. (2013). Green-Lipped Mussel Extract (*Perna canaliculus*) and Glucosamine Sulphate in Patients with Knee Osteoarthritis: Therapeutic Efficacy and Effects on Gastrointestinal Microbiota Profiles. Inflammopharmacol.

[57] Derry S., Conaghan P., Da Silva J.A.P., Wiffen P.J., Moore R.A. (2016). Topical NSAIDs for Chronic Musculoskeletal Pain in Adults. Cochrane Database Syst. Rev..

[58] Sumsuzzman D.M., Kim Y., Baek S., Hong Y. (2024). Cutting-Edge Methodological Guidance for Authors in Conducting the Systematic Review and Meta-Analysis. J. Lifestyle Med..

