# Supplementary material for: Comparative Efficacy of Glucosamine-Based Combination Therapies in Alleviating Knee Osteoarthritis Pain: A Systematic Review and Network Meta-Analysis
Source: J Clin Med. 2024 Dec 6;13(23):7444. doi: 10.3390/jcm13237444 (PMC11641979; doi:10.3390/jcm13237444)
Supplement: Supplementary file 1 [file jcm-13-07444-s001.zip › jcm-3308263-supplementary.pdf]

SUPPLEMENTARY FILE

Table S1. Search strategies. 3
Table S2. Exclusion reasons after full-text screening. 6
Table S3. Characteristics of included studies in network meta-analysis. 9
Table S4. Results of a side-splitting method for overall pain. 12
Table S5. Results of a side-splitting method for short-term pain. 14
Table S6. Results of a side-splitting method for long-term pain. 16
Table S7. Results of global inconsistency tests for the primary analyses. 16
Table S8. Sensitivity analysis of the side-splitting method for statistical inconsistency in overall pain. 17
Table S9. Sensitivity analysis of the side-splitting method for statistical inconsistency in short-term pain. 19
Table S10. Sensitivity analysis of global inconsistency tests for the primary analyses. 20
Table S11. Confidence rating for overall pain using CINeMA. 22
Table S12. Pairwise and network meta-analysis results for overall pain. 29
Table S13. SUCRA and mean rank results for overall pain. 32
Table S14. Studies excluded from the network meta-analysis for overall pain. 32
Table S15. Confidence rating for short-term pain using CINeMA. 33
Table S16. Pairwise and network meta-analysis results for short-term pain. 40
Table S17. SUCRA and mean rank results for short-term pain. 43
Table S18. Studies excluded from the network meta-analysis for short-term pain. 43
Table S19. Confidence rating for long-term pain using CINeMA. 44
Table S20. Pairwise and network meta-analysis results for long-term pain. 45
Table S21. SUCRA and mean rank results for long-term pain. 46
Table S22. Studies excluded from the network meta-analysis for long-term pain. 46
Table S23. Pairwise and network meta-analysis results for adverse events. 47
Table S24. Rank results for adverse events. 49
Table S25. Results of a side-splitting method for adverse events. 50
Table S26. Results of global inconsistency tests for the primary analyses. 51
Table S27. The quality assessment included studies in the network meta-analysis. 51
Figure S1. Risk of bias graph. 52
Figure S2. Risk of bias summary. 53

Figure S3. Assessment of transitivity in terms of mean age distribution in the network of interventions for overall pain outcome. 54

Figure S4. Assessment of transitivity in terms of percentage of females in the network of interventions for overall pain outcome. 54

Figure S5. Assessment of transitivity in terms of mean age distribution in the network of interventions for short-term pain outcome. 55

Figure S6. Assessment of transitivity in terms of percentage of females in the network of interventions for short-term pain outcome. 55

Figure S7. Assessment of transitivity in terms of mean age distribution in the network of interventions for long-term pain outcome. 56

Figure S8. Assessment of transitivity in terms of percentage of females in the network of interventions for long-term pain outcome. 56

Figure S9. Inconsistency factors for overall pain of all trials. 57

Figure S10. Inconsistency factors for short-term pain of 20 trials. 58

Figure S11. Inconsistency factors for long-term pain of 9 trials. 58

Figure S12. Sensitivity analysis to resolve the inconsistency for overall pain. 59

Figure S13. Sensitivity analysis to resolve the inconsistency for short-term pain. 60

Figure S14. Network plot for adverse events. 61

Figure S15. Interval plot for adverse events. 62

Figure S16. SUCRA ranking for adverse events. 63

Figure S17. Assessment of transitivity in terms of mean age distribution in the network of interventions for adverse events outcome. 64

Figure S18. Assessment of transitivity in terms of percentage of females in the network of interventions for adverse events outcome. 64

Figure S19. Inconsistency factors for overall pain of all trials. 65

Figure S20. Comparison adjusted funnel plot for overall pain. 66

Figure S21. Comparison adjusted funnel plot for short-term pain. 67

Figure S22. Comparison adjusted funnel plot for long-term pain. 68

Figure S23. Comparison adjusted funnel plot for adverse events. 69

Figure S24. Sensitivity analysis of network meta-analysis for overall pain and short-term pain. 70

Table S1. Search strategies.

Database: Ovid MEDLINE(R) and Epub Ahead of Print, In-Process, In-Data-Review & Other Non-Indexed Citations, Daily and Versions <1946 to November 30, 2023>

1	exp Glucosamine/	15404
2	Glucosamin*.ti,ab.	21950
3	"Glucosamine Sulfate".ti,ab.	359
4	"Glucosamine hydrochloride".ti,ab.	312
5	Chitosamine.ti,ab.	4
6	"2-Amino-2-Deoxyglucose".ti,ab.	19
7	Dona.ti,ab.	164
8	1 or 2 or 3 or 4 or 5 or 6 or 7	31325
9	exp Osteoarthritis/	79752
10	Osteoarthr$.ti,ab.	95671
11	(degenerative adj2 arthritis).ti,ab.	1587
12	Arthrosis.ti,ab.	5735
13	9 or 10 or 11 or 12	123385
14	8 and 13	1315
15	randomized controlled trial.pt.	609373
16	controlled clinical trial.pt.	95566
17	randomized.ab.	636083
18	placebo.ab.	246044
19	clinical trials as topic.sh.	201801
20	randomly.ab.	427801
21	trial.ti.	303631
22	15 or 16 or 17 or 18 or 19 or 20 or 21	1582036
23	14 and 22	404

Database: Embase <1974 to November 30, 2023>

1 exp Glucosamine/ 20367

2 Glucosamin*.ti,ab. 24211

3 "Glucosamine Sulfate".ti,ab. 599

4 "Glucosamine hydrochloride".ti,ab. 461

5 Chitosamine.ti,ab. 4

6 "2-Amino-2-Deoxyglucose".ti,ab. 14

7 Dona.ti,ab. 285

8 1 or 2 or 3 or 4 or 5 or 6 or 7 36401

9 exp Osteoarthritis/ 165121

10 Osteoarthr$.ti,ab. 133944

11 (degenerative adj2 arthritis).ti,ab. 1937

12 Arthrosis.ti,ab. 6755

13 9 or 10 or 11 or 12 196753

14 8 and 13 3250

15 (random$ or placebo$ or single blind$ or double blind$ or triple blind$).ti,ab. 2183426

16 RETRACTED ARTICLE/ 14942

17 15 or 16 2197811

18 (animal$ not human$).sh,hw. 4922890

19 (book or conference paper or editorial or letter or review).pt. not exp randomized controlled trial/ 6016802

20 (random sampl$ or random digit$ or random effect$ or random survey or random regression).ti,ab. not exp randomized controlled trial/ 168379

21 18 or 19 or 20 10847035

22 17 not 21 1671680

23 14 and 22 540

Database: Cochrane Library (November 30, 2023)

#	Terms	Number of Citations
**1**	MeSH descriptor: [Glucosamine] explode all trees	314
**2**	(Glucosamin*):ti,ab,kw	1249
**3**	("Glucosamine Sulfate"):ti,ab,kw	274
**4**	(dona):ti,ab,kw	81
**5**	(Glucosamine hydrochloride):ti,ab,kw	130
**6**	#1 or #2 or #3 or #4 or #5	1331
**7**	MeSH descriptor: [Osteoarthritis] explode all trees	10744
**8**	(osteoarthr$):ti,ab,kw	23771
**9**	((degenerative near/2 arthritis)):ti,ab,kw	132
**10**	(arthrosis):ti,ab,kw	719
**11**	#7 or #8 or #9 or #10	24071
**12**	#6 and # 11	274

Database: Clinical Trials Registry Platform (November 30, 2023)

1. Search string for ClinicalTrials.gov advanced interface (n = 52)

Osteoarthritis in Condition AND

Glucosamine OR dona in Intervention

2. Search string for WHO-ICTRP advanced interface (n = 12)

Osteoarthritis in Condition AND

Glucosamine OR dona in Intervention

Table S2. Exclusion reasons after full-text screening.

Sl/No.	Title	Reason
1	Efficacy and safety of undenatured type II collagen supplement in modulating knee joint function in osteoarthritic subjects	Abstracts and posters for congresses
2	Comparison of Glucosamine-Chondroitin Sulfate with and without Methylsulfonylmethane in Grade I-II Knee Osteoarthritis: a Double Blind Randomized Controlled Trial	
3	Maintenance therapy with glucosamine sulfate and physiotherapy on osteoarthritis progression	
4	Non-inferiority clinical trial on the efficacy and safety of chondroitin sulfate and glucosamine hydrochloride in combination vs. Celecoxib in patients with knee osteoarthritis	
5	Multicentric osteoarthritis intervention study with sysadoa (MOVES): effects of combined glucosamine hydrochloride and chondroitin sulfate vs celecoxib for painful knee osteoarthritis	
6	Randomized, double-blind, multicenter, non inferiority clinical trial with combined glucosamine and chondroitin sulfate vs celecoxib for painful knee osteoarthritis	
7	Medicinal methods of knee osteoarthritis treatment and indices of T2-mapping of the cartilage	
8	Combined chondroitin sulfate and glucosamine versus celecoxib for painful knee osteoarthritis: Post-hoc analyses by kellgren and lawrence grade and C-reactive protein level from a randomized, double-blind, multicentre clinical trial	
9	Combined chondroitin sulfate and glucosamine is comparable to celecoxib for painful knee osteoarthritis. Results from a multicenter, randomized, double-blind, phase IV non-inferiority trial	
10	The effect of different treatments on the clinical course of knee osteoarthritis	
11	Non-inferiority clinical trial on the efficacy and safety of chondroitin sulfate and glucosamine hydrochloride in combination vs. celecoxib in patients with knee osteoarthritis	
12	Chondroitin sulfate plus glucosamine sulfate does not show superiority over placebo in a randomised, double blind, placebo-controlled clinical trial in patients with knee osteoarthritis	
13	Combined chondroitin sulfate and Glucosamine versus celecoxib for painful Knee osteoarthritis: post-hoc analyses by Kellgren and Lawrence grade and C-reactive protein level from a randomized, double-blind, multicentre clinical trial	
14	The natural mineral supplement, Aquamin, provides relief from the symptoms of knee osteoarthritis symptoms: A randomised controlled pilot trial	
15	The efficacy of a standardized rose-hip powder containing seeds and shells compared with glucosamine sulfate in patients with osteoarthritis of the knee - A blinded, parallel, randomized study	
16	The effect of glucosamine and chondroitin sulfate on MRI-based osteoarthritis features in the patellofemoral joint in people with knee osteoarthritis: a randomised placebo-controlled trial of single and combination regimens	
17	Combination treatment with glucosamine-chondroitin sulfate reduces pain, disability and nsaid consumption in patients with chronic low back pain: Final results from a large, community-based, pilot, open prospective interventional study	
18	Severe chronic low back pain: Combination treatment with glucosamine-chondroitin sulfate reduces pain, disability and nsaid consumption-results from a large, community-based, pilot, open prospective interventional study	
19	Combined glucosamine and chondroitin sulfate, once of three times daily, provide clinically relevant analgesia in knee osteoarthritis	

Sl/No.	Title	Reason
20	Multicentric osteoarthritis intervention study with sysadoa (moves): effects of combined glucosamine hydrochloride and chondroitin sulfate vs. Celecoxib for painful knee osteoarthritis	
21	Effect of diacerin vs glucosaminechondroitin on disease progression, and measures of function in persons with knee osteoarthritis: a 2-year randomised, doubleblind, placebo-controlled trial	
22	Effectiveness of the treatment with nimesulide, glucosamine sulfate+ chondroitin sulfate, intraarticular traumeel and platelet-rich plasma in patients with knee osteoarthritis	
23	Combined chondroitin sulfate and glucosamine is more efficient than celebrex in reducing serum levels of COLL2-1, a cartilage degradation biomarker, in patients with severe OA: results from a randomized, double-blind, multicentric clinical trial	
24	Effects of glucosamine-chondroitin sulfate, glucosamine-chondroitin sulfate-methylsulfonylmethane, or placebo in patients with first and second grade of knee osteoarthritis: a double blind randomized controlled study	
25	Differences in serum protein biomarkers between combined glucosamine and chondroitin versus celecoxib treatment in a randomized, double-blind trial in osteoarthritis patients	
26	The effect of the use of herbal supply on daily life activities containing OTC (Chondroitin sulfate + glucosamine) in patients with knee osteoarthritis	Irrelevant intervention
27	Glucosamine and chondroitin sulphate supplementation along with diet therapy provides better symptomatic relief in osteoarthritic patients as compared to diet therapy alone	
28	The effect of the dietary supplement containing both glucosamine and chondroitin sulfate on gait of healthy volunteer : a randomized, placebo-controlled, double-blind, clinical study-stratified analysis based on jkom and body Weight	
29	Effect of a dietary supplement containing glucosamine hydrochloride, chondroitin sulfate and quercetin glycosides on knee joint functions - Stratified analysis based on kellgren-lawrence grades	
30	A Double-Blind, Randomized Controlled 12-Week Follow-Up Trial to Evaluate the Efficacy and Safety of Polycan in Combination with Glucosamine for the Treatment of Knee Osteoarthritis	
31	Effect of a glucosamine-based combination supplement containing chondroitin sulfate and antioxidant micronutrients in subjects with symptomatic knee osteoarthritis: A pilot study	
32	A natural mineral supplement provides relief from knee osteoarthritis symptoms: a randomized controlled pilot trial	
33	Effects of diet type and supplementation of glucosamine, chondroitin, and MSM on body composition, functional status, and markers of health in women with knee osteoarthritis initiating a resistance-based exercise and weight loss program	
34	Acute effects of combination of glucosamine sulphate lontophoresis with exercise on fasting plasma glucose of participants with knee osteoarthritis	
35	Effects of glucosamine sulfate and exercise therapy on serum leptin levels in patients with knee osteoarthritis: preliminary results of randomized controlled clinical trial	
36	lucosamine/chondroitin combined with exercise for the treatment of knee osteoarthritis: a preliminary study	
37	Efficacy of pulsed Nd: YAG laser in the treatment of patients with knee osteoarthritis: a randomized controlled trial	
38	The efficacy of topical glucosamine sulfate-chondroitin sulfate in knee osteoarthritis treated with physical therapy: a randomized, double-blind, placebo-controlled study	
39	Assessment of the effect of glucosamine sulfate and exercise on knee cartilage using magnetic resonance imaging in patients with knee osteoarthritis: a randomized controlled clinical trial	

Sl/No.	Title	Reason
40	Glucosamine, chondroitin sulfate combined with bone health exercise can promote bone mineral density and muscle strength in postmenopausal women: a random community trial	
41	The efficacy and safety of a combination of glucosamine hydrochloride, chondroitin sulfate and bio-curcumin with exercise in the treatment of knee osteoarthritis: a randomized, double-blind, placebo-controlled study	
42	The synergic use of the high power laser therapy and glu-cosamine sulfate in knee osteoarthritis: A randomized controlled trial	
43	Palm vitamin E and glucosamine sulphate in the treatment of osteoarthritis of the knee	
44	Additive effects of glucosamine or risedronate for the treatment of osteoarthritis of the knee combined with home exercise: a prospective randomized 18-month trial	
45	Efficacy and tolerance of enzymatic hydrolysed collagen (EHC) vs. glucosamine sulphate (GS) in the treatment of knee osteoarthritis (KOA)	
46	Ayurvedic medicine offers a good alternative to glucosamine and celecoxib in the treatment of symptomatic knee osteoarthritis: a randomized, double-blind, controlled equivalence drug trial	
47	Symptoms-modifying effects of electromotive administration of glucosamine sulphate among patients with knee osteoarthritis	
48	Clinical study comparing a new signle dose chewable tablet as treatment knee osteoarthritis with moderate to severe pain, compared to Placebo and Celecoxib	
49	A randomized, double blind, placebo controlled trial of a topical cream containing glucosamine sulfate, chondroitin sulfate, and camphor for osteoarthritis of the knee	
50	Treatment of knee osteoarthritis with a new formulation of a fixed-dose combination of glucosamine sulfate and bovine chondroitin: a multicenter, randomized, single-blind, non-inferiority clinical trial	Irrelevant comparator
51	Combined glucosamine and chondroitin sulfate, once or three times daily, provides clinically relevant analgesia in knee osteoarthritis	
52	Clinical and biochemical study of the comparative efficacy of topical versus oral glucosamine/chondroitin sulfate on osteoarthritis of the knee	
53	Multicenter, randomized, double-blind clinical trial to evaluate efficacy and safety of combined glucosamine sulfate and chondroitin sulfate capsules for treating knee osteoarthritis	
54	Acujoint™, a highly efficient formulation with natural bioactive compounds,exerts potent anti-arthritis effects in human osteoarthritis – A pilot randomized double blind clinical study compared to combination of glucosamine and chondroitin	
55	N-acetyl glucosamine and proteoglycan containing supplement improves the locomotor functions of subjects with knee pain	Irrelevant population
56	Glucosamine-containing supplement improves locomotor functions in subjects with knee pain: a randomized, double-blind, placebo-controlled study	
57	Differences in Serum Biomarkers Between Combined Glucosamine and Chondroitin Versus Celecoxib in a Randomized, Double-blind Trial in Osteoarthritis Patients	Irrelevant outcome
58	Combined medication ARTRA in the treatment of osteoarthrosis	Foreign language
59	Efficacy of dietary supplements in patients with osteoarthritis: the doubt persists	
60	Efficacy of Oral Glucosamine Sulphate and Sulfasalazine combination in the treatment of Osteoarthritis	Inaccessible

Table S3. Characteristics of included studies in network meta-analysis.

Author, Year (Country)	Design	Center (M/S)	Fund	Age (Y)	% female	Disease severity	Duration of disease (Y)	BMI	Treatment arm	Dose (mg/day)	n	Follow-up	Outcomes
Clegg, 2006 (USA) [1]	Parallel	M	Industry	58.6	64.1	Mild-moderate	5.54	31.74	G+CS	1500+1200	317	24 weeks	WOMAC pain, AEs
									Placebo	-	313		
									G	1500	317		
									CS	1200	318		
									Celecoxib	200	318		
Crowley, 2009 (Canada) [2]	Parallel	M	Non-industry	58.8	57.5	Moderate	NR	30.55	G+CS	1500+1200	26	90 days	VAS, AEs
									UC-II	10	26		
Das, 2000 (USA) [3]	Parallel	S	Non-industry	65.25	75	Mild-moderate	6.5	30.35	G+CS+MA	1000+800+152	33	2, 6 months	Lequesne ISK, AEs
									Placebo	-	39		
Feng, 2017 (China) [4]	Parallel	S	Non-industry	64.57	56.5	Mild-moderate	4.83	NR	G+O+Celecoxib	720+20*+200	35	6 weeks	VAS
									G+Celecoxib	720+200	41		
Fransen, 2015 (Australia) [5]	Parallel	S	Non-industry	60.5	56	Mild	NR	28.97	G+CS	1500+800	151	2 years	WOMAC pain, AEs
									Placebo	-	151		
									G	1500	152		
									CS	800	151		
Gang, 2019 (China) [6]	Parallel	S	Non-industry	64.85	66.79	Mild-moderate	5.4	NR	G+Celecoxib	1500+200	60	8 weeks	VAS, AEs
									Celecoxib	200	60		
Gruenwald, 2009 (Germany) [7]	Parallel	M	Industry	62.3	63.48	Moderate-severe	NR	29.4	G+omega-3	1500+1932	90	26 weeks	WOMAC pain, AEs
									G	1500	87		
Hochberg, 2016 (France, Germany,	Parallel	M	Industry	62.7	83.85	Mild-moderate	NR	31	G+CS	1500+1200	264	60, 180 days	WOMAC pain, AEs
									Celecoxib	200	258		

Author, Year (Country)	Design	Center (M/S)	Fund	Age (Y)	% female	Disease severity	Duration of disease (Y)	BMI	Treatment arm	Dose (mg/day)	n	Follow-up	Outcomes
Poland, Spain) [8]													
Kalman, 2017 (USA) [9]	Parallel	S	Industry	57.25	73	Mild	NR	29.05	G+CS	375+300	45	12 weeks	WOMAC pain, AEs
									Placebo	-	45		
Khanna, 2020 (India) [10]	Parallel	S	Industry	52.45	52.2	Mild-moderate	NR	25.09	G+CGM	500+400	40	84 days	WOMAC pain
									G+CS	500+415	40		
Lubis, 2017 (Indonesia) [11]	Parallel	S	Non-industry	60.66	61.66	Mild	NR	25.13	G+CS+MSM	1500+1200+500	48	12 weeks	VAS
									G+CS	1500+1200	49		
									Placebo	-	50		
Lugo, 2016 (India) [12]	Parallel	M	Industry	53	52.08	Mild-moderate	NR	NR	G+CS	1500+1200	57	90, 180 days	WOMAC pain, AEs
									UC-II	40	54		
									Placebo	-	53		
Madhu, 2013 (India) [13]	Parallel	S	Industry	57.09	69.16	Moderate	NR	27.66	G+CGM	750+500	30	42 days	VAS, AEs
									CGM	500	30		
									G	750/day	30		
									Placebo	NA	30		
Mirunalini, 2014 (India) [14]	Parallel	S	Non-industry	50.99	66.66	Mild-moderate	2.84	27.7	G+CS	1500+1200	30	12, 24 weeks	VAS, AEs
									Diacerein	100	30		
Puente, 2017 (Cuba) [15]	Parallel	S	Non-industry	68	88.3	Mild-moderate	NR	24.4	G+CS	375+300	30	12 weeks	WOMAC pain, AEs
									D-002	50	30		
Roman-Blas, 2017 (Spain) [16]	Parallel	S	Non-industry	66	83.54	Mild-moderate	6.2	28.2	G+CS	1500+1200	80	6 months	WOMAC pain, AEs
									Placebo	-	78		
Selvan, 2012 (India) [17]	Parallel	S	Non-industry	48.45	60.46	Mild-moderate	NR	25.71	G+Ibuprofen	1500+NR	43	12 weeks	VAS
									G	1500	39		
		S			75.05		5.55		G+Ibuprofen	1500+1200	30		

Author, Year (Country)	Design	Center (M/S)	Fund	Age (Y)	% female	Disease severity	Duration of disease (Y)	BMI	Treatment arm	Dose (mg/day)	n	Follow-up	Outcomes
Shahine, 2014 (Egypt) [18]	Parallel		Non-industry	52.15		Mild-moderate		35.45	Ibuprofen	1200	30	12 weeks	WOMAC pain
Thomas, 2020 (India) [19]	Parallel	S	Industry	52	51.35	Mild-moderate	3.44	24.88	G+CS	1000+830	37	42 days	WOMAC pain, AEs
									CGM	400	35		
Tsuji, 2016 (Japan) [20]	Parallel	S	Industry	66.85	78	NR	2 to 5	24.35	G+CS	100+180	25	12, 24 weeks	VAS, AEs
									Placebo	-	25		
Usha, 2004 (India) [21]	Parallel	S	Industry	51.25	64.28	Mild-moderate	3.04	25.7	G+MSM	1500+1500	30	12 weeks	VAS
									G	1500	30		
									MSM	1500	30		
									Placebo	-	28		
Wang, 2021 (China) [22]	Parallel	S	Non-industry	61.15	74.45	NR	NR	25.43	G+CS+HA	750+250+50-	24	8 weeks	WOMAC pain
									Placebo	-	23		
Wang, 2021_0 (China) [23]	Parallel	S	Non-industry	59.6	74.9	Mild	NR	25.2	G+CS+HA	750+250+50	47	8 weeks	WOMAC pain, AEs
									Placebo	-	47		
Zhang, 2021 (China) [24]	Parallel	S	Non-industry	55.15	45.29	Mild	NR	NR	G+Celecoxib	960+200	68	12 weeks	VAS, AEs
									Celecoxib	200	60		

*parenteral dosage form

M, multiple; S, single; Y, year; BMI, body mass index; n, number of patients; G+CS; glucosamine+chondroitin sulfate; WOMAC, Western Ontario and McMaster Universities Osteoarthritis Index; AEs, adverse events; G, glucosamine; CS, chondroitin sulfate; NR, not reported; VAS, visual analogue scale; UC-II, undenatured type II collagen; G+CS+MA, glucosamine+chondroitin sulfate+manganese ascorbate; G+O+Celecoxib, glucosamine+ozone+celecoxib; G+Celecoxib, glucosamine+celecoxib; G+omega-3, glucosamine+omega-3; G+CGM, glucosamine+Curcumin derivatives; G+CS+MSM, glucosamine+chondroitin sulfate+methylsulfonylmethane; D-002, inhibitor of both cyclooxygenase and 5-lipoxygenase activities; G+Ibuprofen, glucosamine+ibuprofen; G+MSM, glucosamine+methylsulfonylmethane; MSM, methylsulfonylmethane; G+CS+HA, glucosamine+chondroitin sulfate+hyaluronic acid; G+Celecoxib, glucosamine+celecoxib

Table S4. Results of a side-splitting method for overall pain.

Side	Direct		Indirect		Difference		*p*	tau
	Coef.	Std. Err.	Coef.	Std. Err.	Coef.	Std. Err.		
Placebo UC-II	-0.72432	0.577544	-0.45016	0.586113	-0.27416	0.822742	0.739	0.5427
CGM Placebo	1.176781	0.601017	0.718844	0.558489	0.457937	0.820671	0.577	0.537138
CGM G	0.433173	0.596443	0.936293	0.623923	-0.50312	0.862334	0.56	0.5369
CGM G+CGM	0.738274	0.534112	2.663516	0.750328	-1.92524	0.921014	0.037	0.464965
CGM G+CS	1.150501	0.591327	0.571634	0.533665	0.578868	0.796534	0.467	0.53313
CS Placebo	0.024029	0.39017	0.412173	0.706876	-0.38814	0.807419	0.631	0.542873
CS Celecoxib	-0.14454	0.552776	0.071721	0.721924	-0.21627	0.909249	0.812	0.547052
CS G	0.002623	0.375287	-1.06141	0.944735	1.064034	1.016537	0.295	0.521509
CS G+CS	-0.02219	0.392846	0.138032	0.714453	-0.16022	0.815339	0.844	0.546734
Celecoxib Placebo	0.138235	0.553973	0.215126	0.514234	-0.07689	0.755879	0.919	0.548216
Celecoxib G	0.122862	0.5472	-0.3171	0.595095	0.439966	0.808436	0.586	0.54141
Celecoxib G+CS	0.014321	0.389066	0.398211	0.859394	-0.38389	0.943365	0.684	0.543843
Celecoxib G+Celecoxib	-2.4797	0.41243	0.352135	25.82874	-2.83183	25.83205	0.913	0.523792
D-002 G+CS	0.52699	0.586096	-0.19807	63.25472	0.725057	63.25744	0.991	0.523764
Diacerein G+CS	-2.89256	0.645528	-0.19794	63.2543	-2.69462	63.25759	0.966	0.523763
G Placebo	0.360532	0.279703	-0.47596	0.741493	0.836494	0.792812	0.291	0.524391
G G+CGM	0.310165	0.586992	1.149955	0.613641	-0.83979	0.849414	0.323	0.526756
G G+CS	-0.02488	0.385295	0.360553	0.405417	-0.38543	0.5593	0.491	0.535897
G G+Ibuprofen	-1.73346	0.585394	-0.51671	28.28894	-1.21675	28.29502	0.966	0.523776
G G+MSM	-0.45538	0.582234	-1.81169	1.1743	1.356309	1.307529	0.3	0.520967
G G+omega-3	-0.2605	0.545098	0.517143	63.25482	-0.77764	63.25718	0.99	0.523765
G MSM	0.141326	0.581517	-1.21498	1.170313	1.356309	1.307529	0.3	0.520967
G+CGM Placebo	0.432704	0.515233	-1.23681	0.480676	1.669516	0.705479	**0.018**	0.444969
G+CGM G+CS	-1.51294	0.506254	0.214329	0.453911	-1.72727	0.679947	**0.011**	0.437132

Side	Direct Coef.	Direct Std. Err.	Indirect Coef.	Indirect Std. Err.	Difference Coef.	Difference Std. Err.	***p***	**tau**
G+CS Placebo	-0.04237	0.193221	0.91289	0.467325	-0.95526	0.505624	0.059	0.477318
G+CS G+CS+MSM	-0.48816	0.57829	0.136075	1.085709	-0.62424	1.229578	0.612	0.540685
G+CS UC-II	-0.44854	0.419897	-0.76734	1.090314	0.318802	1.167229	0.785	0.544251
G+CS+HA Placebo	-	-	-	-	-	-	-	-
G+CS+MA Placebo	-	-	-	-	-	-	-	-
G+CS+MSM Placebo	0.312925	0.57751	0.937162	1.086954	-0.62424	1.229578	0.612	0.540685
G+Celecoxib G+O+Celecoxib	-0.31455	0.572714	5.292003	63.26519	-5.60655	63.26772	0.929	0.52378
G+Ibuprofen Ibuprofen	2.103488	0.617287	3.97932	63.28	-1.87583	63.28301	0.976	0.523772
G+MSM Placebo	1.256228	0.589477	-0.10008	1.16341	1.356309	1.307529	0.3	0.520967
G+MSM MSM	-	-	-	-	-	-	-	-
MSM Placebo	0.65952	0.585142	-0.69679	1.164879	1.356309	1.307529	0.3	0.520967

For each comparison the direct and indirect estimates are provided along with the respective *p*-values of differences. *p*-values < 0.10 indicate significant disagreement between direct and indirect evidence (in bold). G+CS; glucosamine+chondroitin sulfate; G, glucosamine; CS, chondroitin sulfate; UC-II, undenatured type II collagen; G+CS+MA, glucosamine+chondroitin sulfate+manganese ascorbate; G+O+Celecoxib, glucosamine+ozone+celecoxib; G+Celecoxib, glucosamine+celecoxib; G+omega-3, glucosamine+omega-3; G+CGM, glucosamine+Curcumin derivatives; G+CS+MSM, glucosamine+chondroitin sulfate+methylsulfonylmethane; D-002, inhibitor of both cyclooxygenase and 5-lipoxygenase activities; G+Ibuprofen, glucosamine+ibuprofen; G+MSM, glucosamine+methylsulfonylmethane; MSM, methylsulfonylmethane; G+CS+HA, glucosamine+chondroitin sulfate+hyaluronic acid; G+Celecoxib, glucosamine+celecoxib.

Table S5. Results of a side-splitting method for short-term pain.

Side	Direct		Indirect		Difference		*p*	tau
	Coef.	Std. Err.	Coef.	Std. Err.	Coef.	Std. Err.		
Placebo UC-II	-0.56118	0.761412	-0.62695	0.828695	0.065764	1.12535	0.953	0.735781
CGM Placebo	1.17643	0.780827	0.966375	0.783369	0.210055	1.106156	0.849	0.732793
CGM G	0.434242	0.778901	0.573476	1.157962	-0.13923	1.395442	0.921	0.734302
CGM G+CGM	0.74265	0.675474	2.663322	0.951261	-1.92067	1.166688	0.1	0.622234
CGM G+CS	1.150501	0.765154	0.588564	0.74249	0.561938	1.066185	0.598	0.721125
Celecoxib G+CS	0.233482	0.693566	-1.83154	17.55117	2.06502	17.56487	0.906	0.68798
Celecoxib G+Celecoxib	-2.51143	0.519318	0.704736	25.84648	-3.21617	25.85171	0.901	0.68797
D-002 G+CS	0.52699	0.736432	-0.42039	63.25881	0.94738	63.2631	0.988	0.687861
Diacerein G+CS	-1.11054	0.742279	-0.42022	63.2626	-0.69032	63.26695	0.991	0.68786
G Placebo	0.772188	0.512987	-1.13546	1.601623	1.907646	1.682601	0.257	0.675152
G G+CGM	0.310928	0.680641	2.232053	1.012259	-1.92113	1.219961	0.115	0.629431
G G+Ibuprofen	-1.73346	0.735905	-0.11094	28.29724	-1.62252	28.30684	0.954	0.687903
G G+MSM	-0.45538	0.773042	-1.20992	1.950554	0.754534	2.096165	0.719	0.728014
G MSM	0.141326	0.772502	-0.61321	1.948156	0.754534	2.096166	0.719	0.728014
G+CGM Placebo	0.431551	0.683456	-1.07241	0.688022	1.50396	0.970301	0.121	0.632172
G+CGM G+CS	-1.51294	0.607367	0.400402	0.587352	-1.91334	0.844913	**0.024**	0.551078
G+CS Placebo	0.039299	0.358287	0.891341	0.715569	-0.85204	0.800185	0.287	0.680867
G+CS G+CS+MSM	-0.48816	0.748431	0.480172	1.498384	-0.96833	1.674509	0.563	0.719771
G+CS UC-II	-0.40028	0.54721	-0.22474	1.534522	-0.17554	1.628561	0.914	0.736283
G+CS+HA Placebo	-	-	-	-	-	-	-	-
G+CS+MA Placebo	-	-	-	-	-	-	-	-
G+CS+MSM Placebo	0.312925	0.747829	1.28126	1.499286	-0.96833	1.674509	0.563	0.719771
G+Celecoxib G+O+Celecoxib	-0.31455	0.725863	5.881527	63.28111	-6.19607	63.28521	0.922	0.68791
G+Ibuprofen Ibuprofen	2.103488	0.76151	4.654696	63.28304	-2.55121	63.28762	0.968	0.68789
G+MSM Placebo	1.256228	0.778512	0.501694	1.944017	0.754534	2.096165	0.719	0.728014

Side	Direct		Indirect		Difference		*p*	tau
	Coef.	Std. Err.	Coef.	Std. Err.	Coef.	Std. Err.		
G+MSM MSM	-	-	-	-	-	-	--	-
MSM Placebo	0.65952	0.77523 5	- 0.09501	1.94489 7	0.75453 4	2.09616 5	0.719	0.72801 4

For each comparison the direct and indirect estimates are provided along with the respective *p*-values of differences. *P*-values < 0.10 indicate significant disagreement between direct and indirect evidence (in bold). G+CS; glucosamine+chondroitin sulfate; G, glucosamine; CS, chondroitin sulfate; UC-II, undenatured type II collagen; G+CS+MA, glucosamine+chondroitin sulfate+manganese ascorbate; G+O+Celecoxib, glucosamine+ozone+celecoxib; G+Celecoxib, glucosamine+celecoxib; G+omega-3, glucosamine+omega-3; G+CGM, glucosamine+Curcumin derivatives; G+CS+MSM, glucosamine+chondroitin sulfate+methylsulfonylmethane; D-002, inhibitor of both cyclooxygenase and 5-lipoxygenase activities; G+Ibuprofen, glucosamine+ibuprofen; G+MSM, glucosamine+methylsulfonylmethane; MSM, methylsulfonylmethane; G+CS+HA, glucosamine+chondroitin sulfate+hyaluronic acid; G+Celecoxib, glucosamine+celecoxib.

Table S6. Results of a side-splitting method for long-term pain.

Side	Direct		Indirect		Difference		*p*	tau
	Coef.	Std. Err.	Coef.	Std. Err.	Coef.	Std. Err.		
Placebo UC-II	-0.72433	0.237826	-0.23022	0.445629	-0.49411	0.50435	0.327	0.132392
CS Placebo	0.016713	0.081879	-0.43214	0.300518	0.448849	0.307623	0.145	0.065311
CS Celecoxib	-0.14451	0.161693	0.057004	0.228407	-0.20152	0.279843	0.471	0.140885
CS G	-0.00054	0.110472	0.223094	63.25977	-0.22364	63.25986	0.997	0.122967
CS G+CS	-0.0328	0.123157	0.136381	0.288789	-0.16918	0.31367	0.59	0.144104
Celecoxib Placebo	0.138181	0.157063	-0.05303	0.181701	0.191207	0.240217	0.426	0.135363
Celecoxib G	0.12284	0.17022	-0.01422	0.238594	0.137056	0.293089	0.64	0.150569
Celecoxib G+CS	0.014852	0.0588	0.450141	0.179863	-0.43529	0.189255	**0.021**	3.18E-10
Diacerein G+CS	-2.89256	0.396863	0.021657	63.23651	-2.91421	63.23775	0.963	0.122968
G Placebo	0.019938	0.081281	-0.42889	0.297372	0.448828	0.307619	0.145	0.065312
G G+CS	-0.03289	0.122969	0.136276	0.288712	-0.16916	0.313663	0.59	0.144102
G G+omega_3	-0.2605	0.194739	-0.03687	63.38787	-0.22363	63.38818	0.997	0.122966
G+CS Placebo	-0.02493	0.096873	0.189801	0.453856	-0.21473	0.464161	0.644	0.152642
G+CS UC-II	-0.52451	0.233354	-1.01862	0.452668	0.494105	0.50435	0.327	0.132392
G+CS+MA Placebo	-	-	-	-	-	-	-	-

For each comparison the direct and indirect estimates are provided along with the respective *p*-values of differences. *P*-values < 0.10 indicate significant disagreement between direct and indirect evidence (in bold). G+CS; glucosamine+chondroitin sulfate; G, glucosamine; CS, chondroitin sulfate; UC-II, undenatured type II collagen; G+CS+MA, glucosamine+chondroitin sulfate+manganese ascorbate; G+O+Celecoxib, glucosamine+ozone+celecoxib; G+Celecoxib, glucosamine+celecoxib; G+omega-3, glucosamine+omega-3; G+CGM, glucosamine+Curcumin derivatives; G+CS+MSM, glucosamine+chondroitin sulfate+methylsulfonylmethane; D-002, inhibitor of both cyclooxygenase and 5-lipoxygenase activities; G+Ibuprofen, glucosamine+ibuprofen; G+MSM, glucosamine+methylsulfonylmethane; MSM, methylsulfonylmethane; G+CS+HA, glucosamine+chondroitin sulfate+hyaluronic acid; G+Celecoxib, glucosamine+celecoxib.

Table S7. Results of global inconsistency tests for the primary analyses.

Time-point	Pain
Post-intervention (Overall)	$Chi^2 = 3.39$, $p = 0.9921$
Short-term treatment	$Chi^2 = 2.03$, $p = 0.9168$
Long-term treatment	$Chi^2 = 1.22$, $p = 0.9761$

Sensitivity analysis for statistical inconsistency

Table S8. Sensitivity analysis of the side-splitting method for statistical inconsistency in overall pain.

Side	Direct		Indirect		Difference		*p*	tau
	Coef.	Std. Err.	Coef.	Std. Err.	Coef.	Std. Err.		
**Placebo UC-II**	-0.72436	0.495484	-0.30062	0.512855	-0.42374	0.71298	0.552	0.45439
**CGM G**	0.502619	0.522843	0.98803	0.584579	-0.48541	0.784281	0.536	0.452119
**CGM G+CS**	1.150501	0.519471	0.664792	0.587553	0.485709	0.784262	0.536	0.452118
**CS Placebo**	0.023906	0.33598	0.214542	0.64636	-0.19064	0.728477	0.794	0.464784
**CS Celecoxib**	-0.14454	0.470789	0.122095	0.617219	-0.26664	0.776273	0.731	0.464054
**CS G**	0.002439	0.296643	-1.52175	0.896804	1.524192	0.944583	0.107	0.407812
**CS G+CS**	-0.02265	0.331596	0.376283	0.618534	-0.39894	0.701802	0.57	0.458462
**Celecoxib Placebo**	0.138235	0.47341	0.086499	0.449574	0.051736	0.652887	0.937	0.46666
**Celecoxib G**	0.122858	0.46094	-0.40433	0.526174	0.527184	0.699519	0.451	0.454051
**Celecoxib G+CS**	0.014327	0.325496	0.607691	0.729413	-0.59336	0.798749	0.458	0.452676
**Celecoxib G+Celecoxib**	-2.45305	0.36107	0.260802	25.82479	-2.71385	25.82733	0.916	0.440298
**D-002 G+CS**	0.52699	0.51285	0.00512	63.24938	0.52187	63.25146	0.993	0.440267
**Diacerein G+CS**	-2.89256	0.57984	0.005243	63.25347	-2.8978	63.25613	0.963	0.440267
**G Placebo**	0.240622	0.284295	0.127328	0.572271	0.113294	0.638399	0.859	0.463073
**G G+CS**	-0.02535	0.313885	0.570768	0.380736	-0.59612	0.493427	0.227	0.432838
**G G+Ibuprofen**	-1.73346	0.512045	-0.56681	28.28631	-1.16665	28.29096	0.967	0.440279
**G G+MSM**	-0.45538	0.49629	-1.96577	1.023225	1.510386	1.133555	0.183	0.422745
**G G+omega-3**	-0.2605	0.465445	0.433682	63.26657	-0.69418	63.26829	0.991	0.440269
**G MSM**	0.141326	0.495448	-1.36906	1.018646	1.510386	1.133555	0.183	0.422745
**G+CS Placebo**	-0.04259	0.183385	0.450349	0.615175	-0.49294	0.641829	0.442	0.449544
**G+CS G+CS+MSM**	-0.48816	0.504284	-0.09162	0.951708	-0.39654	1.076442	0.713	0.460677
**G+CS UC-II**	-0.4501	0.363544	-1.00956	0.942088	0.559457	1.00846	0.579	0.456404
**G+CS+HA Placebo**	.	.	.	.	.	.	.	.

Side	Direct		Indirect		Difference		*p*	tau
	Coef.	Std. Err.	Coef.	Std. Err.	Coef.	Std. Err.		
**G+CS+MA Placebo**	.	.	.	.	.	.	.	.
**G+CS+MSM Placebo**	0.312925	0.503389	0.709466	0.953128	-0.39654	1.076442	0.713	0.460677
**G+Celecoxib G+O+Celecoxib**	-0.31455	0.497501	5.101435	63.26639	-5.41598	63.26827	0.932	0.440286
**G+Ibuprofen Ibuprofen**	2.103488	0.548222	3.895955	63.26336	-1.79247	63.26574	0.977	0.440275
**G+MSM Placebo**	1.256228	0.504767	-0.25416	1.010707	1.510386	1.133555	0.183	0.422744
**G+MSM MSM**	.	.	.	.	.	.	.	.
**MSM Placebo**	0.65952	0.499698	-0.85087	1.012399	1.510386	1.133555	0.183	0.422745

For each comparison the direct and indirect estimates are provided along with the respective *p*-values of differences. *p*-values < 0.10 indicate significant disagreement between direct and indirect evidence. G+CS; glucosamine+chondroitin sulfate; G, glucosamine; CS, chondroitin sulfate; UC-II, undenatured type II collagen; G+CS+MA, glucosamine+chondroitin sulfate+manganese ascorbate; G+O+Celecoxib, glucosamine+ozone+celecoxib; G+Celecoxib, glucosamine+celecoxib; G+omega-3, glucosamine+omega-3; G+CGM, glucosamine+Curcumin derivatives; G+CS+MSM, glucosamine+chondroitin sulfate+methylsulfonylmethane; D-002, inhibitor of both cyclooxygenase and 5-lipoxygenase activities; G+Ibuprofen, glucosamine+ibuprofen; G+MSM, glucosamine+methylsulfonylmethane; MSM, methylsulfonylmethane; G+CS+HA, glucosamine+chondroitin sulfate+hyaluronic acid; G+Celecoxib, glucosamine+celecoxib.

Table S9. Sensitivity analysis of the side-splitting method for statistical inconsistency in short-term pain.

Side	Direct		Indirect		Difference		*p*	tau
	Coef.	Std. Err.	Coef.	Std. Err.	Coef.	Std. Err.		
Placebo UC-II	-0.5612	0.627476	-0.36701	0.701192	-0.19419	0.940917	0.836	0.596113
CGM Placebo	1.174855	0.654724	1.210266	0.664972	-0.03541	0.933444	0.97	0.596621
CGM G	0.434213	0.650823	0.393192	0.971233	0.041021	1.169002	0.972	0.596729
CGM G+CGM	0.745763	0.652216	0.758414	1.773159	-0.01265	1.891434	0.995	0.596903
CGM G+CS	1.150501	0.649413	1.143906	0.687554	0.006596	0.945763	0.994	0.596908
Celecoxib G+CS	0.233482	0.558159	-1.63801	17.54835	1.871487	17.55722	0.915	0.551204
Celecoxib G+Celecoxib	-2.48655	0.43078	0.484132	25.83732	-2.97068	25.84092	0.908	0.551199
D-002 G+CS	0.52699	0.610659	-0.08911	63.25818	0.616099	63.26113	0.992	0.551113
Diacerein G+CS	-1.11054	0.617699	-0.08899	63.25258	-1.02155	63.2556	0.987	0.551112
G Placebo	0.770695	0.461735	0.772145	1.825826	-0.00145	1.884685	0.999	0.596588
G G+CGM	0.311549	0.650109	0.41946	1.669546	-0.10791	1.792141	0.952	0.596283
G G+Ibuprofen	-1.73346	0.610001	0.101086	28.29323	-1.83454	28.29983	0.948	0.551141
G G+MSM	-0.45538	0.650499	-0.56833	1.672523	0.112948	1.792243	0.95	0.596287
G MSM	0.141326	0.649857	0.028377	1.669726	0.112948	1.792244	0.95	0.596287
G+CGM Placebo	0.429193	0.650446	0.511712	1.339287	-0.08252	1.490718	0.956	0.596329
G+CS Placebo	0.038501	0.318551	0.084158	0.789139	-0.04566	0.850903	0.957	0.596776
G+CS G+CS+MSM	-0.48816	0.626015	0.076274	1.269433	-0.56444	1.414932	0.69	0.591452
G+CS UC-II	-0.40083	0.454786	-0.66347	1.285409	0.262644	1.36275	0.847	0.597677
G+CS+HA Placebo	.	.	.	.	.	.	.	.
G+CS+MA Placebo	.	.	.	.	.	.	.	.
G+CS+MSM Placebo	0.312925	0.625294	0.877362	1.270498	-0.56444	1.414932	0.69	0.591452
G+Celecoxib G+O+Celecoxib	-0.31455	0.597853	5.50217	63.26156	-5.81672	63.26432	0.927	0.551155
G+Ibuprofen Ibuprofen	2.103488	0.640665	5.008394	63.27359	-2.90491	63.27683	0.963	0.551132
G+MSM Placebo	1.256228	0.65699	1.143279	1.664894	0.112948	1.792243	0.95	0.596287
G+MSM MSM	.	.	.	.	.	.	.	.

Side	Direct		Indirect		Difference		*p*	tau
	Coef.	Std. Err.	Coef.	Std. Err.	Coef.	Std. Err.		
MSM Placebo	0.65952	0.653103	0.546571	1.665922	0.112948	1.792244	0.95	0.596287

For each comparison the direct and indirect estimates are provided along with the respective *p*-values of differences. *p*-values < 0.10 indicate significant disagreement between direct and indirect evidence. G+CS; glucosamine+chondroitin sulfate; G, glucosamine; CS, chondroitin sulfate; UC-II, undenatured type II collagen; G+CS+MA, glucosamine+chondroitin sulfate+manganese ascorbate; G+O+Celecoxib, glucosamine+ozone+celecoxib; G+Celecoxib, glucosamine+celecoxib; G+omega-3, glucosamine+omega-3; G+CGM, glucosamine+Curcumin derivatives; G+CS+MSM, glucosamine+chondroitin sulfate+methylsulfonylmethane; D-002, inhibitor of both cyclooxygenase and 5-lipoxygenase activities; G+Ibuprofen, glucosamine+ibuprofen; G+MSM, glucosamine+methylsulfonylmethane; MSM, methylsulfonylmethane; G+CS+HA, glucosamine+chondroitin sulfate+hyaluronic acid; G+Celecoxib, glucosamine+celecoxib.

Table S10. Sensitivity analysis of global inconsistency tests for the primary analyses.

Time-point	Pain
Post-intervention (Overall)	$Chi^2 = 1.02$, $p = 0.9998$
Short-term treatment	$Chi^2 = 0.08$, $p = 0.9999$

Confidence rating for pain using CINeMA

CINeMA (Confidence in Network Meta-Analysis) tool (https://cinema.ispm. unibe.ch): CINeMA is an online application that facilitate the evaluation of confidence in the findings from every comparison in the network. It considers 6 domains that possibility affects the level of confidence in the NMA results: (i) within-study bias, (ii) reporting bias, (iii) indirectness, (iv) imprecision, (v) heterogeneity, and (vi) incoherence.

By default, the confidence rating for each comparison in CINeMA is "“high confidence”. Rating the confidence in each comparison were evaluated as per following steps:

Step 1: We assigned a point scale to the domain-level judgments:

- “no concerns” = 0 points,
- “some concerns” = 0.5 points, and
- “major concerns” = 1 point.

Step 2: For each comparison, we downgraded the confidence rating by:

- One level (“moderate confidence”), when there was a reduction of ≥1 but <2 points across all domains.
- Two levels (“low confidence”), when there was a reduction of ≥2 but <3 points across all domains.
- Three levels (“very low confidence”), when there was a reduction of ≥3 points across all domains.

Table S11. Confidence rating for overall pain using CINeMA.

Comparison	χ	Within-study bias	Reporting bias	Indirectness	Imprecision	Heterogeneity	Incoherence	Confidence rating
A:F	1	Some concerns	Some concerns	No concerns	No concerns	Some concerns	No concerns	Moderate
A:G	1	Some concerns	High risk	No concerns	No concerns	No concerns	Some concerns	Low
A:H	1	Some concerns	Some concerns	No concerns	No concerns	Some concerns	No concerns	Moderate
A:S	1	Some concerns	Some concerns	No concerns	No concerns	Some concerns	No concerns	Moderate
B:C	1	No concerns	Some concerns	No concerns	Major concerns	No concerns	No concerns	Moderate
B:F	2	No concerns	Some concerns	No concerns	Some concerns	Some concerns	No concerns	Moderate
B:H	2	No concerns	Some concerns	No concerns	Major concerns	No concerns	No concerns	Moderate
B:S	2	No concerns	Some concerns	No concerns	Some concerns	Some concerns	No concerns	Moderate
C:F	1	No concerns	Some concerns	No concerns	Major concerns	No concerns	No concerns	Moderate
C:H	2	No concerns	Some concerns	No concerns	Some concerns	Some concerns	No concerns	Moderate
C:L	2	Some concerns	Some concerns	No concerns	No concerns	No concerns	No concerns	Moderate
C:S	1	No concerns	Some concerns	No concerns	Some concerns	Some concerns	No concerns	Moderate
D:H	1	Major concerns	Low risk	No concerns	Some concerns	Some concerns	No concerns	Low
E:H	1	No concerns	Low risk	No concerns	No concerns	No concerns	No concerns	High
F:G	1	Some concerns	Some concerns	No concerns	No concerns	Some concerns	No concerns	Moderate
F:H	2	No concerns	Some concerns	No concerns	Some concerns	Some concerns	No concerns	Moderate
F:M	1	Some concerns	Low risk	No concerns	No concerns	No concerns	No concerns	Moderate
F:N	1	No concerns	Some concerns	No concerns	Some concerns	No concerns	No concerns	Moderate
F:P	1	No concerns	High risk	No concerns	Major concerns	No concerns	No concerns	Low
F:R	1	No concerns	Some concerns	No concerns	Major concerns	No concerns	No concerns	Moderate
F:S	4	No concerns	Some concerns	No concerns	Some concerns	Some concerns	No concerns	Moderate
G:H	1	Some concerns	Some concerns	No concerns	No concerns	Some concerns	Major concerns	Low
G:S	1	Some concerns	Some concerns	No concerns	Some concerns	Some concerns	Major concerns	Very low
H:K	1	Some concerns	Some concerns	No concerns	Some concerns	Some concerns	No concerns	Low
H:S	7	No concerns	Some concerns	No concerns	No concerns	Major concerns	Some concerns	Low
H:T	2	No concerns	Some concerns	No concerns	Some concerns	Some concerns	No concerns	Moderate

Comparison	χ	Within-study bias	Reporting bias	Indirectness	Imprecision	Heterogeneity	Incoherence	Confidence rating
I:S	2	No concerns	Low risk	No concerns	Some concerns	Some concerns	No concerns	Moderate
J:S	1	No concerns	Low risk	No concerns	No concerns	No concerns	No concerns	High
K:S	1	Some concerns	Some concerns	No concerns	Some concerns	Some concerns	No concerns	Low
L:O	1	Some concerns	Low risk	No concerns	Major concerns	No concerns	No concerns	Moderate
M:Q	1	Some concerns	Low risk	No concerns	No concerns	No concerns	No concerns	Moderate
N:R	1	No concerns	High risk	No concerns	Some concerns	Some concerns	No concerns	Low
N:S	1	No concerns	Some concerns	No concerns	No concerns	Some concerns	No concerns	Moderate
R:S	1	No concerns	Some concerns	No concerns	Some concerns	Some concerns	No concerns	Moderate
S:T	1	No concerns	Some concerns	No concerns	No concerns	Some concerns	No concerns	Moderate
A:B	0	Some concerns	Some concerns	No concerns	No concerns	Some concerns	No concerns	Moderate
A:C	0	Some concerns	Some concerns	No concerns	No concerns	Some concerns	No concerns	Moderate
A:D	0	Some concerns	Some concerns	No concerns	Major concerns	No concerns	No concerns	Low
A:E	0	Some concerns	Some concerns	No concerns	No concerns	No concerns	No concerns	Moderate
A:I	0	Some concerns	Some concerns	No concerns	No concerns	No concerns	No concerns	Moderate
A:J	0	Some concerns	Some concerns	No concerns	No concerns	No concerns	No concerns	Moderate
A:K	0	Some concerns	Some concerns	No concerns	Major concerns	No concerns	No concerns	Low
A:L	0	Some concerns	Some concerns	No concerns	No concerns	No concerns	No concerns	Moderate
A:M	0	Some concerns	Some concerns	No concerns	No concerns	Some concerns	No concerns	Moderate
A:N	0	Some concerns	High risk	No concerns	Major concerns	No concerns	No concerns	Low
A:O	0	Some concerns	Some concerns	No concerns	No concerns	No concerns	No concerns	Moderate
A:P	0	Some concerns	High risk	No concerns	Major concerns	No concerns	No concerns	Low
A:Q	0	Some concerns	Some concerns	No concerns	Some concerns	Some concerns	No concerns	Low
A:R	0	Some concerns	High risk	No concerns	Some concerns	Some concerns	No concerns	Low
A:T	0	Some concerns	Some concerns	No concerns	Major concerns	No concerns	No concerns	Low
B:D	0	Some concerns	Some concerns	No concerns	Major concerns	No concerns	No concerns	Low
B:E	0	No concerns	Some concerns	No concerns	No concerns	No concerns	No concerns	Moderate
B:G	0	Some concerns	Some concerns	No concerns	Some concerns	Some concerns	No concerns	Low
B:I	0	No concerns	Some concerns	No concerns	Some concerns	Some concerns	No concerns	Moderate

Comparison	χ	Within-study bias	Reporting bias	Indirectness	Imprecision	Heterogeneity	Incoherence	Confidence rating
B:J	0	No concerns	Some concerns	No concerns	No concerns	No concerns	No concerns	Moderate
B:K	0	No concerns	Some concerns	No concerns	Major concerns	No concerns	No concerns	Moderate
B:L	0	No concerns	Some concerns	No concerns	No concerns	No concerns	No concerns	Moderate
B:M	0	No concerns	Some concerns	No concerns	No concerns	No concerns	No concerns	Moderate
B:N	0	No concerns	Some concerns	No concerns	Some concerns	No concerns	No concerns	Moderate
B:O	0	Some concerns	Some concerns	No concerns	No concerns	No concerns	No concerns	Moderate
B:P	0	No concerns	Some concerns	No concerns	Major concerns	No concerns	No concerns	Moderate
B:Q	0	Some concerns	Some concerns	No concerns	Major concerns	No concerns	No concerns	Low
B:R	0	No concerns	Some concerns	No concerns	Major concerns	No concerns	No concerns	Moderate
B:T	0	No concerns	Some concerns	No concerns	Some concerns	Some concerns	No concerns	Moderate
C:D	0	Some concerns	Some concerns	No concerns	Major concerns	No concerns	No concerns	Low
C:E	0	No concerns	Some concerns	No concerns	No concerns	No concerns	No concerns	Moderate
C:G	0	Some concerns	Some concerns	No concerns	Some concerns	Some concerns	No concerns	Low
C:I	0	No concerns	Some concerns	No concerns	Some concerns	Some concerns	No concerns	Moderate
C:J	0	No concerns	Some concerns	No concerns	No concerns	No concerns	No concerns	Moderate
C:K	0	No concerns	Some concerns	No concerns	Major concerns	No concerns	No concerns	Moderate
C:M	0	No concerns	Some concerns	No concerns	No concerns	No concerns	No concerns	Moderate
C:N	0	No concerns	Some concerns	No concerns	Some concerns	Some concerns	No concerns	Moderate
C:O	0	Some concerns	Some concerns	No concerns	No concerns	No concerns	No concerns	Moderate
C:P	0	No concerns	Some concerns	No concerns	Major concerns	No concerns	No concerns	Moderate
C:Q	0	Some concerns	Some concerns	No concerns	Major concerns	No concerns	No concerns	Low
C:R	0	No concerns	Some concerns	No concerns	Major concerns	No concerns	No concerns	Moderate
C:T	0	No concerns	Some concerns	No concerns	Some concerns	Some concerns	No concerns	Moderate
D:E	0	Some concerns	Low risk	No concerns	No concerns	No concerns	No concerns	Moderate
D:F	0	Some concerns	Some concerns	No concerns	Major concerns	No concerns	No concerns	Low
D:G	0	Some concerns	Some concerns	No concerns	No concerns	Some concerns	No concerns	Moderate
D:I	0	Some concerns	Low risk	No concerns	Some concerns	Some concerns	No concerns	Moderate
D:J	0	Some concerns	Low risk	No concerns	No concerns	No concerns	No concerns	Moderate

Comparison	χ	Within-study bias	Reporting bias	Indirectness	Imprecision	Heterogeneity	Incoherence	Confidence rating
D:K	0	Some concerns	Low risk	No concerns	Major concerns	No concerns	No concerns	Moderate
D:L	0	Some concerns	Low risk	No concerns	No concerns	No concerns	No concerns	Moderate
D:M	0	Some concerns	Low risk	No concerns	No concerns	Some concerns	No concerns	Moderate
D:N	0	Some concerns	Some concerns	No concerns	Major concerns	No concerns	No concerns	Low
D:O	0	Some concerns	Low risk	No concerns	No concerns	No concerns	No concerns	Moderate
D:P	0	Some concerns	Some concerns	No concerns	Major concerns	No concerns	No concerns	Low
D:Q	0	Some concerns	Low risk	No concerns	Major concerns	No concerns	No concerns	Moderate
D:R	0	Some concerns	Some concerns	No concerns	Major concerns	No concerns	No concerns	Low
D:S	0	Some concerns	Some concerns	No concerns	Some concerns	Some concerns	No concerns	Low
D:T	0	Some concerns	Some concerns	No concerns	Major concerns	No concerns	No concerns	Low
E:F	0	No concerns	Some concerns	No concerns	No concerns	No concerns	No concerns	Moderate
E:G	0	No concerns	Some concerns	No concerns	No concerns	No concerns	No concerns	Moderate
E:I	0	No concerns	Low risk	No concerns	No concerns	No concerns	No concerns	High
E:J	0	No concerns	Low risk	No concerns	No concerns	No concerns	No concerns	High
E:K	0	No concerns	Low risk	No concerns	No concerns	No concerns	No concerns	High
E:L	0	No concerns	Low risk	No concerns	No concerns	No concerns	No concerns	High
E:M	0	No concerns	Low risk	No concerns	No concerns	No concerns	No concerns	High
E:N	0	No concerns	Some concerns	No concerns	No concerns	No concerns	No concerns	Moderate
E:O	0	No concerns	Low risk	No concerns	No concerns	No concerns	No concerns	High
E:P	0	No concerns	Some concerns	No concerns	No concerns	No concerns	No concerns	Moderate
E:Q	0	No concerns	Low risk	No concerns	No concerns	No concerns	No concerns	High
E:R	0	No concerns	Some concerns	No concerns	No concerns	No concerns	No concerns	Moderate
E:S	0	No concerns	Some concerns	No concerns	No concerns	No concerns	No concerns	Moderate
E:T	0	No concerns	Some concerns	No concerns	No concerns	No concerns	No concerns	Moderate
F:I	0	No concerns	Some concerns	No concerns	Some concerns	Some concerns	No concerns	Moderate
F:J	0	No concerns	Some concerns	No concerns	No concerns	No concerns	No concerns	Moderate
F:K	0	Some concerns	Some concerns	No concerns	Major concerns	No concerns	No concerns	Low
F:L	0	No concerns	Some concerns	No concerns	No concerns	No concerns	No concerns	Moderate

Comparison	χ	Within-study bias	Reporting bias	Indirectness	Imprecision	Heterogeneity	Incoherence	Confidence rating
F:O	0	Some concerns	Some concerns	No concerns	No concerns	No concerns	No concerns	Moderate
F:Q	0	Some concerns	Some concerns	No concerns	Major concerns	No concerns	No concerns	Low
F:T	0	No concerns	Some concerns	No concerns	Some concerns	Some concerns	No concerns	Moderate
G:I	0	No concerns	Some concerns	No concerns	Major concerns	No concerns	No concerns	Moderate
G:J	0	No concerns	Some concerns	No concerns	No concerns	No concerns	No concerns	Moderate
G:K	0	Some concerns	Some concerns	No concerns	No concerns	Some concerns	No concerns	Moderate
G:L	0	Some concerns	Some concerns	No concerns	No concerns	No concerns	No concerns	Moderate
G:M	0	Some concerns	Some concerns	No concerns	No concerns	No concerns	No concerns	Moderate
G:N	0	No concerns	High risk	No concerns	No concerns	No concerns	No concerns	Moderate
G:O	0	Some concerns	Some concerns	No concerns	No concerns	No concerns	No concerns	Moderate
G:P	0	No concerns	High risk	No concerns	No concerns	Some concerns	No concerns	Moderate
G:Q	0	Some concerns	Some concerns	No concerns	Major concerns	No concerns	No concerns	Low
G:R	0	No concerns	High risk	No concerns	Some concerns	Some concerns	No concerns	Low
G:T	0	Some concerns	Some concerns	No concerns	No concerns	Some concerns	No concerns	Moderate
H:I	0	No concerns	Some concerns	No concerns	Some concerns	Some concerns	No concerns	Moderate
H:J	0	No concerns	Some concerns	No concerns	No concerns	No concerns	No concerns	Moderate
H:L	0	No concerns	Some concerns	No concerns	No concerns	No concerns	No concerns	Moderate
H:M	0	No concerns	Some concerns	No concerns	No concerns	No concerns	No concerns	Moderate
H:N	0	No concerns	Some concerns	No concerns	No concerns	Some concerns	No concerns	Moderate
H:O	0	Some concerns	Some concerns	No concerns	No concerns	No concerns	No concerns	Moderate
H:P	0	No concerns	Some concerns	No concerns	Major concerns	No concerns	No concerns	Moderate
H:Q	0	Some concerns	Some concerns	No concerns	Major concerns	No concerns	No concerns	Low
H:R	0	No concerns	Some concerns	No concerns	Major concerns	No concerns	No concerns	Moderate
I:J	0	No concerns	Low risk	No concerns	No concerns	No concerns	No concerns	High
I:K	0	No concerns	Low risk	No concerns	Some concerns	Some concerns	No concerns	Moderate
I:L	0	No concerns	Low risk	No concerns	No concerns	No concerns	No concerns	High
I:M	0	No concerns	Low risk	No concerns	No concerns	No concerns	No concerns	High
I:N	0	No concerns	Some concerns	No concerns	No concerns	Some concerns	No concerns	Moderate

Comparison	χ	Within-study bias	Reporting bias	Indirectness	Imprecision	Heterogeneity	Incoherence	Confidence rating
I:O	0	No concerns	Low risk	No concerns	No concerns	No concerns	No concerns	High
I:P	0	No concerns	Some concerns	No concerns	Some concerns	Some concerns	No concerns	Moderate
I:Q	0	Some concerns	Low risk	No concerns	Major concerns	No concerns	No concerns	Moderate
I:R	0	No concerns	Some concerns	No concerns	Some concerns	Some concerns	No concerns	Moderate
I:T	0	No concerns	Some concerns	No concerns	No concerns	Some concerns	No concerns	Moderate
J:K	0	No concerns	Low risk	No concerns	No concerns	No concerns	No concerns	High
J:L	0	No concerns	Low risk	No concerns	Major concerns	No concerns	No concerns	Moderate
J:M	0	No concerns	Low risk	No concerns	Major concerns	No concerns	No concerns	Moderate
J:N	0	No concerns	Some concerns	No concerns	No concerns	No concerns	No concerns	Moderate
J:O	0	No concerns	Low risk	No concerns	Major concerns	No concerns	No concerns	Moderate
J:P	0	No concerns	Some concerns	No concerns	No concerns	No concerns	No concerns	Moderate
J:Q	0	Some concerns	Low risk	No concerns	No concerns	No concerns	No concerns	Moderate
J:R	0	No concerns	Some concerns	No concerns	No concerns	No concerns	No concerns	Moderate
J:T	0	No concerns	Some concerns	No concerns	No concerns	No concerns	No concerns	Moderate
K:L	0	Some concerns	Low risk	No concerns	No concerns	No concerns	No concerns	Moderate
K:M	0	Some concerns	Low risk	No concerns	No concerns	No concerns	No concerns	Moderate
K:N	0	No concerns	Some concerns	No concerns	Major concerns	No concerns	No concerns	Moderate
K:O	0	Some concerns	Low risk	No concerns	No concerns	No concerns	No concerns	Moderate
K:P	0	No concerns	Some concerns	No concerns	Major concerns	No concerns	No concerns	Moderate
K:Q	0	Some concerns	Low risk	No concerns	Major concerns	No concerns	No concerns	Moderate
K:R	0	No concerns	Some concerns	No concerns	Major concerns	No concerns	No concerns	Moderate
K:T	0	No concerns	Low risk	No concerns	Major concerns	No concerns	No concerns	Moderate
L:M	0	Some concerns	Low risk	No concerns	Major concerns	No concerns	No concerns	Moderate
L:N	0	No concerns	Low risk	No concerns	No concerns	No concerns	No concerns	High
L:P	0	No concerns	Low risk	No concerns	No concerns	No concerns	No concerns	High
L:Q	0	Some concerns	Low risk	No concerns	No concerns	No concerns	No concerns	Moderate
L:R	0	No concerns	Low risk	No concerns	No concerns	No concerns	No concerns	High
L:S	0	No concerns	Low risk	No concerns	No concerns	No concerns	No concerns	High

Comparison	χ	Within-study bias	Reporting bias	Indirectness	Imprecision	Heterogeneity	Incoherence	Confidence rating
L:T	0	No concerns	Some concerns	No concerns	No concerns	No concerns	No concerns	Moderate
M:N	0	No concerns	Some concerns	No concerns	Some concerns	Some concerns	No concerns	Moderate
M:O	0	Some concerns	Low risk	No concerns	Major concerns	No concerns	No concerns	Moderate
M:P	0	Some concerns	Some concerns	No concerns	No concerns	No concerns	No concerns	Moderate
M:R	0	No concerns	Some concerns	No concerns	No concerns	No concerns	No concerns	Moderate
M:S	0	Some concerns	Some concerns	No concerns	No concerns	No concerns	No concerns	Moderate
M:T	0	No concerns	Some concerns	No concerns	No concerns	No concerns	No concerns	Moderate
N:O	0	No concerns	Some concerns	No concerns	No concerns	No concerns	No concerns	Moderate
N:P	0	No concerns	High risk	No concerns	Major concerns	No concerns	No concerns	Low
N:Q	0	Some concerns	Some concerns	No concerns	Some concerns	Some concerns	No concerns	Low
N:T	0	No concerns	Some concerns	No concerns	Major concerns	No concerns	No concerns	Moderate
O:P	0	No concerns	Low risk	No concerns	No concerns	No concerns	No concerns	High
O:Q	0	Some concerns	Low risk	No concerns	No concerns	No concerns	No concerns	Moderate
O:R	0	No concerns	Some concerns	No concerns	No concerns	No concerns	No concerns	Moderate
O:S	0	Some concerns	Some concerns	No concerns	No concerns	No concerns	No concerns	Moderate
O:T	0	No concerns	Some concerns	No concerns	No concerns	No concerns	No concerns	Moderate
P:Q	0	Some concerns	Some concerns	No concerns	Major concerns	No concerns	No concerns	Low
P:R	0	No concerns	High risk	No concerns	Major concerns	No concerns	No concerns	Low
P:S	0	No concerns	Some concerns	No concerns	Some concerns	Some concerns	No concerns	Moderate
P:T	0	No concerns	Some concerns	No concerns	Major concerns	No concerns	No concerns	Moderate
Q:R	0	Some concerns	Some concerns	No concerns	Major concerns	No concerns	No concerns	Low
Q:S	0	Some concerns	Some concerns	No concerns	Major concerns	No concerns	No concerns	Low
Q:T	0	Some concerns	Some concerns	No concerns	Major concerns	No concerns	No concerns	Low
R:T	0	No concerns	Some concerns	No concerns	Major concerns	No concerns	No concerns	Moderate

A: CGM (curcumin derivatives); B: CS (chondroitin sulfate); C: Celecoxib; D: D-002 (inhibitor of both cyclooxygenase and 5-lipoxygenase activities); E: Diacerein; F: G; G: G+CGM (glucosamine+curcumin derivatives); H: G+CS (glucosamine+chondroitin sulfate); I: G+CS+HA (glucosamine+chondroitin sulfate+hyaluronic acid); J: G+CS+MA (glucosamine+chondroitin sulfate+manganese ascorbate); K: G+CS+MSM (glucosamine+chondroitin sulfate+methylsulfonylmethane); L: G+Celecoxib (glucosamine+celecoxib); M: G+Ibuprofen (glucosamine+ibuprofen); N: G+MSM (glucosamine+methylsulfonylmethane); O: G+O+Celecoxib (glucosamine+ozone+celecoxib); P: G+omega-3 (glucosamine+omega-3); Q: Ibuprofen; R: MSM (methylsulfonylmethane); S (reference): Placebo; T: UC-II (undenatured type II collagen).

Results from direct and network evidence for pain

Interpretation of network results: Results in the league tables below are presented as standardised mean differences (SMD), with the corresponding 95% confidence interval (CI). To interpret our results, a negative SMD value indicates that results favoured the column defining treatment node whereas a positive SMD value indicates that results favoured the row defining treatment node for the NMA results (lower left triangle). The significant results are bold and colored with the certainty of evidence assessed for each comparison with CINeMA and classified into high (in green), moderate (in blue), low (in yellow) and very low (in red). A negative SMD value indicates that results favoured the row-defining treatment nodes whereas a positive SMD value indicates that results favoured the column defining treatment node for the pairwise meta-analysis results (upper right triangle).

Table S12. Pairwise and network meta-analysis results for overall pain.

**Placebo (Ref)**	-0.72 (-1.11, -0.34)	0.66 (0.14, 1.18)				1.26 (0.72, 1.80)			0.31 (-0.08, 0.71)	2.64 (2.00, 3.28)	-0.23 (-1.11, 0.66)	-0.04 (-0.22, 0.14)	0.43 (-0.08, 0.94)	0.30 (-0.03, 0.63)			0.14 (-0.02, 0.29)	0.01 (-0.11, 0.14)	1.17 (0.65, 1.70)
0.59 (-0.19, 1.37)	**UC-II**											-0.47 (-0.78, -0.16)							
0.38 (-0.64, 1.41)	-0.21 (-1.48, 1.07)	**MSM**				0.60 (0.08, 1.11)								0.14 (-0.37, 0.65)					
-0.11 (-1.86, 1.63)	-0.70 (-2.59, 1.19)	-0.50 (-2.45, 1.46)	**Ibuprofen**				2.10 (1.46, 2.74)												
0.52 (-0.67, 1.70)	-0.07 (-1.46, 1.32)	0.13 (-1.35, 1.61)	0.63 (-1.35, 2.61)	**G+omega-3**										-0.26 (-0.56, 0.04)					
**2.97 (1.42, 4.53)**	**2.38 (0.68, 4.08)**	**2.59 (0.75, 4.42)**	**3.09 (0.79, 5.38)**	**2.45 (0.55, 4.36)**	**G+O+Celecoxib**			-0.31 (-0.77, 0.14)											

0.98 (-0.06, 2.01)	0.39 (-0.89, 1.67)	0.59 (-0.55, 1.74)	1.09 (-0.87, 3.05)	0.46 (-1.02, 1.94)	**-1.99 (-3.83, -0.15)**	**G+MSM**								-0.46 (-0.96, 0.05)					
**1.99 (0.73,3.25)**	1.40 (-0.05, 2.85)	1.61 (0.07,3.14)	**2.10 (0.89,3.31)**	1.47 (-0.10, 3.04)	-0.98 (-2.93, 0.97)	1.01 (-0.53, 2.55)	**G+Ibuprofen**							-1.73 (-2.25, -1.22)					
**2.66 (1.58,3.73)**	**2.07 (0.79,3.34)**	**2.27 (0.82,3.73)**	**2.77 (0.77,4.78)**	**2.14 (0.60,3.68)**	-0.31 (-1.44, 0.81)	**1.68 (0.22,3.14)**	0.67 (-0.93, 2.27)	**G+Celecoxib**								-2.55 (-4.75, -0.36)			
0.45 (-0.52, 1.42)	-0.14 (-1.35, 1.07)	0.07 (-1.34, 1.47)	0.56 (-1.42, 2.55)	-0.07 (-1.58, 1.45)	**-2.52 (-4.33, -0.72)**	-0.53 (-1.94, 0.88)	-1.54 (-3.11, 0.03)	**-2.21 (-3.62, -0.79)**	**G+CS+MSM**			-0.49 (-0.89, -0.09)							
**2.64 (1.43,3.85)**	**2.05 (0.61,3.49)**	**2.25 (0.67,3.84)**	**2.75 (0.63,4.87)**	**2.12 (0.43,3.81)**	-0.33 (-2.30, 1.64)	1.66 (0.07,3.25)	0.65 (-1.10, 2.39)	-0.02 (-1.64, 1.60)	**2.19 (0.64,3.74)**	**G+CS+MA**									
-0.23 (-1.04, 0.58)	-0.82 (-1.94, 0.30)	-0.61 (-1.92, 0.69)	-0.12 (-2.04, 1.80)	-0.75 (-2.18, 0.69)	**-3.20 (-4.95, -1.45)**	-1.21 (-2.52, 0.10)	**-2.22 (-3.71, -0.73)**	**-2.89 (-4.23, -1.55)**	-0.68 (-1.94, 0.58)	**-2.87 (-4.32, -1.41)**	**G+CS+HA**								
0.10 (-0.28, 0.48)	-0.49 (-1.23, 0.25)	-0.28 (-1.35, 0.78)	0.21 (-1.54, 1.96)	-0.42 (-1.61, 0.78)	**-2.87 (-4.41, -1.34)**	-0.88 (-1.95, 0.19)	**-1.89 (-3.16, -0.62)**	**-2.56 (-3.61, -1.51)**	-0.35 (-1.32, 0.62)	**-2.54 (-3.81, -1.27)**	0.33 (-0.56, 1.22)	**G+CS**	-1.51 (-2.01, -1.01)	-0.05 (-0.19, 0.10)	-2.89 (-3.63, -2.15)	0.53 (0.01, 1.04)	0.01 (-0.10, 0.13)	-0.04 (-0.24, 0.16)	1.15 (0.65, 1.65)
-0.46 (-1.24, 0.33)	-1.04 (-2.09, 0.01)	-0.84 (-2.09, 0.41)	-0.34 (-2.20, 1.52)	-0.97 (-2.32, 0.38)	**-3.43 (-5.12, -1.73)**	-1.43 (-2.69, -0.18)	**-2.44 (-3.86, -1.03)**	**-3.11 (-4.39, -1.84)**	-0.90 (-2.12, 0.31)	**-3.09 (-4.53, -1.65)**	-0.22 (-1.35, 0.90)	-0.55 (-1.32, 0.21)	**G+CGM**	0.31 (-0.20, 0.82)					0.75 (0.23, 1.26)

0.26 (-0.26, 0.77)	-0.33 (-1.22, 0.56)	-0.13 (-1.15, 0.90)	0.37 (-1.30, 2.04)	-0.26 (-1.33, 0.81)	**-2.72 (-4.30, -1.14)**	-0.72 (-1.75, 0.31)	**-1.73 (-2.88, -0.59)**	**-2.40 (-3.51, -1.29)**	-0.19 (-1.27, 0.88)	**-2.38 (-3.70, -1.07)**	0.49 (-0.47, 1.44)	0.16 (-0.38, 0.69)	0.71 (-0.12, 1.54)	**G**			0.12 (-0.03, 0.28)	-0.01 (-0.13, 0.12)	0.43 (-0.08, 0.94)
**-2.79 (-4.11, -1.47)**	**-3.38 (-4.85, -1.91)**	**-3.18 (-4.83, -1.52)**	**-2.68 (-4.84, -0.52)**	**-3.31 (-5.05, -1.57)**	**-5.76 (-7.76, -3.77)**	**-3.77 (-5.43, -2.11)**	**-4.78 (-6.57, -2.99)**	**-5.45 (-7.09, -3.81)**	**-3.24 (-4.84, -1.65)**	**-5.43 (-7.22, -3.64)**	**-2.56 (-4.11, -1.02)**	**-2.89 (-4.16, -1.63)**	**-2.34 (-3.82, -0.86)**	**-3.05 (-4.42, -1.68)**	**Diacerein**				
0.63 (-0.58, 1.84)	0.04 (-1.33, 1.41)	0.24 (-1.33, 1.81)	0.74 (-1.35, 2.83)	0.11 (-1.55, 1.77)	**-2.35 (-4.26, -0.43)**	-0.35 (-1.92, 1.22)	-1.36 (-3.07, 0.35)	**-2.03 (-3.59, -0.48)**	0.18 (-1.33, 1.68)	-2.01 (-3.72, -0.30)	0.86 (-0.60, 2.31)	0.53 (-0.62, 1.68)	1.08 (-0.30, 2.46)	0.37 (-0.90, 1.64)	**3.42 (1.71,5.13)**	**D-002**			
0.18 (-0.53, 0.89)	-0.41 (-1.40, 0.58)	-0.21 (-1.41, 1.00)	0.29 (-1.54, 2.13)	-0.34 (-1.65, 0.98)	**-2.79 (-4.18, -1.41)**	-0.80 (-2.01, 0.42)	**-1.81 (-3.19, -0.43)**	**-2.48 (-3.29, -1.67)**	-0.27 (-1.43, 0.89)	**-2.46 (-3.86, -1.06)**	0.41 (-0.66, 1.48)	0.08 (-0.59, 0.75)	0.63 (-0.35, 1.62)	-0.08 (-0.84, 0.69)	**2.97 (1.54,4.40)**	-0.45 (-1.78, 0.88)	**Celecoxib**	-0.14 (-0.30, 0.01)	
0.11 (-0.53, 0.76)	-0.48 (-1.44, 0.49)	-0.27 (-1.44, 0.90)	0.23 (-1.57, 2.03)	-0.40 (-1.67, 0.87)	**-2.86 (-4.47, -1.25)**	-0.86 (-2.04, 0.31)	**-1.88 (-3.21, -0.54)**	**-2.54 (-3.70, -1.39)**	-0.34 (-1.47, 0.80)	**-2.52 (-3.90, -1.15)**	0.34 (-0.69, 1.38)	0.01 (-0.63, 0.66)	0.57 (-0.38, 1.52)	-0.14 (-0.83, 0.54)	**2.91 (1.49,4.33)**	-0.51 (-1.83, 0.81)	-0.06 (-0.89, 0.76)	**CS**	
0.93 (0.14,1.72)	0.34 (-0.71, 1.39)	0.55 (-0.71, 1.80)	1.04 (-0.82, 2.91)	0.41 (-0.94, 1.77)	**-2.04 (-3.74, -0.34)**	-0.05 (-1.31, 1.21)	-1.06 (-2.47, 0.36)	**-1.73 (-3.00, -0.45)**	0.48 (-0.73, 1.70)	-1.71 (-3.15, -0.26)	1.16 (0.04,2.29)	0.83 (0.07,1.60)	**1.39 (0.45,2.32)**	0.67 (-0.15, 1.50)	**3.72 (2.25,5.20)**	0.30 (-1.08, 1.68)	0.75 (-0.23, 1.74)	0.82 (-0.14, 1.77)	**CGM**

UC-II, undenatured type II collagen; MSM, methylsulfonylmethane; G+omega-3, glucosamine+omega-3; G+O+Celecoxib, glucosamine+ozone+celecoxib; G+MSM, glucosamine+methylsulfonylmethane; G+Ibuprofen, glucosamine+ibuprofen; G+CS+MSM, glucosamine+chondroitin sulfate+methylsulfonylmethane; G+CS+MA, glucosamine+chondroitin sulfate+manganese ascorbate; G+CS+HA, glucosamine+chondroitin sulfate+hyaluronic acid; G+CS; glucosamine+chondroitin sulfate; G, glucosamine; D-002, inhibitor of both cyclooxygenase and 5-lipoxygenase activities; D-002, inhibitor of both cyclooxygenase and 5-lipoxygenase activities; CGM, curcumin derivatives.

Rank results for pain

Table S13. SUCRA and mean rank results for overall pain.

Treatment	SUCRA	PrBest	MeanRank
CGM	27.2	0	14.8
CS	68.1	0	7.1
Celecoxib	34.3	0	13.5
D-002	37.8	0	12.8
Diacerein	54.8	0	9.6
G	0	0	20
G+CGM	42.1	0	12
G+CS	13.2	0	17.5
G+CS+HA	33.1	0	13.7
G+CS+MA	21.4	0	15.9
G+CS+MSM	92.4	29.5	2.4
G+Celecoxib	49.8	0	10.5
G+Ibuprofen	92.4	13.3	2.4
G+MSM	85.6	5.8	3.7
G+O+Celecoxib	67.7	0	7.1
G+omega-3	95.5	51.4	1.9
Ibuprofen	51.2	0	10.3
MSM	30.6	0	14.2
Placebo	46.4	0	11.2
UC-II	56.3	0	9.3

UC-II, undenatured type II collagen; MSM, methylsulfonylmethane; G+omega-3, glucosamine+omega-3; G+O+Celecoxib, glucosamine+ozone+celecoxib; G+MSM, glucosamine+methylsulfonylmethane; G+Ibuprofen, glucosamine+ibuprofen; G+Celecoxib, glucosamine+celecoxib; G+CS+MSM, glucosamine+chondroitin sulfate+methylsulfonylmethane; G+CS+MA, glucosamine+chondroitin sulfate+manganese ascorbate; G+CS+HA, glucosamine+chondroitin sulfate+hyaluronic acid; G+CS; glucosamine+chondroitin sulfate; G, glucosamine; D-002, inhibitor of both cyclooxygenase and 5-lipoxygenase activities; D-002, inhibitor of both cyclooxygenase and 5-lipoxygenase activities; CGM, curcumin derivatives

Table S14. Studies excluded from the network meta-analysis for overall pain.

Study	Treatment nodes	Pain [MD (95%CI)]	Pain [SMD (95%CI)]
⁋Kongtharvonskul, 2016	G+Diacerein vs G+Placebo	0.26 (-2.24, 2.76)	0.04 (-0.31, 0.38)
⁋Kumar, 2020	G+Oxaceprol vs Oxaceprol	-1.70 (-5.64, 2.24)	-0.26 (-0.89, 0.36)
⁋Sun, 2020	G+Etoricoxib vs Etoricoxib	-3.63 (-4.05, -3.21)	-3.03 (-3.60, -2.46)
⁋Zhijun, 2019	G+Meloxicam vs Meloxicam	-1.31 (-1.76, -0.86)	-1.21 (-1.66, -0.75)

⁋Disconnected studies from the network

MD: Mean difference; CI: confidence interval; SMD: standardized mean difference

Table S15. Confidence rating for short-term pain using CINeMA.

Comparison	χ	Within-study bias	Reporting bias	Indirectness	Imprecision	Heterogeneity	Incoherence	Confidence rating
A:F	1	Some concerns	High risk	No concerns	Some concerns	Some concerns	Major concerns	Very low
A:G	2	Some concerns	Some concerns	No concerns	Some concerns	Some concerns	No concerns	Low
A:S	1	Some concerns	High risk	No concerns	No concerns	Major concerns	No concerns	Low
B:G	1	No concerns	High risk	No concerns	Major concerns	No concerns	No concerns	Low
B:K	2	Some concerns	Low risk	No concerns	No concerns	No concerns	No concerns	Moderate
C:G	1	Major concerns	Low risk	No concerns	Major concerns	No concerns	No concerns	Low
D:G	1	No concerns	Low risk	No concerns	Some concerns	Some concerns	No concerns	Moderate
E:L	1	Some concerns	Low risk	No concerns	No concerns	Major concerns	No concerns	Moderate
E:M	1	No concerns	High risk	No concerns	Major concerns	No concerns	No concerns	Low
E:P	1	No concerns	High risk	No concerns	Major concerns	No concerns	No concerns	Low
E:Q	1	No concerns	High risk	No concerns	Major concerns	No concerns	No concerns	Low
F:G	2	Some concerns	Some concerns	No concerns	Major concerns	No concerns	No concerns	Low
F:S	1	Some concerns	High risk	No concerns	Major concerns	No concerns	No concerns	Low
G:J	1	Some concerns	Some concerns	No concerns	Major concerns	No concerns	No concerns	Low
G:Q	3	No concerns	Some concerns	No concerns	Major concerns	No concerns	No concerns	Moderate
G:R	2	No concerns	Some concerns	No concerns	Major concerns	No concerns	No concerns	Moderate
G:S	1	Some concerns	Some concerns	No concerns	Major concerns	No concerns	No concerns	Low
H:Q	2	No concerns	Some concerns	No concerns	Major concerns	No concerns	No concerns	Moderate
H:S	1	No concerns	Some concerns	No concerns	Major concerns	No concerns	No concerns	Moderate
I:Q	1	No concerns	Low risk	No concerns	Some concerns	Some concerns	No concerns	Moderate
J:Q	1	Some concerns	Some concerns	No concerns	Major concerns	No concerns	No concerns	Low
K:N	1	Some concerns	Low risk	No concerns	Major concerns	No concerns	No concerns	Moderate
L:O	1	Some concerns	Low risk	No concerns	No concerns	Major concerns	No concerns	Moderate
M:P	1	No concerns	High risk	No concerns	Major concerns	No concerns	No concerns	Low
M:Q	1	No concerns	High risk	No concerns	Some concerns	Some concerns	No concerns	Low
P:Q	1	No concerns	High risk	No concerns	Major concerns	No concerns	No concerns	Low

Compariso n	χ	Within-study bias	Reporting bias	Indirectness	Imprecision	Heterogeneity	Incoherence	Confidence rating
Q:R	1	No concerns	Some concerns	No concerns	Major concerns	No concerns	No concerns	Moderate
A:B	0	Some concerns	Some concerns	No concerns	Major concerns	No concerns	No concerns	Low
A:C	0	Major concerns	Some concerns	No concerns	Major concerns	No concerns	No concerns	Low
A:D	0	Some concerns	Some concerns	No concerns	No concerns	Major concerns	No concerns	Low
A:E	0	Some concerns	Some concerns	No concerns	Major concerns	No concerns	No concerns	Low
A:H	0	Some concerns	Some concerns	No concerns	Some concerns	Some concerns	No concerns	Low
A:I	0	Some concerns	Some concerns	No concerns	Major concerns	No concerns	No concerns	Low
A:J	0	Some concerns	Some concerns	No concerns	Major concerns	No concerns	No concerns	Low
A:K	0	Some concerns	Some concerns	No concerns	Some concerns	Some concerns	No concerns	Low
A:L	0	Some concerns	Some concerns	No concerns	Major concerns	No concerns	No concerns	Low
A:M	0	Some concerns	High risk	No concerns	Major concerns	No concerns	No concerns	Low
A:N	0	Some concerns	Some concerns	No concerns	Some concerns	Some concerns	No concerns	Low
A:O	0	Some concerns	Some concerns	No concerns	Major concerns	No concerns	No concerns	Low
A:P	0	Some concerns	High risk	No concerns	Major concerns	No concerns	No concerns	Low
A:Q	0	Some concerns	Some concerns	No concerns	Major concerns	No concerns	No concerns	Low
A:R	0	Some concerns	Some concerns	No concerns	Major concerns	No concerns	No concerns	Low
B:C	0	Some concerns	Some concerns	No concerns	Major concerns	No concerns	No concerns	Low
B:D	0	No concerns	Some concerns	No concerns	Major concerns	No concerns	No concerns	Moderate
B:E	0	No concerns	Some concerns	No concerns	Major concerns	No concerns	No concerns	Moderate
B:F	0	Some concerns	Some concerns	No concerns	Major concerns	No concerns	No concerns	Low
B:H	0	No concerns	Some concerns	No concerns	Major concerns	No concerns	No concerns	Moderate
B:I	0	No concerns	Some concerns	No concerns	Major concerns	No concerns	No concerns	Moderate
B:J	0	No concerns	Some concerns	No concerns	Major concerns	No concerns	No concerns	Moderate
B:L	0	No concerns	Some concerns	No concerns	Major concerns	No concerns	No concerns	Moderate
B:M	0	No concerns	Some concerns	No concerns	Major concerns	No concerns	No concerns	Moderate
B:N	0	Some concerns	Some concerns	No concerns	No concerns	No concerns	No concerns	Moderate
B:O	0	No concerns	Some concerns	No concerns	Major concerns	No concerns	No concerns	Moderate
B:P	0	No concerns	Some concerns	No concerns	Major concerns	No concerns	No concerns	Moderate

Comparison	χ	Within-study bias	Reporting bias	Indirectness	Imprecision	Heterogeneity	Incoherence	Confidence rating
B:Q	0	No concerns	Some concerns	No concerns	Major concerns	No concerns	No concerns	Moderate
B:R	0	No concerns	Some concerns	No concerns	Major concerns	No concerns	No concerns	Moderate
B:S	0	Some concerns	Some concerns	No concerns	Major concerns	No concerns	No concerns	Low
C:D	0	Some concerns	Low risk	No concerns	Major concerns	No concerns	No concerns	Moderate
C:E	0	Some concerns	Some concerns	No concerns	Major concerns	No concerns	No concerns	Low
C:F	0	Some concerns	Some concerns	No concerns	Major concerns	No concerns	No concerns	Low
C:H	0	Some concerns	Some concerns	No concerns	Major concerns	No concerns	No concerns	Low
C:I	0	Some concerns	Low risk	No concerns	Major concerns	No concerns	No concerns	Moderate
C:J	0	Some concerns	Low risk	No concerns	Major concerns	No concerns	No concerns	Moderate
C:K	0	Some concerns	Low risk	No concerns	Some concerns	Some concerns	No concerns	Moderate
C:L	0	Some concerns	Low risk	No concerns	Major concerns	No concerns	No concerns	Moderate
C:M	0	Some concerns	Some concerns	No concerns	Major concerns	No concerns	No concerns	Low
C:N	0	Some concerns	Low risk	No concerns	Some concerns	Some concerns	No concerns	Moderate
C:O	0	Some concerns	Low risk	No concerns	Major concerns	No concerns	No concerns	Moderate
C:P	0	Some concerns	Some concerns	No concerns	Major concerns	No concerns	No concerns	Low
C:Q	0	Some concerns	Some concerns	No concerns	Major concerns	No concerns	No concerns	Low
C:R	0	Some concerns	Some concerns	No concerns	Major concerns	No concerns	No concerns	Low
C:S	0	Some concerns	Some concerns	No concerns	Major concerns	No concerns	No concerns	Low
D:E	0	No concerns	Some concerns	No concerns	Major concerns	No concerns	No concerns	Moderate
D:F	0	Some concerns	Some concerns	No concerns	Major concerns	No concerns	No concerns	Low
D:H	0	No concerns	Some concerns	No concerns	Major concerns	No concerns	No concerns	Moderate
D:I	0	No concerns	Low risk	No concerns	No concerns	Major concerns	No concerns	Moderate
D:J	0	No concerns	Low risk	No concerns	Major concerns	No concerns	No concerns	Moderate
D:K	0	No concerns	Low risk	No concerns	No concerns	No concerns	No concerns	High
D:L	0	No concerns	Low risk	No concerns	No concerns	Some concerns	No concerns	Moderate
D:M	0	No concerns	Some concerns	No concerns	Some concerns	Some concerns	No concerns	Moderate
D:N	0	Some concerns	Low risk	No concerns	No concerns	No concerns	No concerns	Moderate
D:O	0	No concerns	Low risk	No concerns	Major concerns	No concerns	No concerns	Moderate

Comparison	χ	Within-study bias	Reporting bias	Indirectness	Imprecision	Heterogeneity	Incoherence	Confidence rating
D:P	0	No concerns	Some concerns	No concerns	Major concerns	No concerns	No concerns	Moderate
D:Q	0	No concerns	Some concerns	No concerns	Major concerns	No concerns	No concerns	Moderate
D:R	0	No concerns	Some concerns	No concerns	Some concerns	Some concerns	No concerns	Moderate
D:S	0	Some concerns	Some concerns	No concerns	Major concerns	No concerns	No concerns	Low
E:F	0	No concerns	Low risk	No concerns	Major concerns	No concerns	No concerns	Moderate
E:G	0	No concerns	Low risk	No concerns	Major concerns	No concerns	No concerns	Moderate
E:H	0	No concerns	Low risk	No concerns	Major concerns	No concerns	No concerns	Moderate
E:I	0	No concerns	Low risk	No concerns	Major concerns	No concerns	No concerns	Moderate
E:J	0	No concerns	Low risk	No concerns	Major concerns	No concerns	No concerns	Moderate
E:K	0	No concerns	Low risk	No concerns	Some concerns	Some concerns	No concerns	Moderate
E:N	0	No concerns	Some concerns	No concerns	Major concerns	No concerns	No concerns	Moderate
E:O	0	Some concerns	Some concerns	No concerns	Major concerns	No concerns	No concerns	Low
E:R	0	No concerns	Some concerns	No concerns	Major concerns	No concerns	No concerns	Moderate
E:S	0	No concerns	Some concerns	No concerns	Major concerns	No concerns	No concerns	Moderate
F:H	0	Some concerns	Some concerns	No concerns	Major concerns	No concerns	No concerns	Low
F:I	0	No concerns	Some concerns	No concerns	Some concerns	Some concerns	No concerns	Moderate
F:J	0	Some concerns	Some concerns	No concerns	Major concerns	No concerns	No concerns	Low
F:K	0	Some concerns	Some concerns	No concerns	No concerns	No concerns	No concerns	Moderate
F:L	0	Some concerns	Some concerns	No concerns	No concerns	Major concerns	No concerns	Low
F:M	0	No concerns	High risk	No concerns	Some concerns	Some concerns	No concerns	Low
F:N	0	Some concerns	Some concerns	No concerns	No concerns	No concerns	No concerns	Moderate
F:O	0	Some concerns	Some concerns	No concerns	Major concerns	No concerns	No concerns	Low
F:P	0	No concerns	High risk	No concerns	Major concerns	No concerns	No concerns	Low
F:Q	0	Some concerns	Some concerns	No concerns	Major concerns	No concerns	No concerns	Low
F:R	0	Some concerns	Some concerns	No concerns	Major concerns	No concerns	No concerns	Low
G:H	0	No concerns	Some concerns	No concerns	Major concerns	No concerns	No concerns	Moderate
G:I	0	No concerns	Some concerns	No concerns	Major concerns	No concerns	No concerns	Moderate
G:K	0	Some concerns	Some concerns	No concerns	No concerns	No concerns	No concerns	Moderate

Compariso n	χ	Within-study bias	Reporting bias	Indirectness	Imprecision	Heterogeneity	Incoherence	Confidence rating
G:L	0	No concerns	Some concerns	No concerns	No concerns	Major concerns	No concerns	Moderate
G:M	0	No concerns	Some concerns	No concerns	Major concerns	No concerns	No concerns	Moderate
G:N	0	Some concerns	Some concerns	No concerns	No concerns	Some concerns	No concerns	Moderate
G:O	0	Some concerns	Some concerns	No concerns	Major concerns	No concerns	No concerns	Low
G:P	0	No concerns	Some concerns	No concerns	Major concerns	No concerns	No concerns	High
H:I	0	No concerns	Some concerns	No concerns	Some concerns	Some concerns	No concerns	Moderate
H:J	0	Some concerns	Some concerns	No concerns	Major concerns	No concerns	No concerns	Low
H:K	0	No concerns	Some concerns	No concerns	No concerns	No concerns	No concerns	Moderate
H:L	0	No concerns	Some concerns	No concerns	No concerns	Major concerns	No concerns	Moderate
H:M	0	No concerns	Some concerns	No concerns	Some concerns	Some concerns	No concerns	Moderate
H:N	0	Some concerns	Some concerns	No concerns	No concerns	Some concerns	No concerns	Moderate
H:O	0	No concerns	Some concerns	No concerns	Major concerns	No concerns	No concerns	Moderate
H:P	0	No concerns	Some concerns	No concerns	Major concerns	No concerns	No concerns	Moderate
H:R	0	No concerns	Some concerns	No concerns	Major concerns	No concerns	No concerns	Moderate
I:J	0	No concerns	Low risk	No concerns	Major concerns	No concerns	No concerns	Moderate
I:K	0	No concerns	Low risk	No concerns	Major concerns	No concerns	No concerns	Moderate
I:L	0	No concerns	Low risk	No concerns	Major concerns	No concerns	No concerns	Moderate
I:M	0	No concerns	Some concerns	No concerns	Major concerns	No concerns	No concerns	Moderate
I:N	0	No concerns	Some concerns	No concerns	Major concerns	No concerns	No concerns	Moderate
I:O	0	No concerns	Low risk	No concerns	Major concerns	No concerns	No concerns	Moderate
I:P	0	No concerns	Some concerns	No concerns	Major concerns	No concerns	No concerns	Moderate
I:R	0	No concerns	Some concerns	No concerns	Major concerns	No concerns	No concerns	Moderate
I:S	0	No concerns	Some concerns	No concerns	Some concerns	Some concerns	No concerns	Moderate
J:K	0	Some concerns	Low risk	No concerns	No concerns	Major concerns	No concerns	Moderate
J:L	0	Some concerns	Low risk	No concerns	Major concerns	No concerns	No concerns	Moderate
J:M	0	No concerns	Some concerns	No concerns	Major concerns	No concerns	No concerns	Moderate
J:N	0	Some concerns	Low risk	No concerns	No concerns	Major concerns	No concerns	Moderate
J:O	0	Some concerns	Low risk	No concerns	Major concerns	No concerns	No concerns	High

Comparison	χ	Within-study bias	Reporting bias	Indirectness	Imprecision	Heterogeneity	Incoherence	Confidence rating
J:P	0	No concerns	Some concerns	No concerns	Major concerns	No concerns	No concerns	Moderate
J:R	0	No concerns	Some concerns	No concerns	Major concerns	No concerns	No concerns	Moderate
J:S	0	Some concerns	Some concerns	No concerns	Major concerns	No concerns	No concerns	Low
K:L	0	No concerns	Low risk	No concerns	Major concerns	No concerns	No concerns	Moderate
K:M	0	No concerns	Some concerns	No concerns	Major concerns	No concerns	No concerns	Moderate
K:O	0	Some concerns	Low risk	No concerns	Major concerns	No concerns	No concerns	Moderate
K:P	0	No concerns	Some concerns	No concerns	Some concerns	Some concerns	No concerns	Moderate
K:Q	0	No concerns	Some concerns	No concerns	No concerns	Some concerns	No concerns	Moderate
K:R	0	No concerns	Some concerns	No concerns	No concerns	Major concerns	No concerns	Moderate
K:S	0	Some concerns	Some concerns	No concerns	No concerns	No concerns	No concerns	Moderate
L:M	0	No concerns	Some concerns	No concerns	Major concerns	No concerns	No concerns	Moderate
L:N	0	Some concerns	Low risk	No concerns	Major concerns	No concerns	No concerns	Moderate
L:P	0	No concerns	Some concerns	No concerns	Some concerns	Some concerns	No concerns	Moderate
L:Q	0	No concerns	Some concerns	No concerns	No concerns	Major concerns	No concerns	Moderate
L:R	0	No concerns	Some concerns	No concerns	Some concerns	Some concerns	No concerns	Moderate
L:S	0	No concerns	Some concerns	No concerns	No concerns	Some concerns	No concerns	Moderate
M:N	0	No concerns	Some concerns	No concerns	Major concerns	No concerns	No concerns	Moderate
M:O	0	Some concerns	Some concerns	No concerns	Major concerns	No concerns	No concerns	Low
M:R	0	No concerns	Some concerns	No concerns	Major concerns	No concerns	No concerns	Moderate
M:S	0	No concerns	High risk	No concerns	Some concerns	Some concerns	No concerns	Low
N:O	0	Some concerns	Low risk	No concerns	Major concerns	No concerns	No concerns	Moderate
N:P	0	No concerns	Some concerns	No concerns	Some concerns	Some concerns	No concerns	Moderate
N:Q	0	Some concerns	Some concerns	No concerns	No concerns	Some concerns	No concerns	Moderate
N:R	0	No concerns	Some concerns	No concerns	No concerns	Major concerns	No concerns	Moderate
N:S	0	Some concerns	Some concerns	No concerns	No concerns	No concerns	No concerns	Moderate
O:P	0	Some concerns	Some concerns	No concerns	Major concerns	No concerns	No concerns	Low
O:Q	0	Some concerns	Some concerns	No concerns	Major concerns	No concerns	No concerns	Low
O:R	0	No concerns	Some concerns	No concerns	Major concerns	No concerns	No concerns	Moderate

Compariso n	χ	Within-study bias	Reporting bias	Indirectness	Imprecision	Heterogeneity	Incoherence	Confidence rating
O:S	0	Some concerns	Some concerns	No concerns	Major concerns	No concerns	No concerns	Low
P:R	0	No concerns	Some concerns	No concerns	Major concerns	No concerns	No concerns	Moderate
P:S	0	No concerns	High risk	No concerns	Major concerns	No concerns	No concerns	Low
Q:S	0	Some concerns	Some concerns	No concerns	Major concerns	No concerns	No concerns	Low
R:S	0	No concerns	Some concerns	No concerns	Major concerns	No concerns	No concerns	Moderate

A: CGM (curcumin derivatives); B: Celecoxib; C: D-002 (inhibitor of both cyclooxygenase and 5-lipoxygenase activities); D: Diacerein; E: G (glucosamine); F: G+CGM (glucosamine+curcumin derivatives); G: G+CS (glucosamine+chondroitin sulfate); H: G+CS+HA (glucosamine+chondroitin sulfate+hyaluronic acid); I: G+CS+MA (glucosamine+chondroitin sulfate+manganese ascorbate); J: G+CS+MSM (glucosamine+chondroitin sulfate+methylsulfonylmethane); K: G+Celecoxib (glucosamine+celecoxib); L: G+Ibuprofen (glucosamine+ibuprofen); M: G+MSM (glucosamine+curcumin derivatives); N: G+O+Celecoxib (glucosamine+ozone+celecoxib); O: Ibuprofen; P: MSM (methylsulfonylmethane); Q (reference): Placebo; R: UC-II (undenatured type II collagen).

Table S16. Pairwise and network meta-analysis results for short-term pain.

**Placebo (Ref)**	-0.56 (-0.95, -0.18)	0.66 (0.14, 1.18)			1.26 (0.72, 1.80)			0.31 (-0.08, 0.71)	1.38 (0.87, 1.90)	-0.23 (-1.11, 0.66)	0.02 (-0.19, 0.23)	0.43 (-0.08, 0.94)	0.77 (0.40, 1.14)				1.17 (0.65, 1.70)
0.59 (-0.44, 1.62)	**UC-II**										-0.41 (-0.72, -0.10)						
0.56 (-0.79, 1.90)	-0.04 (-1.71, 1.64)	**MSM**			0.60 (0.08, 1.11)								0.14 (-0.37, 0.65)				
0.22 (-2.07, 2.51)	-0.37 (-2.86, 2.12)	-0.33 (-2.80, 2.14)	**Ibuprofen**			2.10 (1.46, 2.74)											
**3.26 (0.96, 5.57)**	2.67 (0.26, 5.08)	2.71 (0.06, 5.36)	3.04 (-0.18, 6.26)	**G+O+Celecoxib**			-0.31 (-0.77, 0.14)										
1.15 (-0.20, 2.50)	0.56 (-1.12, 2.24)	0.60 (-0.85, 2.04)	0.93 (-1.54, 3.40)	-2.11 (-4.76,0.54)	**G+MSM**								-0.46 (-0.96, 0.05)				
**2.33 (0.59, 4.07)**	1.74 (-0.26, 3.73)	1.77 (-0.20, 3.74)	**2.10 (0.61, 3.60)**	-0.94 (-3.79,1.92)	1.18 (-0.79, 3.14)	**G+Ibuprofen**							-1.73 (-2.25, -1.22)				
**2.95 (1.14, 4.76)**	**2.36 (0.42, 4.30)**	2.40 (0.16, 4.63)	2.73 (-0.16, 5.62)	-0.31 (-1.74,1.11)	1.80 (-0.44, 4.04)	0.62 (-1.85,3.10)	**G+Celecoxib**									-2.55 (-4.75, -0.36)	

0.51 (-0.75, 1.76)	-0.09 (-1.63, 1.45)	-0.05 (-1.88, 1.78)	0.28 (-2.31, 2.88)	-2.76 (-5.31, -0.21)	-0.65 (-2.48, 1.19)	-1.82 (-3.95,0.30)	**-2.45 (-4.56, -0.33)**	**G+CS +MSM**			-0.49 (-0.89, -0.09)						
1.38 (-0.06, 2.83)	0.79 (-0.98, 2.57)	0.83 (-1.14, 2.80)	1.16 (-1.55, 3.87)	-1.88 (-4.60,0.84)	0.23 (-1.74, 2.21)	-0.94 (-3.20,1.32)	-1.57 (-3.88,0.75)	0.88 (-1.04,2.80)	**G+CS +MA**								
-0.22 (-1.24, 0.80)	-0.81 (-2.26, 0.64)	-0.78 (-2.46, 0.91)	-0.44 (-2.95, 2.06)	**-3.48 (-6.00, -0.97)**	-1.37 (-3.06, 0.32)	**-2.55 (-4.56, -0.53)**	**-3.17 (-5.25, -1.10)**	-0.73 (-2.34,0.89)	-1.61 (-3.37,0.16)	**G+CS+HA**							
0.21 (-0.42, 0.84)	-0.38 (-1.33, 0.57)	-0.35 (-1.80, 1.11)	-0.01 (-2.35, 2.33)	**-3.05 (-5.27, -0.84)**	-0.94 (-2.40, 0.52)	-2.12 (-3.92, -0.31)	**-2.74 (-4.44, -1.04)**	-0.30 (-1.55,0.96)	-1.17 (-2.75,0.40)	0.43 (-0.77,1.63)	**G+CS**	-1.51 (-2.01, -1.01)		-1.11 (-1.66, -0.56)	0.53 (0.01, 1.04)	0.23 (0.06, 0.41)	1.15 (0.65, 4.65)
-0.31 (-1.34, 0.71)	-0.91 (-2.25, 0.44)	-0.87 (-2.47, 0.73)	-0.54 (-2.93, 1.86)	**-3.58 (-6.01, -1.15)**	-1.47 (-3.07, 0.14)	**-2.64 (-4.51, -0.77)**	**-3.27 (-5.23, -1.30)**	-0.82 (-2.37,0.73)	-1.70 (-3.47,0.07)	-0.09 (-1.54,1.35)	-0.52 (-1.52, 0.47)	**G+CGM**	0.31 (-0.20, 0.82)				0.75 (0.23, 1.26)
0.59 (-0.38, 1.57)	0.00 (-1.37, 1.38)	0.04 (-1.30, 1.38)	0.37 (-1.70, 2.45)	-2.67 (-5.13, -0.21)	-0.56 (-1.90, 0.78)	-1.73 (-3.17, -0.29)	**-2.36 (-4.37, -0.35)**	0.09 (-1.47,1.65)	-0.79 (-2.53,0.95)	0.81 (-0.59,2.22)	0.38 (-0.70, 1.46)	0.91 (-0.29, 2.10)	**G**				0.43 (-0.08, 0.94)
-0.90 (-2.49, 0.69)	-1.49 (-3.23, 0.25)	-1.46 (-3.51, 0.60)	-1.12 (-3.88, 1.63)	**-4.16 (-6.81, -1.52)**	-2.05 (-4.11, 0.01)	**-3.23 (-5.54, -0.91)**	**-3.85 (-6.09, -1.62)**	-1.41 (-3.33,0.52)	-2.29 (-4.43, -0.14)	-0.68 (-2.56,1.20)	-1.11 (-2.57, 0.34)	-0.59 (-2.35, 1.18)	-1.49 (-3.31, 0.32)	**Diacerein**			
0.74 (-0.84, 2.31)	0.15 (-1.58, 1.87)	0.18 (-1.87, 2.23)	0.51 (-2.23, 3.26)	-2.53 (-5.17,0.12)	-0.41 (-2.47, 1.64)	-1.59 (-3.90,0.72)	-2.21 (-4.44,0.01)	0.23 (-1.68,2.15)	-0.65 (-2.79,1.49)	0.96 (-0.92,2.83)	0.53 (-0.92, 1.97)	1.05 (-0.70, 2.81)	0.14 (-1.66, 1.95)	1.64 (-0.41, 3.69)	**D-002**		

0.44 (-1.06, 1.94)	-0.15 (-1.81, 1.51)	-0.11 (-2.10, 1.87)	0.22 (-2.49, 2.92)	-2.82 (-4.57, -1.08)	-0.71 (-2.70, 1.28)	-1.89 (-4.14,0.37)	**-2.51 (-3.53, -1.49)**	-0.06 (-1.92,1.79)	-0.94 (-3.03,1.14)	0.66 (-1.15,2.47)	0.23 (-1.13, 1.59)	0.76 (-0.93, 2.44)	-0.15 (-1.89, 1.58)	1.34 (-0.65, 3.33)	-0.30 (-2.28, 1.69)	**Celecoxib**	
1.07 (0.05, 2.10)	0.48 (-0.86, 1.82)	0.52 (-1.09, 2.12)	0.85 (-1.55, 3.24)	-2.19 (-4.62,0.24)	-0.08 (-1.68, 1.53)	-1.25 (-3.13,0.62)	-1.88 (-3.85,0.09)	0.57 (-0.99,2.12)	-0.31 (-2.08,1.46)	1.29 (-0.15,2.74)	0.86 (-0.14, 1.86)	1.39 (0.21, 2.56)	0.48 (-0.72, 1.67)	1.97 (0.21, 3.74)	0.33 (-1.42, 2.09)	0.63 (-1.06, 2.32)	**CGM**

UC-II, undenatured type II collagen; MSM, methylsulfonylmethane; G+O+Celecoxib, glucosamine+ozone+celecoxib; G+MSM, glucosamine+methylsulfonylmethane; G+Ibuprofen, glucosamine+ibuprofen; G+Celecoxib, glucosamine+celecoxib; G+CS+MSM, glucosamine+chondroitin sulfate+methylsulfonylmethane; G+CS+MA, glucosamine+chondroitin sulfate+manganese ascorbate; G+CS+HA, glucosamine+chondroitin sulfate+hyaluronic acid; G+CS; glucosamine+chondroitin sulfate; G, glucosamine; D-002, inhibitor of both cyclooxygenase and 5-lipoxygenase activities; D-002, inhibitor of both cyclooxygenase and 5-lipoxygenase activities; CGM, curcumin derivatives.

Table S17. SUCRA and mean rank results for short-term pain.

Treatment	SUCRA	PrBest	MeanRank
CGM	24.6	0	13.8
Celecoxib	65.2	0.2	6.9
D-002	42.9	0	10.7
Diacerein	52.9	0.5	9
G	8.1	0	16.6
G+CGM	48.6	0	9.7
G+CS	16.5	0	15.2
G+CS+HA	33.7	0	12.3
G+CS+MA	19.6	0	14.7
G+CS+MSM	70.3	2.1	6.1
G+Celecoxib	45.3	0.1	10.3
G+Ibuprofen	92.6	22	2.3
G+MSM	87.2	19.6	3.2
G+O+Celecoxib	65.6	0.9	6.9
Ibuprofen	94.6	54.5	1.9
MSM	37	0.1	11.7
Placebo	46.1	0.1	10.2
UC-II	49.2	0	9.6

UC-II, undenatured type II collagen; MSM, methylsulfonylmethane; G+O+Celecoxib, glucosamine+ozone+celecoxib; G+MSM, glucosamine+methylsulfonylmethane; G+Ibuprofen, glucosamine+ibuprofen; G+Celecoxib, glucosamine+celecoxib; G+CS+MSM, glucosamine+chondroitin sulfate+methylsulfonylmethane; G+CS+MA, glucosamine+chondroitin sulfate+manganese ascorbate; G+CS+HA, glucosamine+chondroitin sulfate+hyaluronic acid; G+CS; glucosamine+chondroitin sulfate; G, glucosamine; D-002, inhibitor of both cyclooxygenase and 5-lipoxygenase activities; D-002, inhibitor of both cyclooxygenase and 5-lipoxygenase activities; CGM, curcumin derivatives

Table S18. Studies excluded from the network meta-analysis for short-term pain.

Study	Treatment nodes	Pain [MD (95%CI)]	Pain [SMD (95%CI)
¶Kongtharvonskul, 2016	G+Diacerein vs G+Placebo	0.75 (-2.23, 3.73)	0.08 (-0.25, 0.41)
¶Kumar, 2020	G+Oxaceprol vs Oxaceprol	-1.70 (-5.64, 2.24)	-0.26 (-0.89, 0.36)
¶Sun, 2020	G+Etoricoxib vs Etoricoxib	-3.63 (-4.05, -3.21)	-3.03 (-3.60, -2.46)
¶Zhijun, 2019	G+Meloxicam vs Meloxicam	-1.31 (-1.76, -0.86)	-1.21 (-1.66, -0.75)
†Arti, 2012	G+alendronate vs G	1, 3, 6 and 12 weeks: $p >$ 0.05	NA
†Leffler, 1999	G+CS+MA vs Placebo	8 weeks: $p < 0.05$	NA

¶Disconnected studies from the network; † study excluded due to intransitivity

MD: Mean difference; CI: confidence interval; SMD: standardized mean difference

Table S19. Confidence rating for long-term pain using CINeMA.

Comparison	χ	Within-study bias	Reporting bias	Indirectness	Imprecision	Heterogeneity	Incoherence	Confidence rating
A:B	1	No concerns	Some concerns	No concerns	No concerns	Some concerns	No concerns	Moderate
A:D	2	No concerns	Some concerns	No concerns	No concerns	No concerns	No concerns	Moderate
A:E	2	No concerns	Some concerns	No concerns	No concerns	No concerns	No concerns	Moderate
A:H	2	No concerns	Some concerns	No concerns	No concerns	Some concerns	No concerns	Moderate
B:D	1	No concerns	High risk	No concerns	No concerns	Some concerns	No concerns	Moderate
B:E	2	No concerns	High risk	No concerns	No concerns	Some concerns	No concerns	Moderate
B:H	1	No concerns	Some concerns	No concerns	No concerns	Some concerns	No concerns	Moderate
C:E	1	No concerns	Low risk	No concerns	No concerns	No concerns	No concerns	High
D:E	2	No concerns	Some concerns	No concerns	No concerns	No concerns	No concerns	Moderate
D:G	1	No concerns	High risk	No concerns	Some concerns	No concerns	No concerns	Moderate
D:H	2	No concerns	Some concerns	No concerns	No concerns	Some concerns	No concerns	Moderate
E:H	5	No concerns	Some concerns	No concerns	No concerns	No concerns	No concerns	Moderate
E:I	1	No concerns	Some concerns	No concerns	No concerns	No concerns	No concerns	Moderate
F:H	1	No concerns	Low risk	No concerns	No concerns	No concerns	No concerns	High
H:I	1	No concerns	Some concerns	No concerns	No concerns	No concerns	No concerns	Moderate
A:C	0	No concerns	Some concerns	No concerns	No concerns	No concerns	No concerns	Moderate
A:F	0	No concerns	Some concerns	No concerns	No concerns	No concerns	No concerns	Moderate
A:G	0	No concerns	Some concerns	No concerns	Some concerns	No concerns	No concerns	Moderate
A:I	0	No concerns	Some concerns	No concerns	No concerns	No concerns	No concerns	Moderate
B:C	0	No concerns	Some concerns	No concerns	No concerns	No concerns	No concerns	Moderate
B:F	0	No concerns	Some concerns	No concerns	No concerns	No concerns	No concerns	Moderate
B:G	0	No concerns	High risk	No concerns	Some concerns	Some concerns	No concerns	Low
B:I	0	No concerns	High risk	No concerns	No concerns	Some concerns	No concerns	Moderate
C:D	0	No concerns	Some concerns	No concerns	No concerns	No concerns	No concerns	Moderate
C:F	0	No concerns	Low risk	No concerns	No concerns	No concerns	No concerns	High
C:G	0	No concerns	Some concerns	No concerns	No concerns	No concerns	No concerns	Moderate

Comparison	χ	Within-study bias	Reporting bias	Indirectness	Imprecision	Heterogeneity	Incoherence	Confidence rating
C:H	0	No concerns	Some concerns	No concerns	No concerns	No concerns	No concerns	Moderate
C:I	0	No concerns	Some concerns	No concerns	No concerns	No concerns	No concerns	Moderate
D:F	0	No concerns	Some concerns	No concerns	No concerns	No concerns	No concerns	Moderate
D:I	0	No concerns	Some concerns	No concerns	No concerns	No concerns	No concerns	Moderate
E:F	0	No concerns	Some concerns	No concerns	No concerns	No concerns	No concerns	Moderate
E:G	0	No concerns	Some concerns	No concerns	Some concerns	No concerns	No concerns	Moderate
F:G	0	No concerns	Some concerns	No concerns	No concerns	No concerns	No concerns	Moderate
F:I	0	No concerns	Some concerns	No concerns	No concerns	No concerns	No concerns	Moderate
G:H	0	No concerns	Some concerns	No concerns	Some concerns	No concerns	No concerns	Moderate
G:I	0	No concerns	High risk	No concerns	Some concerns	Some concerns	No concerns	Low

A: CS (chondroitin sulfate); B: Celecoxib; C: Diacerein; D: G (glucosamine); E: G+CS (glucosamine+chondroitin sulfate); F: G+CS+MA (glucosamine+chondroitin sulfate+manganese ascorbate); G: G+omega-3 (glucosamine+omega-3); H (reference): Placebo; I: UC-II (undenatured type II collagen).

Table S20. Pairwise and network meta-analysis results for long-term pain.

**Placebo (Ref)**	-0.72 (-1.11, -0.34)		2.64 (2.00, 3.28)	-0.03 (-0.26, 0.20)	0.02 (-0.11, 0.15)		0.14 (-0.02, 0.29)	0.01 (-0.11, 0.14)
0.61 (0.21,1.02)	**UC-II**			-0.52 (-0.90, -0.15)				
0.24 (-0.19,0.67)	-0.37 (-0.95,0.20)	**G+omega-3**			-0.26 (-0.56, 0.04)			
**2.64 (1.95,3.32)**	**2.02 (1.23,2.82)**	**2.40 (1.59,3.21)**	**G+CS+MA**					
-0.01 (-0.18,0.16)	-0.63 (-1.03, -0.22)	-0.25 (-0.68,0.18)	**-2.65 (-3.35, -1.94)**	**G+CS**	-0.05 (-0.19, 0.10)	-2.89 (-3.63, -2.15)	0.01 (-0.10, 0.13)	-0.04 (-0.24, 0.16)
-0.02 (-0.23,0.18)	-0.64 (-1.07, -0.20)	-0.26 (-0.64,0.12)	**-2.66 (-3.37, -1.94)**	-0.01 (-0.21,0.19)	**G**		0.12 (-0.03, 0.28)	-0.01 (-0.13, 0.12)
**-2.90 (-3.70, -2.11)**	**-3.52 (-4.39, -2.64)**	**-3.14 (-4.03, -2.25)**	**-5.54 (-6.59, -4.49)**	**-2.89 (-3.67, -2.11)**	**-2.88 (-3.69, -2.08)**	**Diacerein**		
0.06 (-0.16,0.28)	-0.56 (-0.99, -0.12)	-0.18 (-0.63,0.27)	**-2.58 (-3.30, -1.86)**	0.07 (-0.12,0.26)	0.08 (-0.16,0.31)	**2.96 (2.16,3.76)**	**Celecoxib**	-0.14 (-0.30, 0.01)
-0.02 (-0.23,0.18)	-0.64 (-1.07, -0.20)	-0.26 (-0.70,0.18)	**-2.66 (-3.37, -1.94)**	-0.01 (-0.21,0.19)	-0.00 (-0.22,0.22)	**2.88 (2.08,3.69)**	-0.08 (-0.32,0.16)	**CS**

UC-II, undenatured type II collagen; G+omega-3, glucosamine+omega-3; G+CS+MA, glucosamine+chondroitin sulfate+manganese ascorbate; G+CS; glucosamine+chondroitin sulfate; CS, chondroitin sulfate.

Table S21. SUCRA and mean rank results for long-term pain.

Treatment	SUCRA	PrBest	MeanRank
CS	39.5	0	5.8
Celecoxib	34.4	0	6.2
Diacerein	52	0	4.8
G	0	0	9
G+CS	34.2	0	6.3
G+CS+MA	36.3	0	6.1
G+omega-3	100	100	1
Placebo	67.5	0	3.6
UC-II	86.1	0	2.1

UC-II, undenatured type II collagen; G+omega-3, glucosamine+omega-3; G+CS+MA, glucosamine+chondroitin sulfate+manganese ascorbate; G+CS; glucosamine+chondroitin sulfate; CS, chondroitin sulfate

Table S22. Studies excluded from the network meta-analysis for long-term pain.

Study	Treatment nodes	Pain [MD (95%CI)]	Pain [SMD (95%CI)]
⁋Kongtharvonskul, 2016	G+Diacerein vs G+Placebo	0.26 (-2.24, 2.76)	0.04 (-0.31, 0.38)

⁋Disconnected studies from the network

MD: Mean difference; CI: confidence interval; SMD: standardized mean difference

Table S23. Pairwise and network meta-analysis results for adverse events.

Results in the league tables below are presented as odds ratio (OR), with the corresponding 95% confidence interval (CI). Lower left triangle presents the network meta-analysis results and upper right triangle presents pairwise meta-analysis results.

Ref (Placebo)	1.06 (0.36, 3.13)			1.13 (0.39, 3.23)	0.79 (0.30, 2.05)	0.82 (0.42, 1.62)	0.46 (0.08, 2.75)	0.88 (0.46, 1.66)			1.62 (0.62, 4.23)	0.54 (0.29, 0.98)	1.00 (0.13, 7.60)
1.00 (0.35,2.85)	UC-II					0.79 (0.13, 4.84)							
0.54 (0.11,2.56)	0.54 (0.09,3.30)	G+omega-3						1.33 (0.53, 3.34)					
3.15 (0.76,13.04)	3.14 (0.60,16.55)	5.86 (0.78,43.85)	G+Celecoxib								0.34 (0.15, 0.78)		
1.13 (0.27,4.70)	1.12 (0.19,6.58)	2.09 (0.25,17.34)	0.36 (0.05,2.67)	G+CS+MA									
0.79 (0.20,3.07)	0.79 (0.14,4.36)	1.46 (0.18,11.61)	0.25 (0.03,1.79)	0.70 (0.10,5.04)	G+CS+HA								
0.86 (0.48,1.52)	0.85 (0.32,2.25)	1.59 (0.33,7.67)	0.27 (0.07,1.06)	0.76 (0.16,3.55)	1.09 (0.25,4.75)	G+CS		0.89 (0.34, 2.36)	1.52 (0.42, 5.47)	2.07 (0.18, 24.15)	1.16 (0.69, 1.97)	0.51 (0.27, 0.94)	0.95 (0.02, 49.00)
0.71 (0.14,3.65)	0.70 (0.11,4.72)	1.31 (0.17,10.45)	0.22 (0.03,1.85)	0.63 (0.07,5.54)	0.90 (0.11,7.56)	0.83 (0.15,4.42)	G+CGM	0.77 (0.19, 3.20)					2.15 (0.36,

													12.76)
0.72 (0.32,1.61)	0.72 (0.21,2.45)	1.33 (0.35,5.06)	0.23 (0.05,1.03)	0.64 (0.12,3.30)	0.91 (0.19,4.44)	0.84 (0.36,1.93)	1.02 (0.21,4.97)	G			1.30 (0.48, 3.53)	0.53 (0.29, 0.98)	2.80 (0.50, 15.73)
1.30 (0.24,7.15)	1.30 (0.20,8.46)	2.42 (0.26,22.88)	0.41 (0.05,3.39)	1.16 (0.13,10.69)	1.65 (0.19,14.61)	1.52 (0.31,7.56)	1.84 (0.18,18.79)	1.82 (0.30,11.06)	Diacerein				
1.77 (0.12,26.41)	1.77 (0.11,29.42)	3.30 (0.15,71.16)	0.56 (0.03,10.97)	1.58 (0.07,33.47)	2.25 (0.11,46.29)	2.07 (0.15,29.00)	2.51 (0.11,57.28)	2.47 (0.16,39.36)	1.36 (0.06,29.86)	D-002			
1.06 (0.42,2.65)	1.05 (0.30,3.71)	1.96 (0.36,10.71)	0.34 (0.11,0.99)	0.94 (0.17,5.14)	1.34 (0.26,6.92)	1.23 (0.54,2.82)	1.50 (0.24,9.13)	1.47 (0.52,4.20)	0.81 (0.13,4.93)	0.60 (0.04,9.47)	Celecoxib	0.34 (0.14, 0.80)	
0.43 (0.19,0.98)	0.43 (0.13,1.48)	0.80 (0.16,4.00)	0.14 (0.03,0.61)	0.38 (0.07,2.00)	0.55 (0.11,2.69)	0.51 (0.22,1.15)	0.61 (0.11,3.48)	0.60 (0.25,1.47)	0.33 (0.05,2.01)	0.24 (0.02,3.87)	0.41 (0.15,1.14)	CS	
1.40 (0.24,8.06)	1.40 (0.19,10.17)	2.60 (0.29,22.96)	0.44 (0.05,3.97)	1.25 (0.13,11.93)	1.78 (0.19,16.31)	1.64 (0.28,9.59)	1.98 (0.28,13.87)	1.95 (0.35,10.91)	1.07 (0.10,11.70)	0.79 (0.03,18.92)	1.33 (0.20,8.87)	3.24 (0.51,20.41)	CGM

UC-II, undenatured type II collagen; MSM, methylsulfonylmethane; G+omega-3, glucosamine+omega-3; G+O+Celecoxib, glucosamine+ozone+celecoxib; G+MSM, glucosamine+methylsulfonylmethane; G+Ibuprofen, glucosamine+ibuprofen; G+Celecoxib, glucosamine+celecoxib; G+CS+MSM, glucosamine+chondroitin sulfate+methylsulfonylmethane; G+CS+MA, glucosamine+chondroitin sulfate+manganese ascorbate; G+CS+HA, glucosamine+chondroitin sulfate+hyaluronic acid; G+CS; glucosamine+chondroitin sulfate; G, glucosamine; D-002, inhibitor of both cyclooxygenase and 5-lipoxygenase activities; D-002, inhibitor of both cyclooxygenase and 5-lipoxygenase activities; CGM, curcumin derivatives.

Table S24. Rank results for adverse events.

Treatment	SUCRA	PrBest	MeanRank
CGM	54.1	0.1	7
CS	64.1	11.6	5.7
Celecoxib	13.6	0	12.2
D-002	56	0.1	6.7
Diacerein	65.7	27.3	5.5
G	61.2	9.1	6
G+CGM	35.5	0.1	9.4
G+CS	38.5	1.8	9
G+CS+HA	43.8	0	8.3
G+CS+MA	41.9	2.2	8.6
G+Celecoxib	56.2	5.6	6.7
G+omega-3	89.3	40.3	2.4
Placebo	27.4	0.6	10.4
UC-II	52.6	1.3	7.2

UC-II, undenatured type II collagen; MSM, methylsulfonylmethane; G+omega-3, glucosamine+omega-3; G+O+Celecoxib, glucosamine+ozone+celecoxib; G+MSM, glucosamine+methylsulfonylmethane; G+Ibuprofen, glucosamine+ibuprofen; G+Celecoxib, glucosamine+celecoxib; G+CS+MSM, glucosamine+chondroitin sulfate+methylsulfonylmethane; G+CS+MA, glucosamine+chondroitin sulfate+manganese ascorbate; G+CS+HA, glucosamine+chondroitin sulfate+hyaluronic acid; G+CS; glucosamine+chondroitin sulfate; G, glucosamine; D-002, inhibitor of both cyclooxygenase and 5-lipoxygenase activities; D-002, inhibitor of both cyclooxygenase and 5-lipoxygenase activities; CGM, curcumin derivatives.

Table S25. Results of a side-splitting method for adverse events.

Side	Direct		Indirect		Difference		P>z	tau
	Coef.	Std. Err.	Coef.	Std. Err.	Coef.	Std. Err.		
Placebo UC-II	0.031767	0.769192	-0.0297	0.802609	0.061463	1.115422	0.956	0.534827
CGM Placebo	1.81E-12	1.150788	0.85033	1.428886	-0.85033	1.834674	0.643	0.502877
CGM G	1.053192	1.006098	-0.33928	1.553429	1.392473	1.768973	0.431	0.496984
CGM G+CGM	0.767255	1.035854	-0.57594	4.435943	1.343198	4.609239	0.771	0.498816
CGM G+CS	-0.05481	2.074525	0.616796	1.003834	-0.6716	2.304633	0.771	0.498816
CS Placebo	-0.62353	0.472148	-1.74846	1.019302	1.124928	1.146903	0.327	0.494778
CS Celecoxib	-1.06428	0.693523	-0.62977	0.890008	-0.4345	1.123291	0.699	0.535488
CS G	-0.59935	0.47335	1.147111	2.079125	-1.74646	2.144296	0.415	0.501654
CS G+CS	-0.72377	0.508841	-0.56582	1.013175	-0.15795	1.16381	0.892	0.54041
Celecoxib Placebo	0.480852	0.702705	-0.27724	0.627664	0.758095	0.930171	0.415	0.505393
Celecoxib G	0.233606	0.745537	0.615829	0.852166	-0.38222	1.12785	0.735	0.543933
Celecoxib G+CS	0.224732	0.477151	0.117346	1.208487	0.107386	1.281457	0.933	0.545868
Celecoxib G+Celecoxib	-1.09291	0.551915	-0.07029	135.3917	-1.02262	135.3924	0.994	0.492896
D-002 G+CS	0.728239	1.346529	0.426581	290.2139	0.301658	290.2153	0.999	0.492892
Diacerein G+CS	0.419854	0.818096	0.335539	158.1186	0.084315	158.1204	1	0.492895
G Placebo	-0.22759	0.456698	-0.98562	1.129169	0.758029	1.220655	0.535	0.514905
G G+CGM	-0.26236	0.883244	1.484351	1.978679	-1.74672	2.14438	0.415	0.501655
G G+CS	-0.11732	0.531038	-0.34468	0.799453	0.227359	0.960871	0.813	0.53547
G G+omega-3	0.287682	0.680463	-0.68377	195.5656	0.971456	195.5669	0.996	0.492894
G+CGM Placebo	-0.76726	1.038054	0.735593	1.784391	-1.50285	2.180852	0.491	0.503369
G+CS Placebo	-0.22579	0.296986	0.696621	1.018741	-0.92241	1.060715	0.385	0.476166
G+CS UC-II	-0.33795	0.505673	1.364745	1.383238	-1.7027	1.443873	0.238	0.469177
G+CS+HA Placebo	.	.	.	.	.	.	.	.
G+CS+MA Placebo	.	.	.	.	.	.	.	.

UC-II, undenatured type II collagen; MSM, methylsulfonylmethane; G+omega-3, glucosamine+omega-3; G+O+Celecoxib, glucosamine+ozone+celecoxib; G+MSM, glucosamine+methylsulfonylmethane; G+Ibuprofen, glucosamine+ibuprofen; G+Celecoxib, glucosamine+celecoxib; G+CS+MSM, glucosamine+chondroitin sulfate+methylsulfonylmethane; G+CS+MA, glucosamine+chondroitin sulfate+manganese ascorbate; G+CS+HA, glucosamine+chondroitin sulfate+hyaluronic acid; G+CS; glucosamine+chondroitin sulfate; G,

glucosamine; D-002, inhibitor of both cyclooxygenase and 5-lipoxygenase activities; D-002, inhibitor of both cyclooxygenase and 5-lipoxygenase activities; CGM, curcumin derivatives

Table S26. Results of global inconsistency tests for the primary analyses.

Time-point	Pain
Adverse events (Overall)	$Chi^2 = 4.27$, $p = 0.8931$

Table S27. The quality assessment included studies in the network meta-analysis.

Author, year	D1	D2	D3	D4	D5	D6	D7	Total	Overall
Clegg, 2006	1	1	1	1	1	1	0	6	Low
Crowley, 2009	1	1	1	1	0	1	1	6	Low
Das, 2000	1	1	1	1	1	1	0.5	6.5	Low
Feng, 2017	1	0.5	0.5	0.5	0	1	0.5	4	Some concerns
Fransen, 2015	1	1	1	1	1	1	1	7	Low
Gang, 2019	0.5	0.5	0.5	0.5	0	1	0.5	3.5	Some concerns
Gruenwald, 2009	1	1	1	1	1	1	0	6	Low
Hochberg, 2016	1	1	1	1	1	1	0	6	Low
Kalman_2017	1	1	1	1	1	1	0	6	Low
Khanna, 2020	1	0.5	0.5	0.5	0	1	0	3.5	Some concerns
Lubis, 2017	1	0.5	0.5	0.5	1	1	0	4.5	Some concerns
Lugo, 2016	1	1	1	1	1	1	0	6	Low
Madhu_2013	1	1	0.5	0	1	1	0	4.5	Some concerns
Mirunalini, 2014	1	1	1	1	0	1	0	5	Low
Puente, 2017	1	0	0	0	1	1	0	3	High
Roman-Blas, 2017	1	1	1	1	1	1	1	7	Low
Selvan, 2012	1	0.5	0.5	0.5	0	1	0	3.5	Some concerns
Shahine, 2014	1	0.5	0.5	0	0	1	0.5	3.5	Some concerns
Thomas, 2020	1	0	0	0	0	1	0	2	High
Tsuji, 2016	1	1	1	1	1	1	0	6	Low
Usha, 2004	1	1	1	1	0	1	0	5	Low
Wang, 2021a	1	1	1	1	1	1	0	6	Low
Wang, 2021b	1	1	1	1	1	1	0	6	Low
Zhang, 2021	1	0.5	0.5	0.5	0	1	0	3.5	Some concerns

Domain1: Random sequence generation; Domain2: Allocation concealment; Domain3: Blinding of participants and personnel; Domain4: Blinding of outcome assessment; Domain5: Incomplete outcome data; Domain6: Selective reporting; Domain7: Other sources of bias; yes: low risk of bias; no: high risk of bias; unclear: unclear risk of bias. Low risk of bias = 1, unclear risk of bias = 0.5, high risk of bias = 0. We assigned a point scale to the domain-level judgments: low risk ('1'), unclear risk or some concerns ('0.5'), or high risk ('0'), and studies were categorized into risk levels: high risk (0–3 points), some concerns (>3 to <5 points), and low risk (5–7 points).

Figure S1. Risk of bias graph.

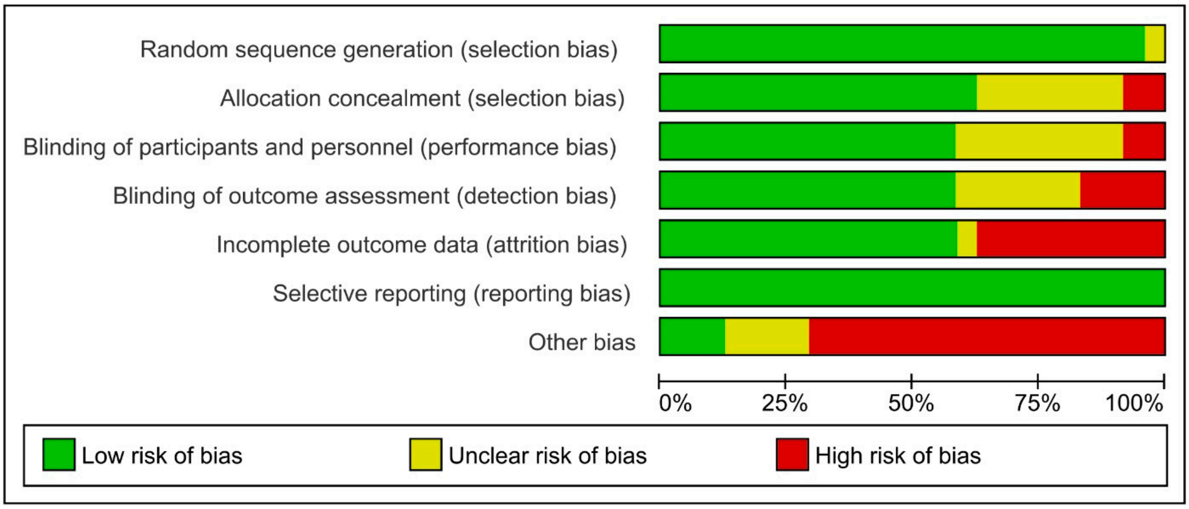

Figure S2. Risk of bias summary.

	Random sequence generation (selection bias)	Allocation concealment (selection bias)	Binding of participants and personnel (performance bias)	Binding of outcome assessment (detection bias)	Incomplete outcome data (attrition bias)	Selective reporting (reporting bias)	Other bias
Clegg, 2006	+	+	+	+	+	+	−
Crowley, 2009	+	+	+	+	−	+	+
Das, 2000	+	+	+	+	+	+	?
Feng, 2017	+	?	?	?	−	+	?
Fransen, 2015	+	+	+	+	+	+	+
Gang, 2019	?	?	?	?	−	+	?
Gruenwald, 2009	+	+	+	+	+	+	−
Hochberg, 2016	+	+	+	+	+	+	−
Kalman, 2017	+	+	+	+	+	+	−
Khanna, 2020	+	?	?	?	−	+	−
Lubis, 2017	+	?	?	?	+	+	−
Lugo, 2016	+	+	+	+	+	+	−
Madhu, 2013	+	+	?	−	+	+	−
Mirunalini, 2014	+	+	+	+	−	+	−
Puente, 2017	+	−	−	−	+	+	−
Roman-Blas, 2017	+	+	+	+	+	+	+
Selvan, 2012	+	?	?	?	−	+	−
Shahine, 2014	+	?	?	−	−	+	?
Thomas, 2020	+	−	−	−	?	+	−
Tsuji, 2016	+	+	+	+	+	+	−
Usha, 2004	+	+	+	+	−	+	−
Wang, 2021a	+	+	+	+	+	+	−
Wang, 2021b	+	+	+	+	+	+	−
Zhang, 2021	+	?	?	?	−	+	−

Assessment of transitivity

Transitivity is one of the key assumptions in network meta-analysis (NMA), which assesses whether the distribution of effect modifiers is similar for all contributing sources of direct evidence. To evaluate transitivity in the NMA, we show boxplots for the distributions of two potential effect modifiers: mean age and percentage of females across the available direct comparisons. When there are no significant differences in the distributions of effect modifiers are found, the transitivity assumption is considered to hold in the network.

Figure S3. Assessment of transitivity in terms of mean age distribution in the network of interventions for overall pain outcome.

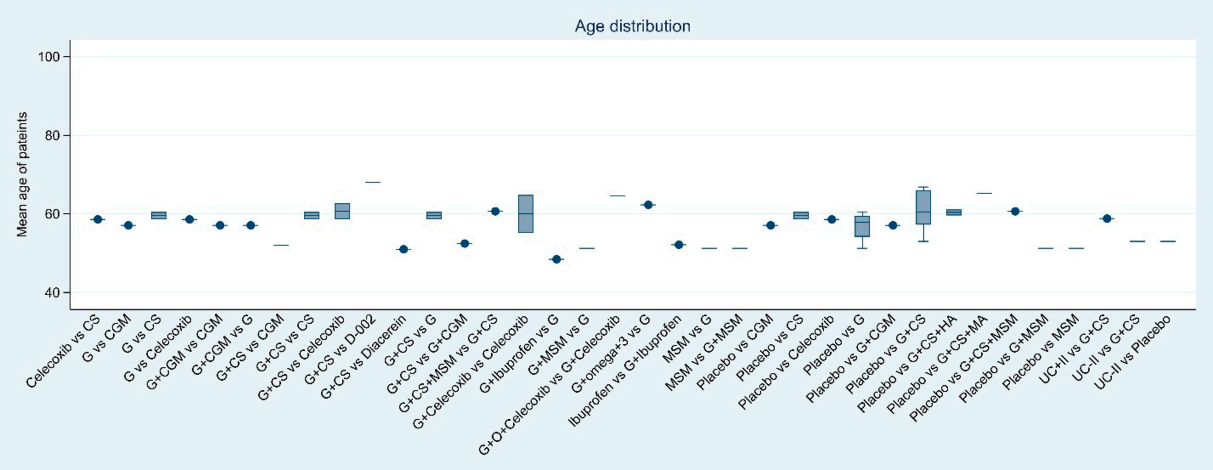

Figure S4. Assessment of transitivity in terms of percentage of females in the network of interventions for overall pain outcome.

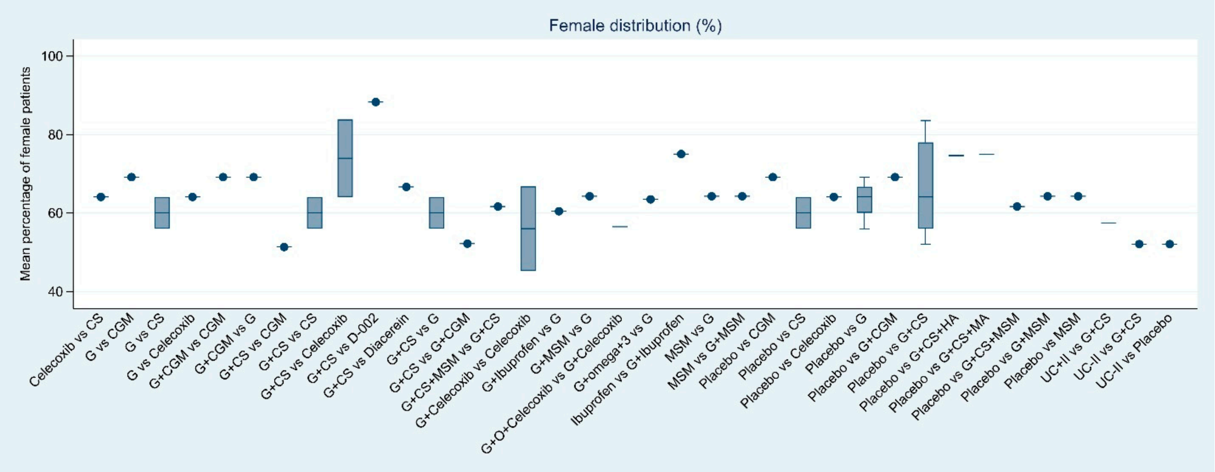

Figure S5. Assessment of transitivity in terms of mean age distribution in the network of interventions for short-term pain outcome.

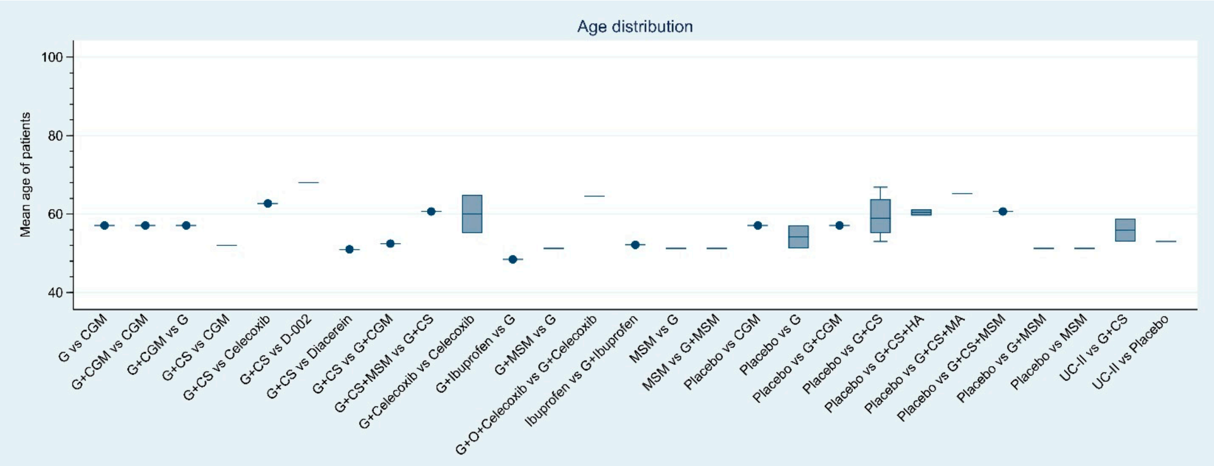

Figure S6. Assessment of transitivity in terms of percentage of females in the network of interventions for short-term pain outcome.

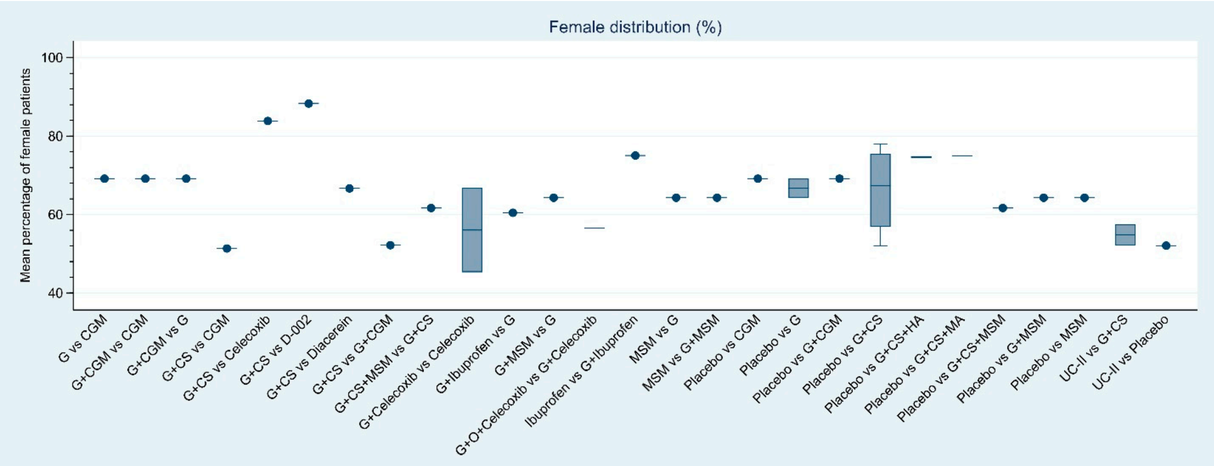

Figure S7. Assessment of transitivity in terms of mean age distribution in the network of interventions for long-term pain outcome.

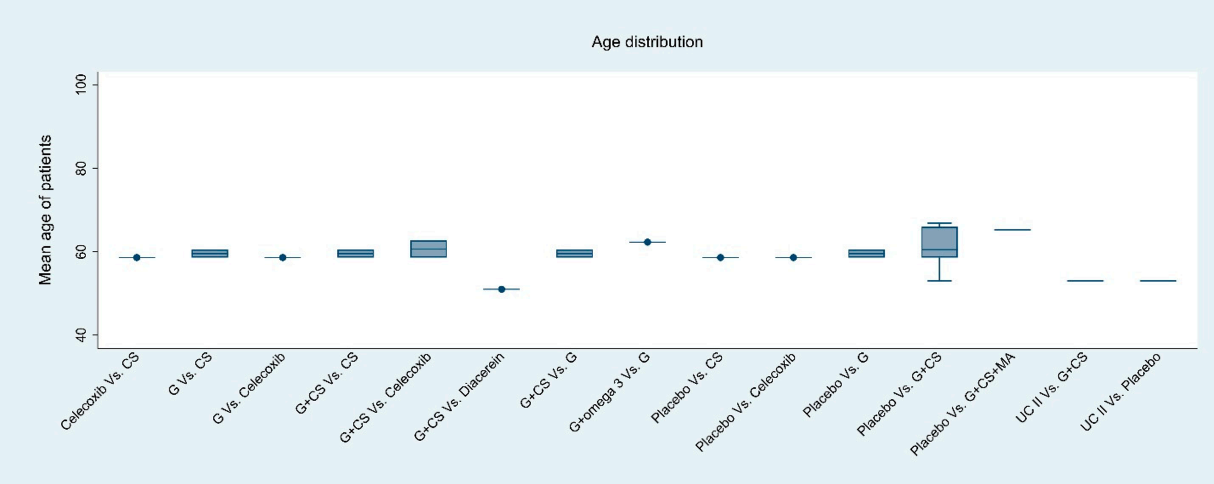

Figure S8. Assessment of transitivity in terms of percentage of females in the network of interventions for long-term pain outcome.

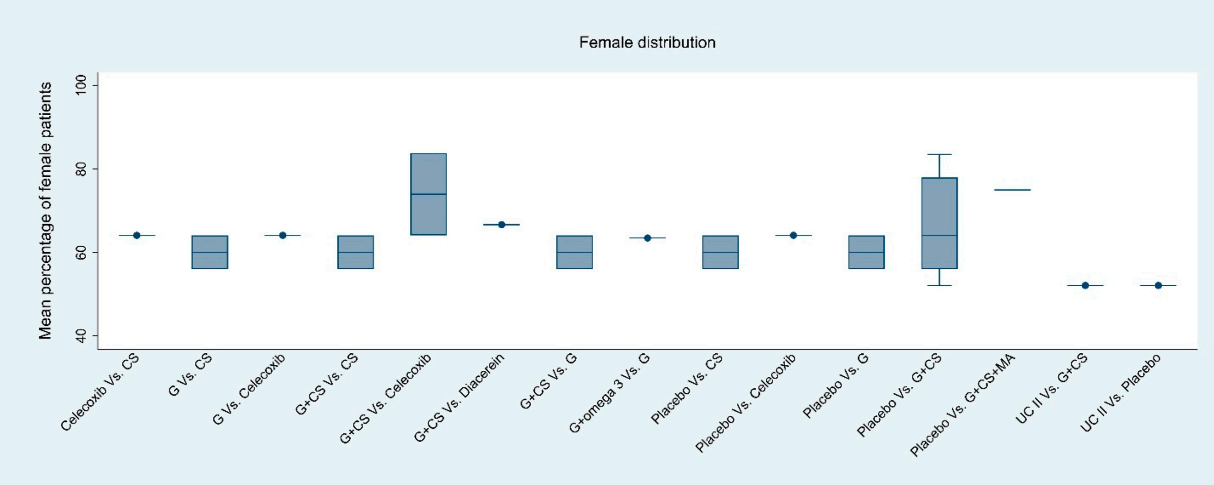

Assessment of incoherence

Figure S9. Inconsistency factors for overall pain of all trials.

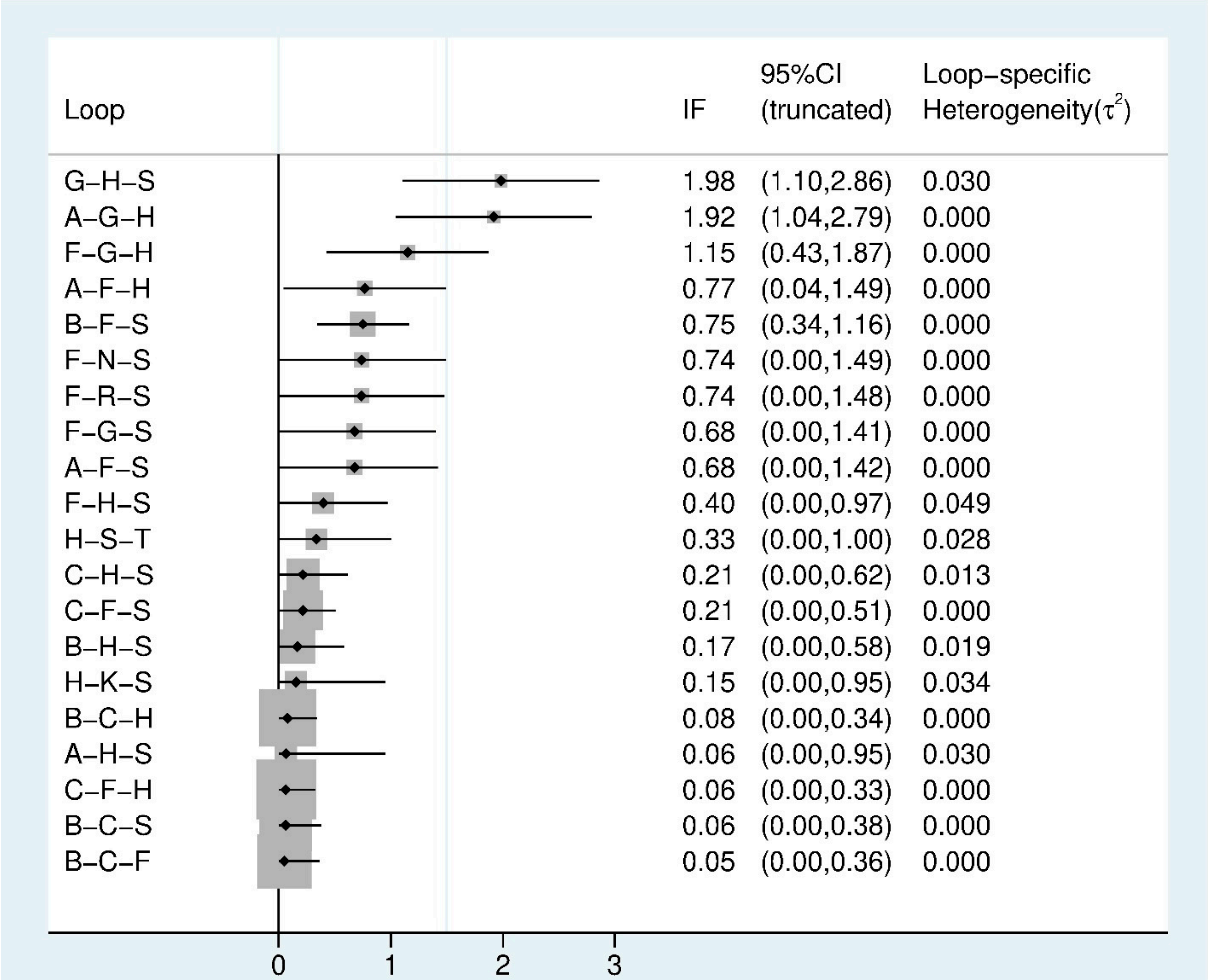

A: CGM (glucosamine+Curcumin derivatives); B: CS (chondroitin sulfate); C: Celecoxib; F: G (glucosamine); G: G+CGM (glucosamine+Curcumin derivatives); H: G+CS (glucosamine+chondroitin sulfate); K: G+CS+MSM (glucosamine+chondroitin sulfate+manganese ascorbate); N: G+MSM (glucosamine+methylsulfonylmethane); R: MSM (methylsulfonylmethane); S (reference): Placebo; T: UC-II (undenatured type II collagen).

Figure S10. Inconsistency factors for short-term pain of 20 trials.

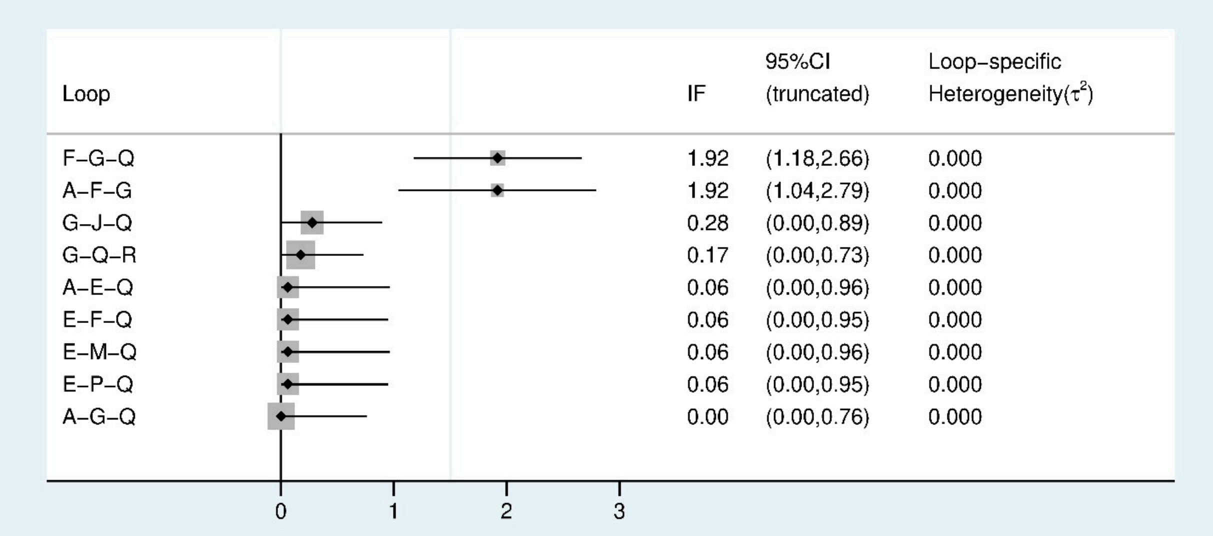

A: CGM (curcumin derivatives); E: G (glucosamine); F: G+CGM (glucosamine+curcumin derivatives); G: G+CS (glucosamine+chondroitin sulfate); J: G+CS+MSM (glucosamine+chondroitin sulfate+methylsulfonylmethane); M: G+MSM (glucosamine+methylsulfonylmethane); P: MSM; Q: Placebo; R: UC-II (undenatured type II collagen).

Figure S11. Inconsistency factors for long-term pain of 9 trials.

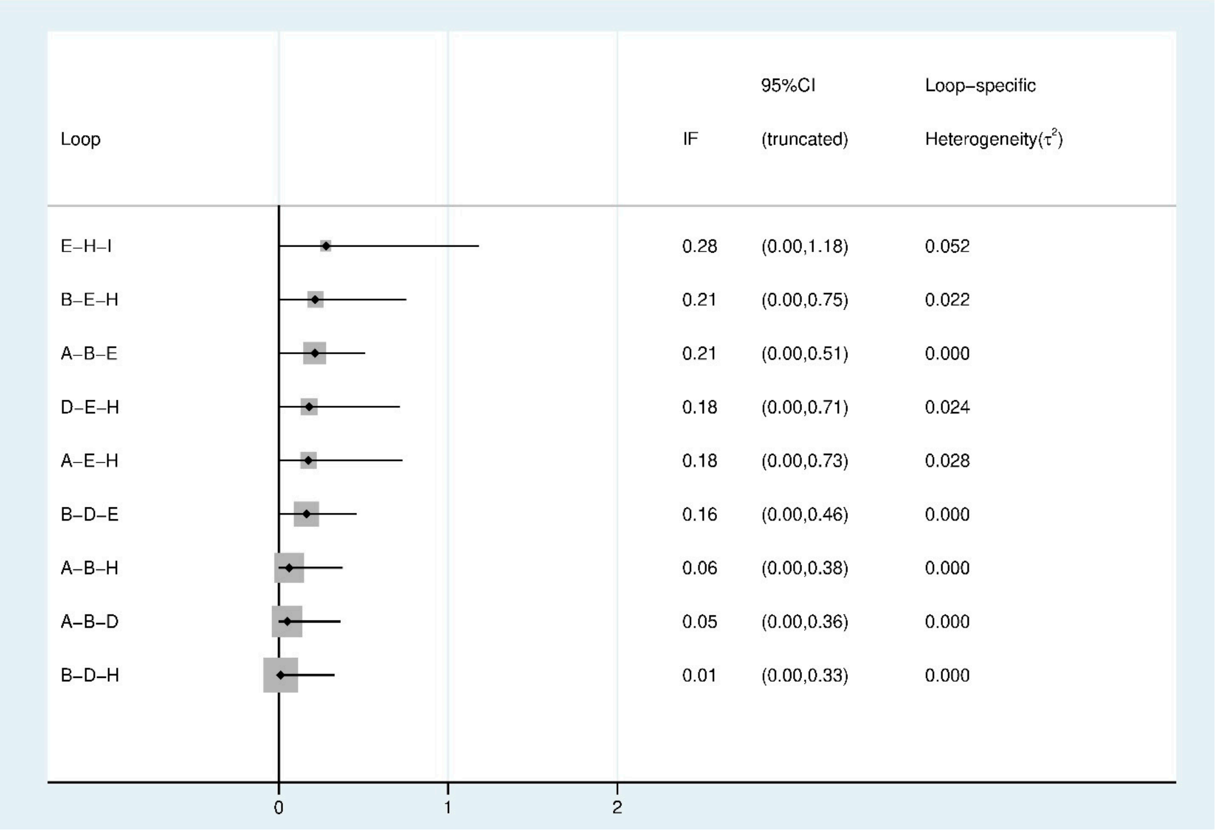

A: CS (chondroitin sulfate); B: Celecoxib; D: G (glucosamine); E: G+CS (glucosamine+chondroitin sulfate); H: Placebo; I: UC-II (undenatured type II collagen).

Sensitivity analysis to resolve the inconsistency

Figure S12. Sensitivity analysis to resolve the inconsistency for overall pain.

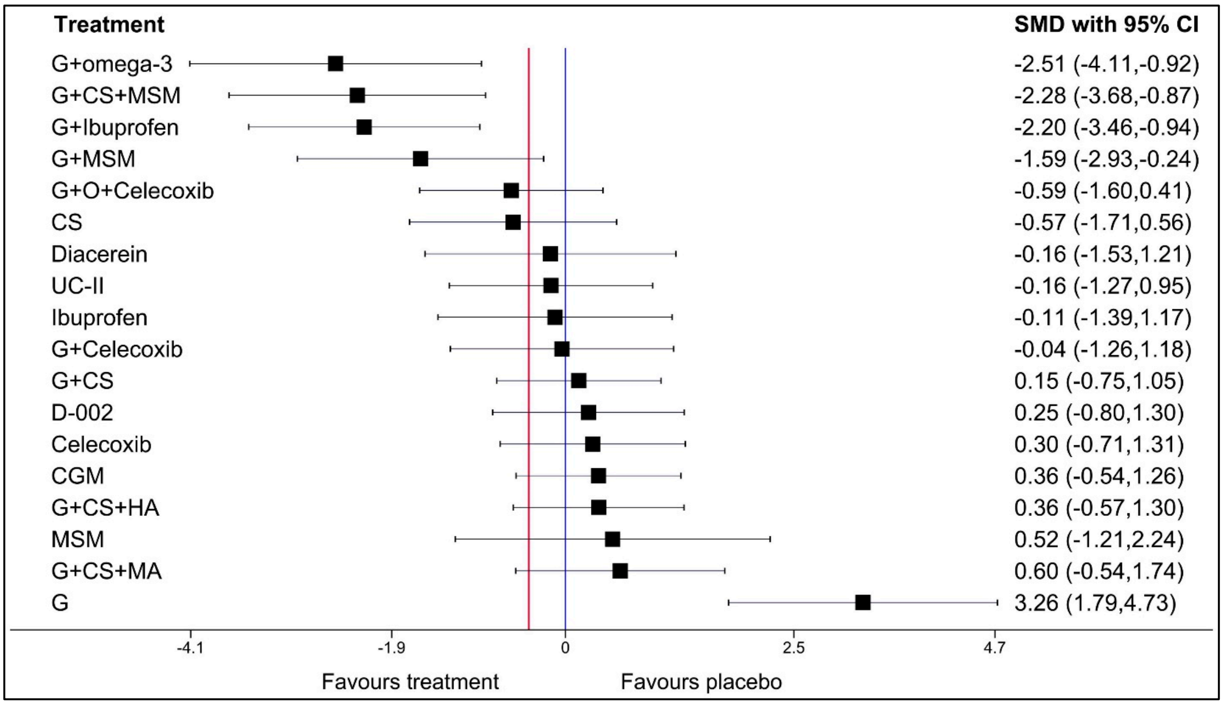

UC-II, undenatured type II collagen; MSM, methylsulfonylmethane; G+omega-3, glucosamine+omega-3; G+O+Celecoxib, glucosamine+ozone+celecoxib; G+MSM, glucosamine+methylsulfonylmethane; G+Ibuprofen, glucosamine+ibuprofen; G+Celecoxib, glucosamine+celecoxib; G+CS+MSM, glucosamine+chondroitin sulfate+methylsulfonylmethane; G+CS+MA, glucosamine+chondroitin sulfate+manganese ascorbate; G+CS+HA, glucosamine+chondroitin sulfate+hyaluronic acid; G+CS; glucosamine+chondroitin sulfate; G, glucosamine; D-002, inhibitor of both cyclooxygenase and 5-lipoxygenase activities; D-002, inhibitor of both cyclooxygenase and 5-lipoxygenase activities; CGM, curcumin derivatives.

Figure S13. Sensitivity analysis to resolve the inconsistency for short-term pain.

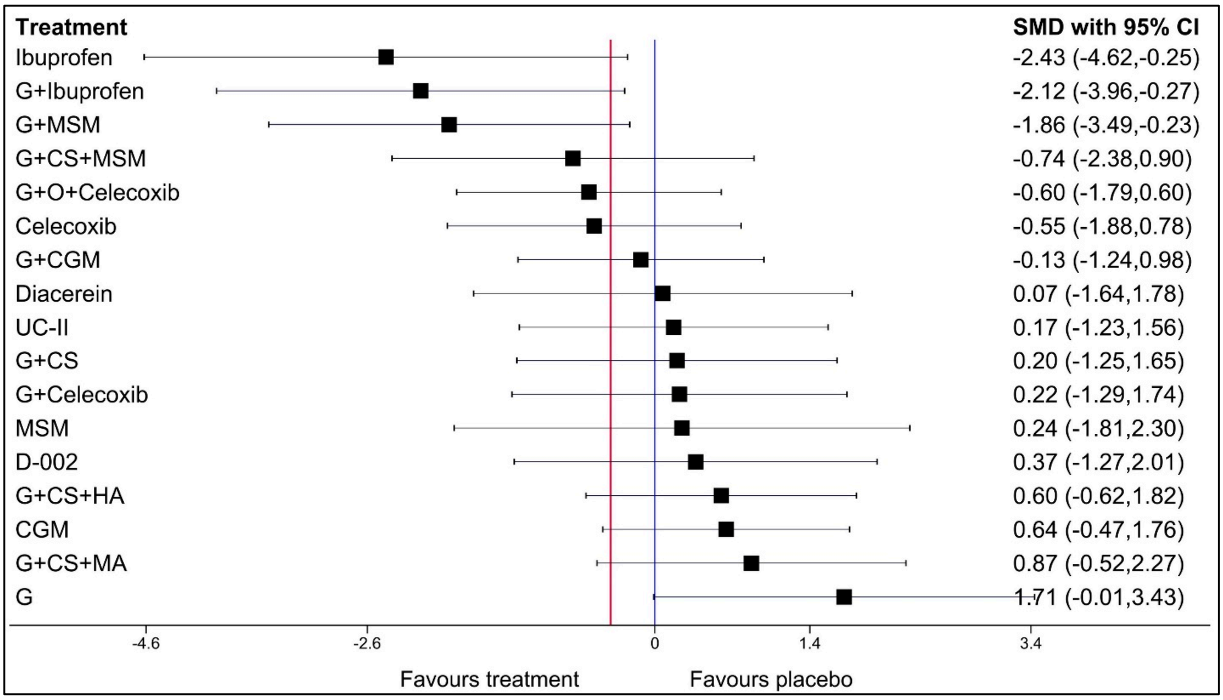

UC-II, undenatured type II collagen; MSM, methylsulfonylmethane; G+O+Celecoxib, glucosamine+ozone+celecoxib; G+MSM, glucosamine+methylsulfonylmethane; G+Ibuprofen, glucosamine+ibuprofen; G+Celecoxib, glucosamine+celecoxib; G+CS+MSM, glucosamine+chondroitin sulfate+methylsulfonylmethane; G+CS+MA, glucosamine+chondroitin sulfate+manganese ascorbate; G+CS+HA, glucosamine+chondroitin sulfate+hyaluronic acid; G+CS; glucosamine+chondroitin sulfate; G, glucosamine; D-002, inhibitor of both cyclooxygenase and 5-lipoxygenase activities; D-002, inhibitor of both cyclooxygenase and 5-lipoxygenase activities; CGM, curcumin derivatives.

Results of adverse events

Figure S14. Network plot for adverse events.

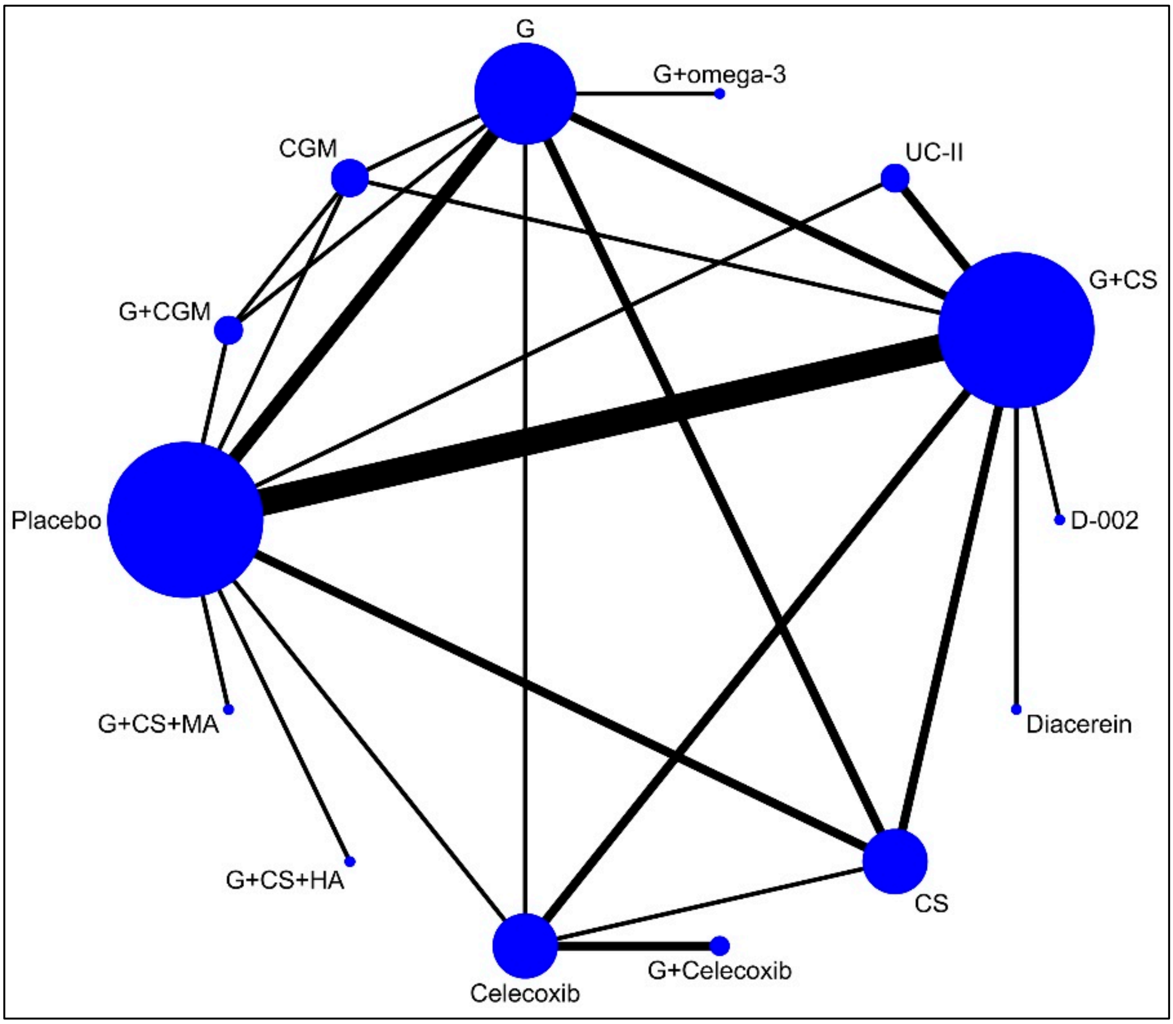

UC-II, undenatured type II collagen; MSM, methylsulfonylmethane; G+omega-3, glucosamine+omega-3; G+O+Celecoxib, glucosamine+ozone+celecoxib; G+MSM, glucosamine+methylsulfonylmethane; G+Ibuprofen, glucosamine+ibuprofen; G+Celecoxib, glucosamine+celecoxib; G+CS+MSM, glucosamine+chondroitin sulfate+methylsulfonylmethane; G+CS+MA, glucosamine+chondroitin sulfate+manganese ascorbate; G+CS+HA, glucosamine+chondroitin sulfate+hyaluronic acid; G+CS; glucosamine+chondroitin sulfate; G, glucosamine; D-002, inhibitor of both cyclooxygenase and 5-lipoxygenase activities; D-002, inhibitor of both cyclooxygenase and 5-lipoxygenase activities; CGM, curcumin derivatives.

Figure S15. Interval plot for adverse events.

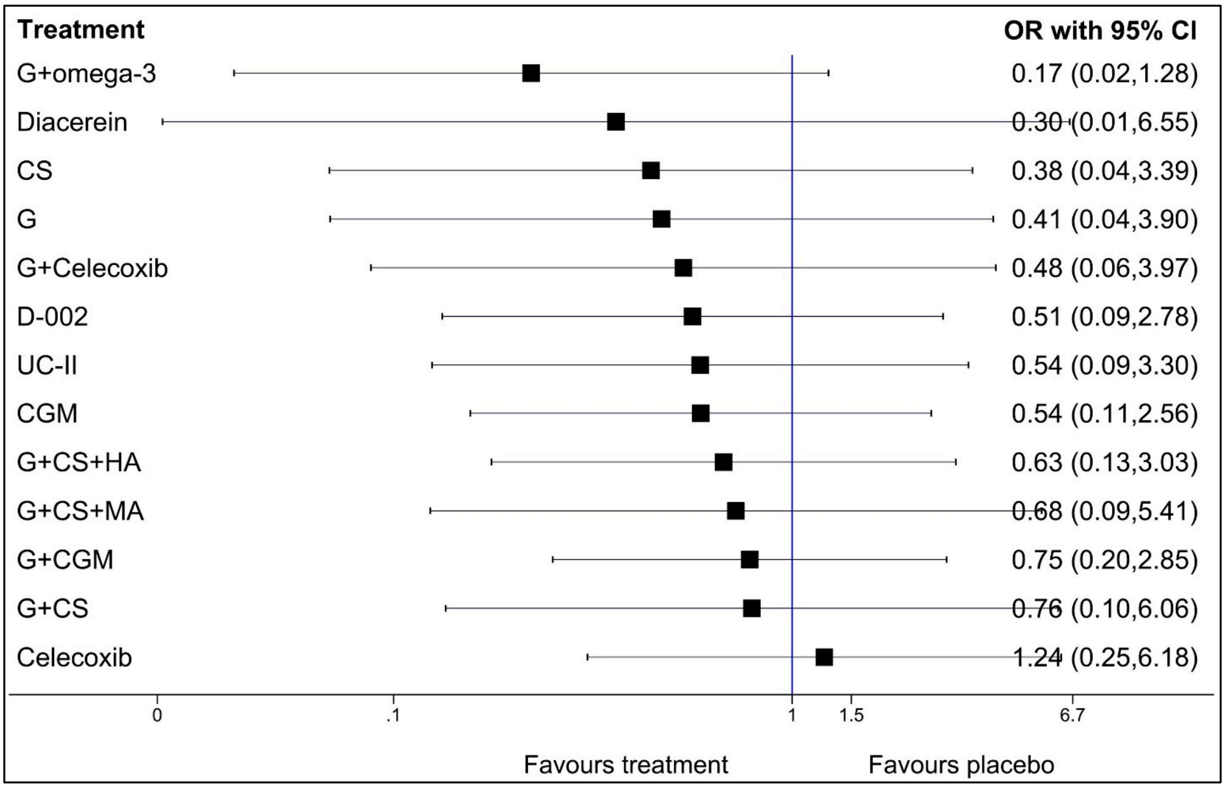

UC-II, undenatured type II collagen; MSM, methylsulfonylmethane; G+omega-3, glucosamine+omega-3; G+O+Celecoxib, glucosamine+ozone+celecoxib; G+MSM, glucosamine+methylsulfonylmethane; G+Ibuprofen, glucosamine+ibuprofen; G+Celecoxib, glucosamine+celecoxib; G+CS+MSM, glucosamine+chondroitin sulfate+methylsulfonylmethane; G+CS+MA, glucosamine+chondroitin sulfate+manganese ascorbate; G+CS+HA, glucosamine+chondroitin sulfate+hyaluronic acid; G+CS; glucosamine+chondroitin sulfate; G, glucosamine; D-002, inhibitor of both cyclooxygenase and 5-lipoxygenase activities; D-002, inhibitor of both cyclooxygenase and 5-lipoxygenase activities; CGM, curcumin derivatives

Figure S16. SUCRA ranking for adverse events.

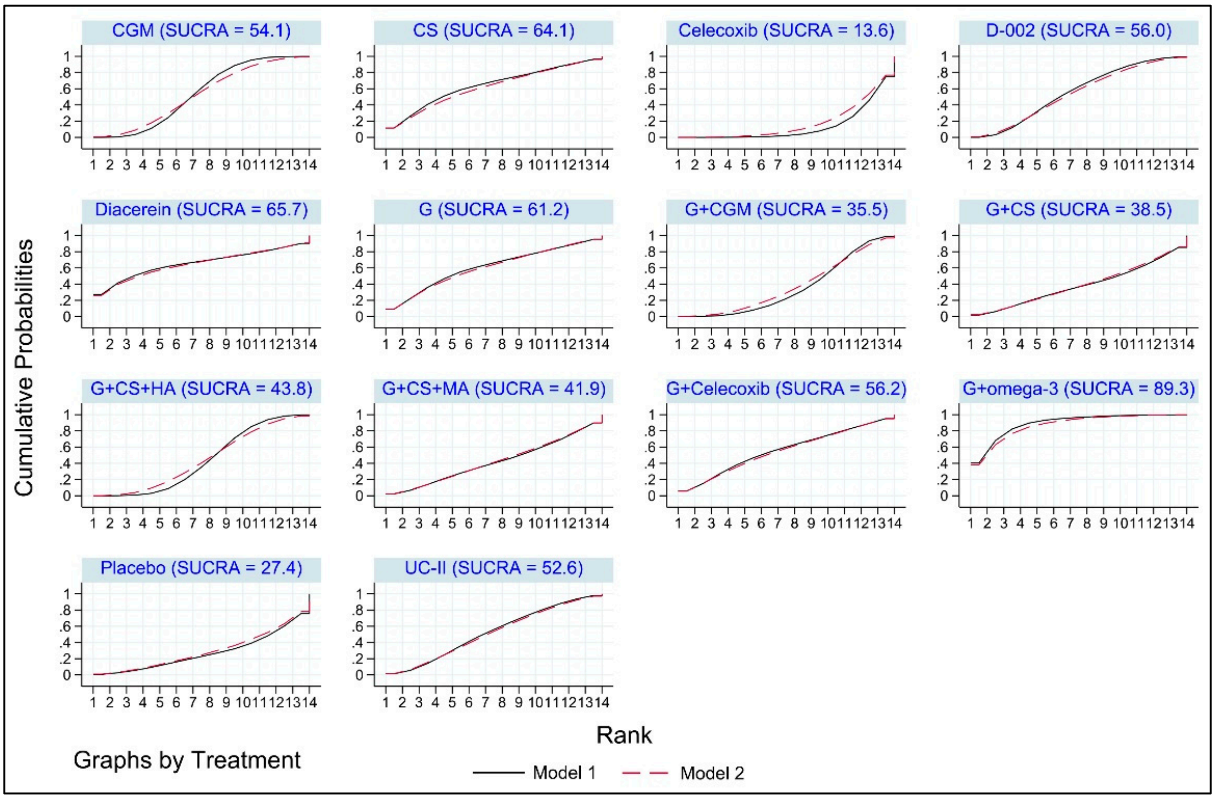

UC-II, undenatured type II collagen; MSM, methylsulfonylmethane; G+omega-3, glucosamine+omega-3; G+O+Celecoxib, glucosamine+ozone+celecoxib; G+MSM, glucosamine+methylsulfonylmethane; G+Ibuprofen, glucosamine+ibuprofen; G+Celecoxib, glucosamine+celecoxib; G+CS+MSM, glucosamine+chondroitin sulfate+methylsulfonylmethane; G+CS+MA, glucosamine+chondroitin sulfate+manganese ascorbate; G+CS+HA, glucosamine+chondroitin sulfate+hyaluronic acid; G+CS; glucosamine+chondroitin sulfate; G, glucosamine; D-002, inhibitor of both cyclooxygenase and 5-lipoxygenase activities; D-002, inhibitor of both cyclooxygenase and 5-lipoxygenase activities; CGM, curcumin derivatives.

Evaluation of transitivity for adverse events

In network meta-analysis, transitivity refers to the assumption that the relative effect between treatment A and treatment B can be validly compared indirectly through one or more intermediate treatments. To evaluate this, we show boxplots for the distributions of two potential effect modifiers: mean age and percentage of females across the available direct comparisons. If no important differences in the distributions of effect modifiers are found, the transitivity assumption is considered to hold in the network.

Figure S17. Assessment of transitivity in terms of mean age distribution in the network of interventions for adverse events outcome.

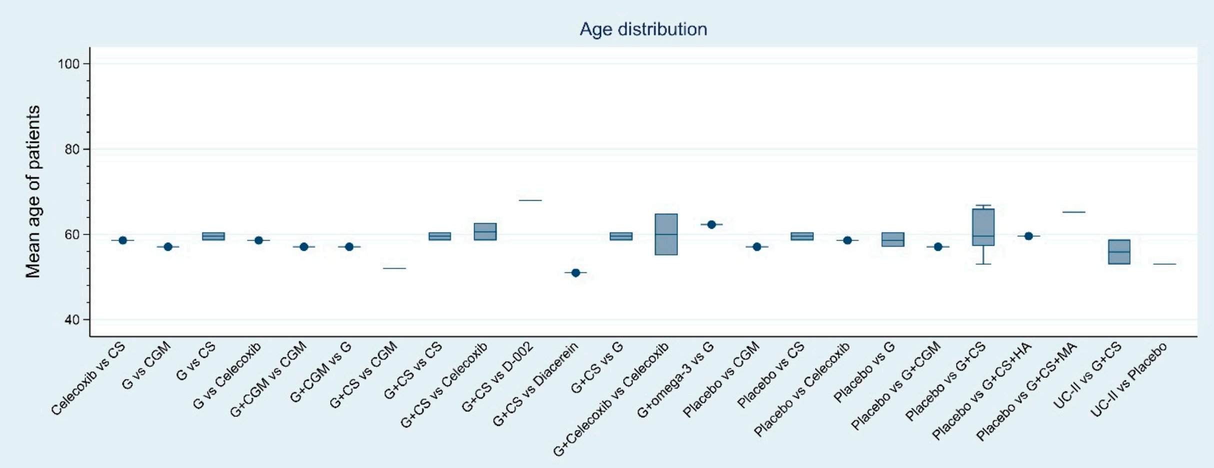

Figure S18. Assessment of transitivity in terms of percentage of females in the network of interventions for adverse events outcome.

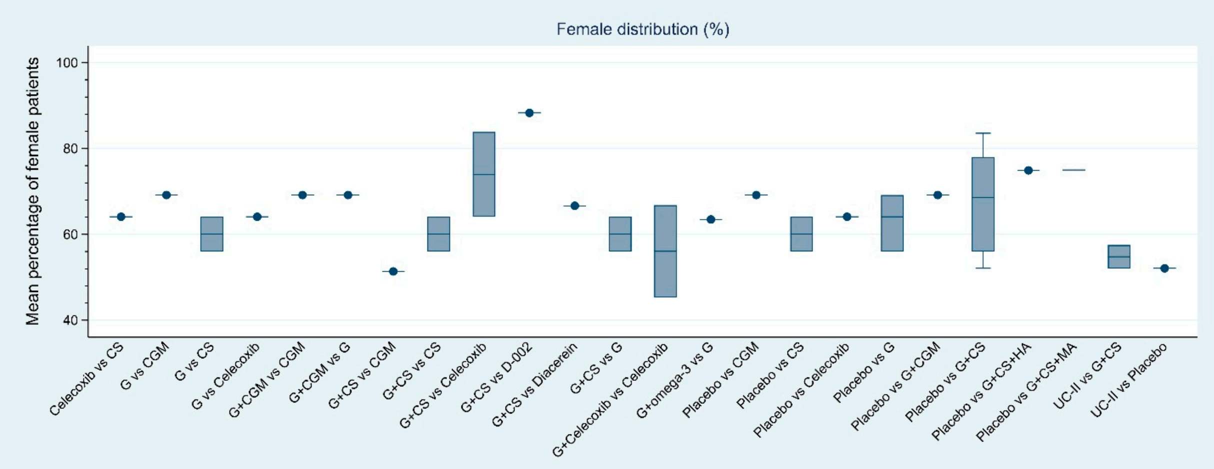

Figure S19. Inconsistency factors for overall pain of all trials.

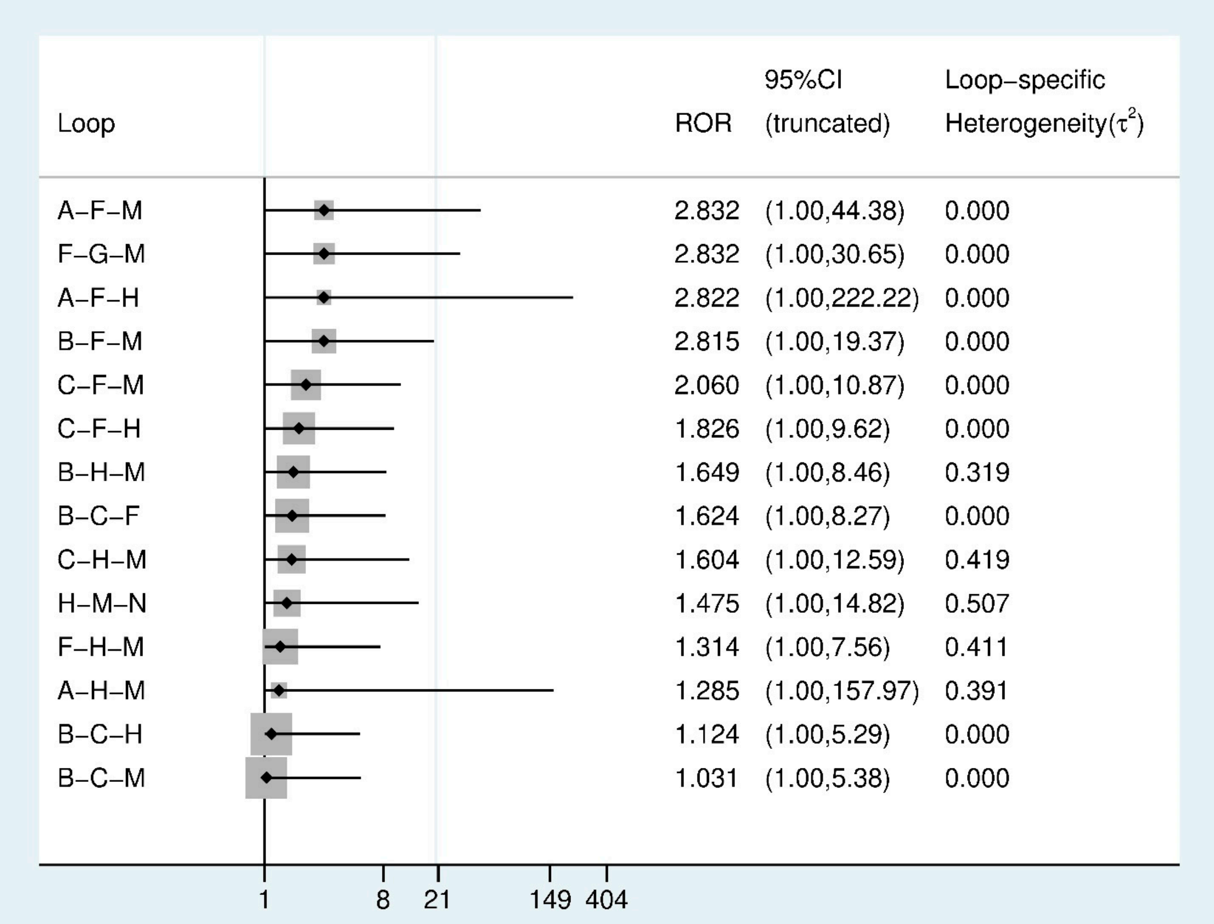

We used a loop-specific approach to investigate the consistency within every closed triangular or quadratic loop in every network as the difference between direct and indirect estimates for a specific treatment comparison (RoR - ratio of odds ratio) in the loop. We identified inconsistent loops as those yielding a 95% CI excluding 1.

Comparison-adjusted funnel plot for primary and secondary outcomes

Figure S20. Comparison adjusted funnel plot for overall pain.

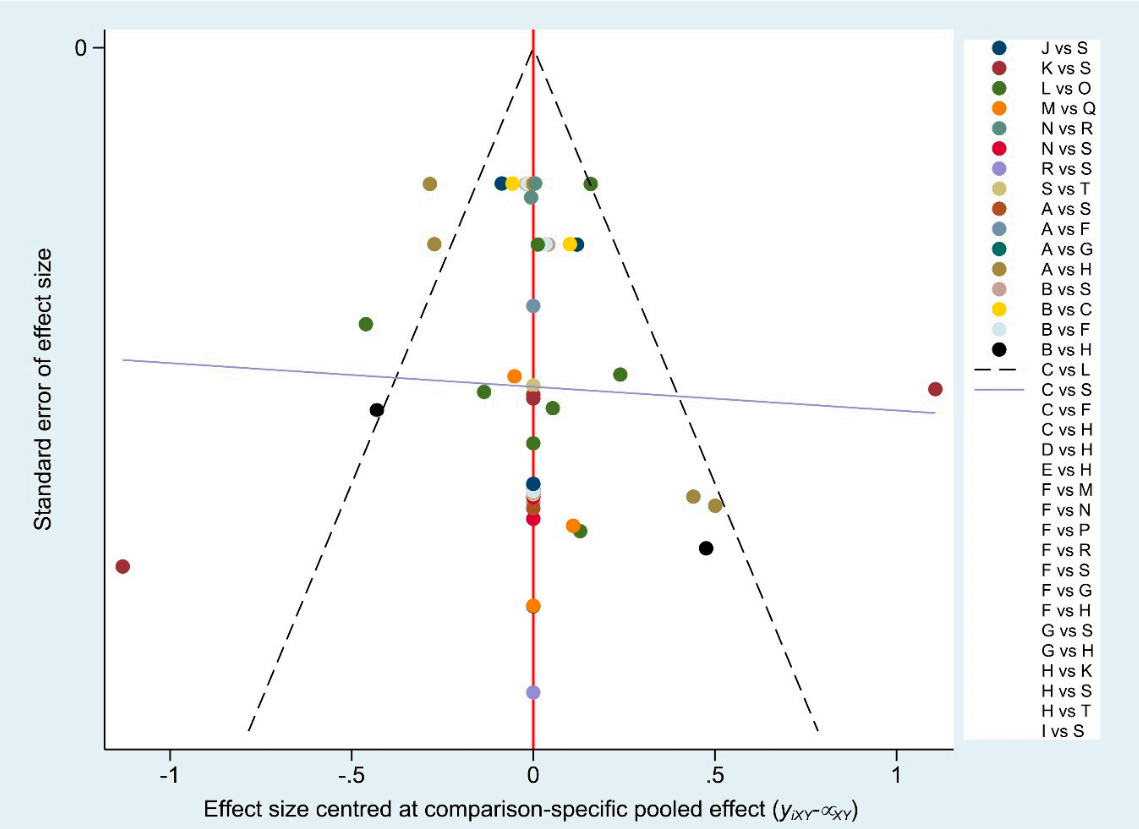

A: CGM; B: CS (curcumin derivatives); C: Celecoxib; D: D-002 (inhibitor of both cyclooxygenase and 5-lipoxygenase activities); E: Diacerein; F: G (glucosamine); G: G+CGM (glucosamine+curcumin derivatives); H: G+CS (glucosamine+chondroitin sulfate); I: G+CS+HA (glucosamine+chondroitin sulfate+hyaluronic acid); J: G+CS+MA (glucosamine+chondroitin sulfate+manganese ascorbate); K: G+CS+MSM (glucosamine+chondroitin sulfate+methylsulfonylmethane); L: G+Celecoxib (glucosamine+celecoxib); M: G+Ibuprofen (glucosamine+ibuprofen); N: G+MSM (glucosamine+methylsulfonylmethane); O: G+O+Celecoxib (glucosamine+ozone+celecoxib); P: G+omega-3 (glucosamine+omega-3); Q: Ibuprofen; R: MSM (methylsulfonylmethane); S: Placebo; T: UC-II (undenatured type II collagen).

Figure S21. Comparison adjusted funnel plot for short-term pain.

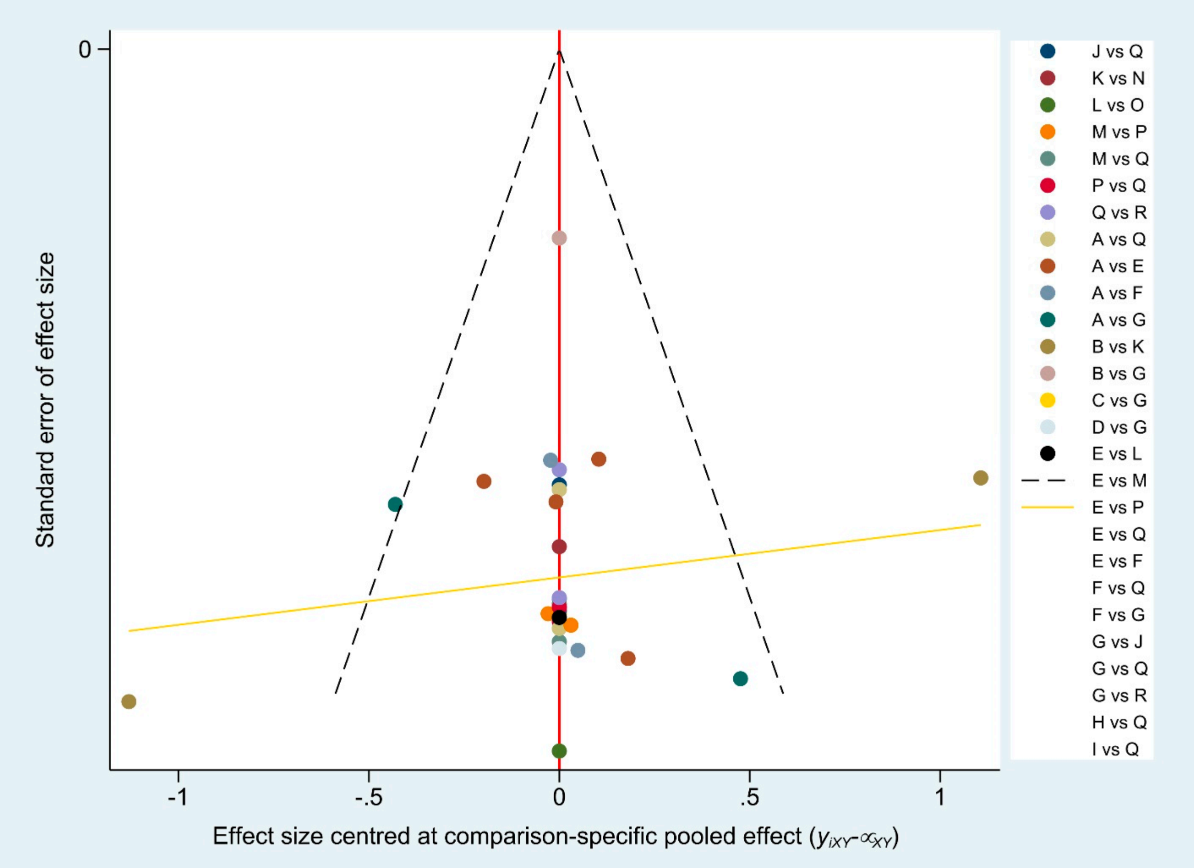

A: CGM (curcumin derivatives); B: Celecoxib; C: D-002 (inhibitor of both cyclooxygenase and 5-lipoxygenase activities); D: Diacerein; E: G (glucosamine); F: G+CGM (glucosamine+curcumin derivatives); G: G+CS (glucosamine+chondroitin sulfate); H: G+CS+HA (glucosamine+chondroitin sulfate+hyaluronic acid); I: G+CS+MA (glucosamine+chondroitin sulfate+manganese ascorbate); J: G+CS+MSM (glucosamine+chondroitin sulfate+methylsulfonylmethane); K: G+Celecoxib (glucosamine+celecoxib); L: G+Ibuprofen (glucosamine+ibuprofen); M: G+MSM (glucosamine+methylsulfonylmethane); N: G+O+Celecoxib (glucosamine+ozone+celecoxib); O: Ibuprofen; P: MSM (methylsulfonylmethane); Q: Placebo; R: UC-II (undenatured type II collagen).

Figure S22. Comparison adjusted funnel plot for long-term pain.

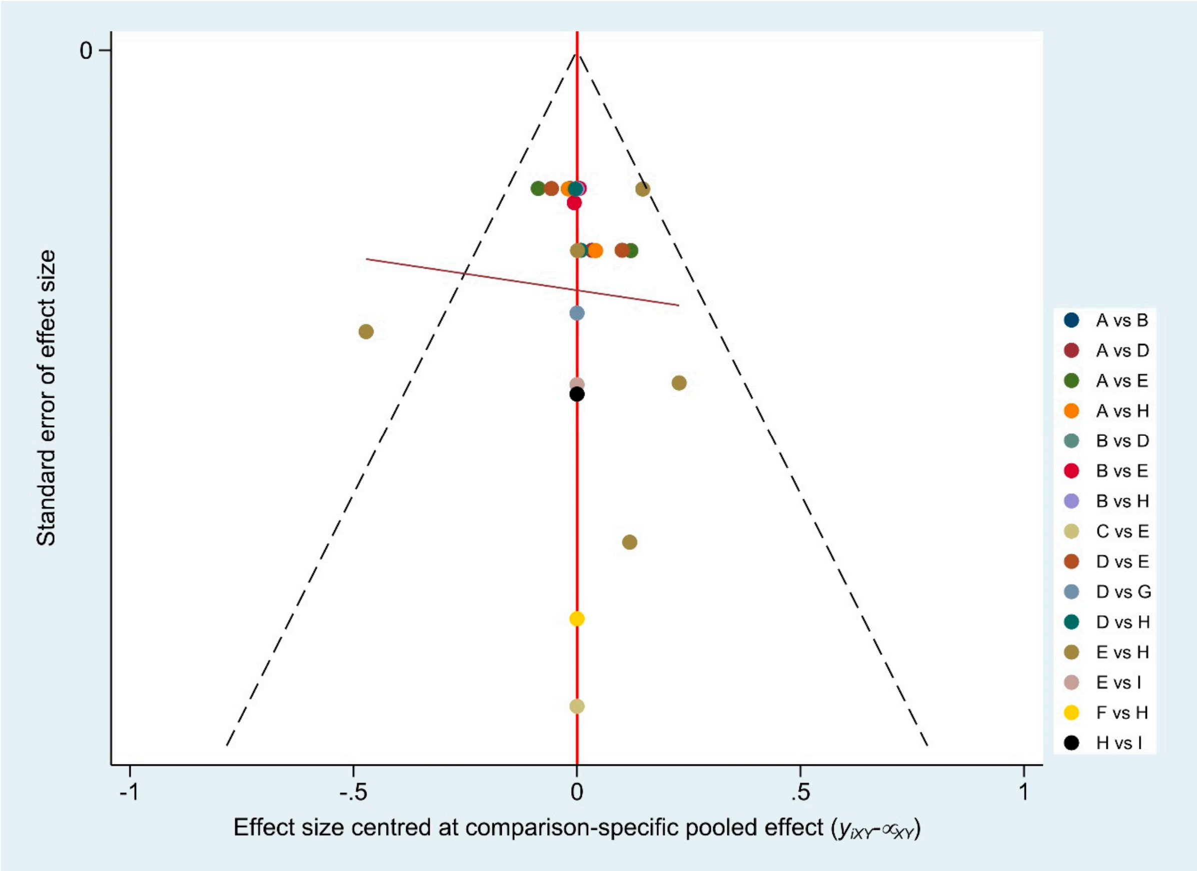

A: CS (chondroitin sulfate); B: Celecoxib; C: Diacerein; D: G (glucosamine); E: G+CS (glucosamine+chondroitin sulfate); F: G+CS+MA (glucosamine+chondroitin sulfate+manganese ascorbate); G: G+omega-3 (glucosamine+omega-3); H: Placebo; I: UC-II (undenatured type II collagen).

Figure S23. Comparison adjusted funnel plot for adverse events.

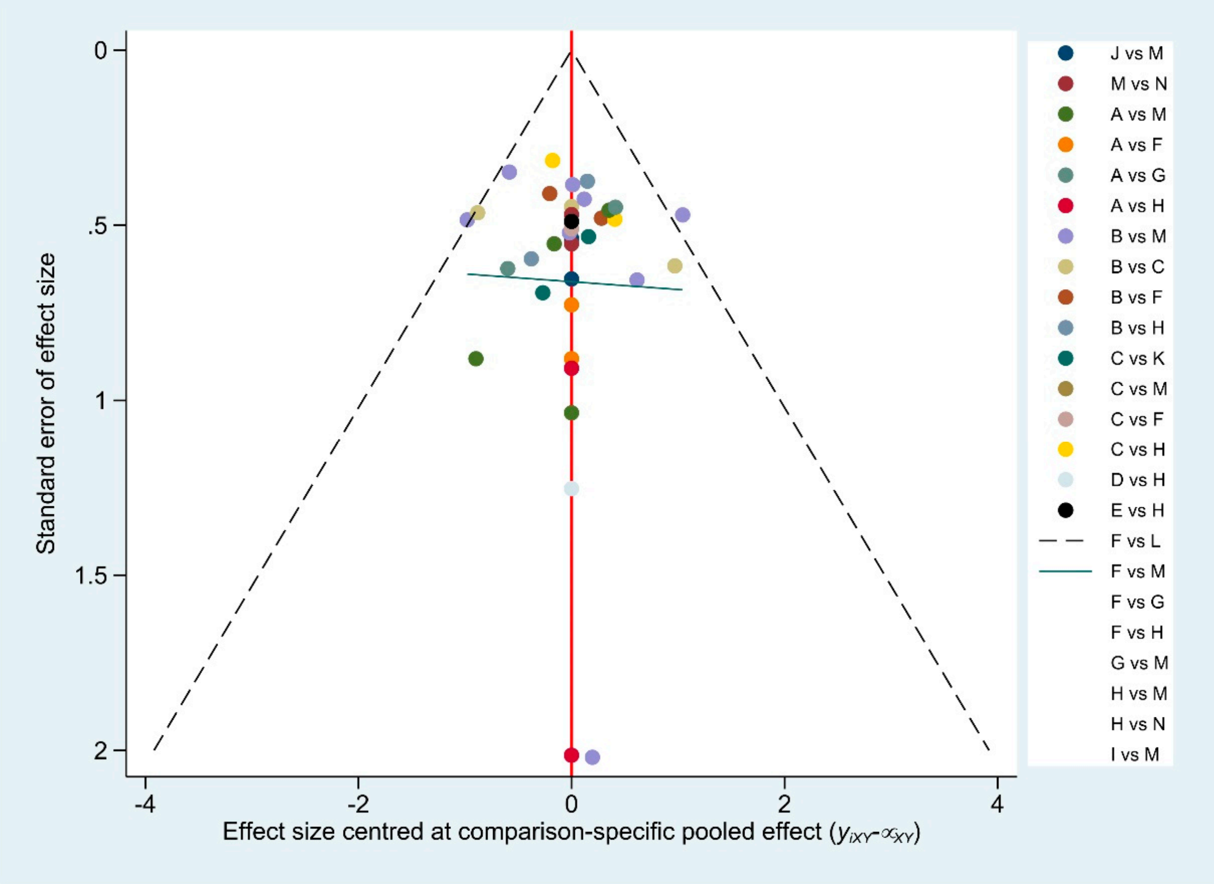

A: CGM (curcumin derivatives); B: CS (curcumin derivatives); C:Celecoxib; D:D-002 (inhibitor of both cyclooxygenase and 5-lipoxygenase activities); E:Diacerein; F:G (glucosamine); G:G+CGM (glucosamine+curcumin derivatives); H:G+CS (glucosamine+chondroitin sulfate); I:G+CS+HA (glucosamine+chondroitin sulfate+hyaluronic acid); J:G+CS+MA (glucosamine+chondroitin sulfate+manganese ascorbate); K:G+Celecoxib (glucosamine+celecoxib); L:G+omega-3 (glucosamine+omega-3); M (reference): Placebo; N: UC-II (undenatured type II collagen).

Figure S24. Sensitivity analysis of network meta-analysis for overall pain and short-term pain.

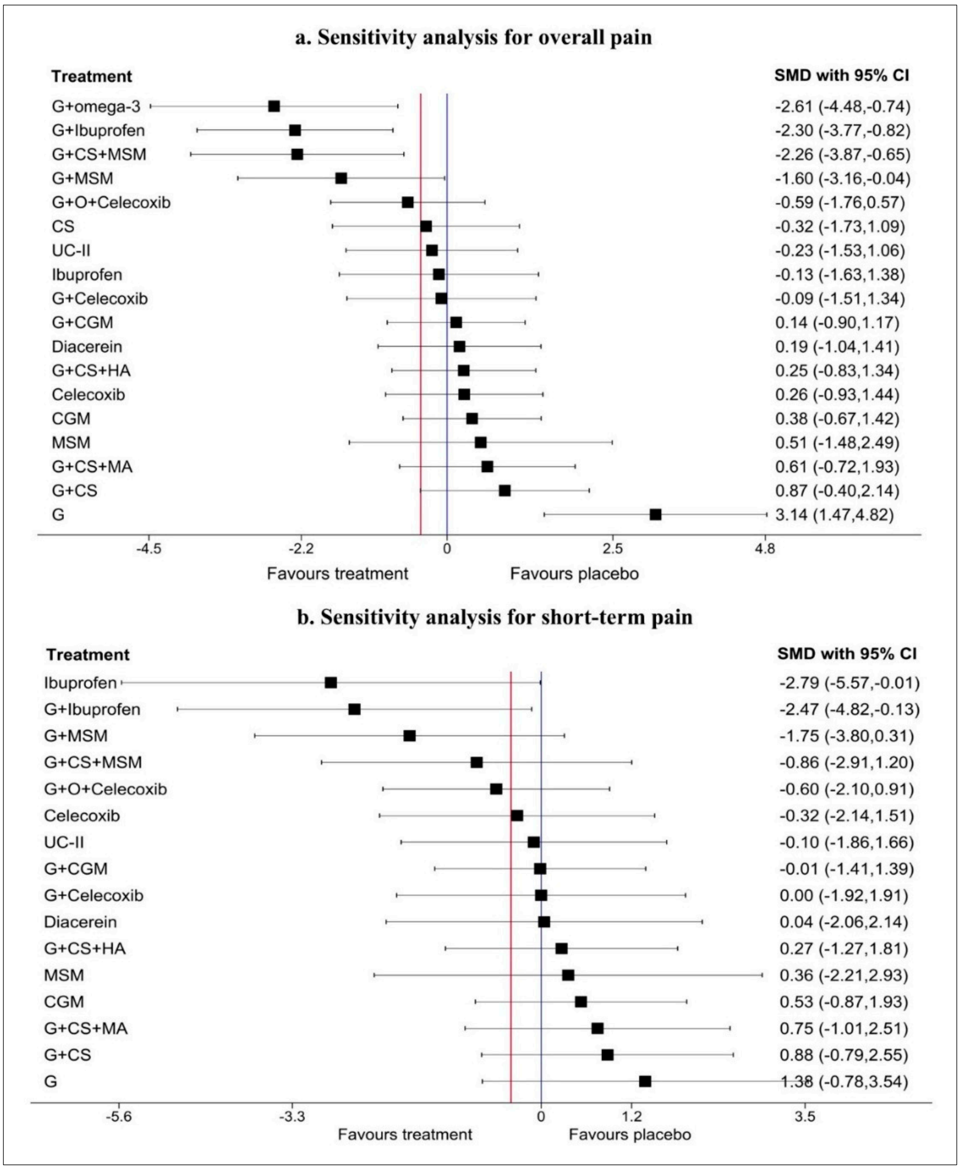

(a) overall pain and (b) short-term pain compared with placebo, ordered according to treatment effect size. The purple vertical line represents the line of no effect, the red vertical line indicates the MCID, and the black square represents the effect size. When the 95% CI crosses the MCID line, it indicates that the effectiveness of the intervention is uncertain. MCID, minimum clinically important difference; SMD, standardized mean difference; 95% CI, 95% confidence interval; G+omega-3, glucosa-mine+omega-3; G+Ibuprofen, glucosamine+ibuprofen; G+CS+MSM, glucosamine+chondroitin sulfate+methylsulfonylmethane; G+MSM, glucosamine+methylsulfonylmethane; G+O+Celecoxib, glucosamine+ozone+celecoxib; CS, chondroitin sulfate; UC-II, undenatured type II collagen; G+Celecoxib, glucosamine+celecoxib; D-002, an inhibitor of both cyclooxygenase and 5-lipoxygenase activitiy; G+CS+HA, glucosamine+chondroitin sulfate+hyaluronic acid; CGM, curcumin derivatives; MSM, methylsulfonylmethane; G+CS+MA, glucosamine+chondroitin sul-fate+manganese ascorbate; G+CS; glucosamine+chondroitin sulfate; G, glucosamine.
